# Carotenoids modulate kernel texture in maize by influencing amyloplast envelope integrity

**DOI:** 10.1038/s41467-020-19196-9

**Published:** 2020-10-22

**Authors:** Haihai Wang, Yongcai Huang, Qiao Xiao, Xing Huang, Changsheng Li, Xiaoyan Gao, Qiong Wang, Xiaoli Xiang, Yidong Zhu, Jiechen Wang, Wenqin Wang, Brian A. Larkins, Yongrui Wu

**Affiliations:** 1 National Key Laboratory of Plant Molecular Genetics, CAS Center for Excellence in Molecular Plant Sciences, Shanghai Institute of Plant Physiology & Ecology, Shanghai, 200032 China; 2 University of the Chinese Academy of Sciences, Beijing, 100049 China; 3 School of Agriculture and Biology, Shanghai Jiao Tong University, Shanghai, 200240 China; 4 School of Plant Sciences, University of Arizona, Tucson, Arizona 85721 USA

**Keywords:** Agricultural genetics, Agricultural genetics, Natural variation in plants, Plant breeding

## Abstract

The mechanism that creates vitreous endosperm in the mature maize kernel is poorly understood. We identified *Vitreous endosperm 1* (*Ven1*) as a major QTL influencing this process. *Ven1* encodes β-carotene hydroxylase 3, an enzyme that modulates carotenoid composition in the amyloplast envelope. The A619 inbred contains a nonfunctional *Ven1* allele, leading to a decrease in polar and an increase in non-polar carotenoids in the amyloplast. Coincidently, the stability of amyloplast membranes is increased during kernel desiccation. The lipid composition in endosperm cells in A619 is altered, giving rise to a persistent amyloplast envelope. These changes impede the gathering of protein bodies and prevent them from interacting with starch grains, creating air spaces that cause an opaque kernel phenotype. Genetic modifiers were identified that alter the effect of *Ven1*^*A619*^, while maintaining a high β-carotene level. These studies provide insight for breeding vitreous kernel varieties and high vitamin A content in maize.

## Abstract (web summary)

Very little is known about how vitreous endosperm in the mature maize kernel is created. Here, via map-based cloning, the authors find that mutation of a β-carotene hydroxylase 3 encoding gene *Ven1* affects carotenoids and lipids composition, which consequently influences amyloplast envelope integrity.

## Introduction

Maize kernel texture is a critical agronomic trait. It is influenced by the ratio of vitreous endosperm in the outer part of the kernel to the starchy endosperm in the center of the kernel^1^. Vitreous endosperm strengthens the kernel and protects the grains from mechanical damages during harvesting and shipping, whereas starchy endosperm is breakable and fragile, and is susceptible to pests and diseases^2^. Even though teosinte (wild maize) has a hard glume encasing the seed, it contains a large portion of vitreous endosperm. In this respect, there may have been evolutionary selection for vitreous endosperm in teosinte to enhance kernel integrity and passage through the gut of birds and other animals to aid seed dispersal. Maize grains containing a greater amount of vitreous endosperm have a higher kernel density and test weight and lower flotation indices^3,4^. The vitreous region is also important for food processing, including making of grits and cornflakes, and other food products^5^, and it is essential for making popcorn pop.

What creates vitreous endosperm is a longstanding question with many hypotheses, most of which are associated with synthesis of starch and storage proteins, and the factors affecting them. The major storage metabolites in the endosperm are starch and proteins, which account for 70 and 10%, respectively, of the endosperm dry mass. Starch is synthesized and accumulated as starch grains (SGs) in amyloplasts. The major proteins are storage proteins, prolamins, collectively called zeins. Zeins contain a signal peptide that directs their synthesis into the lumen of the rough endoplasmic reticulum (RER), where they become organized into accretions called protein bodies (PBs)^6,7^. During kernel maturation, SGs become surrounded by a proteinaceous matrix composed of PBs and amorphous cytoplasmic proteins. As the seed undergoes desiccation, the integrity of organellar membranes is lost and the peripheral endosperm cells, filled predominantly with PBs and SGs, form vitreous endosperm, while central endosperm cells, containing primarily SGs and few PBs, form the starchy endosperm^1^. Although a large number of mutations affecting zein proteins and starch synthesis have been identified and provided insight into the mechanisms that create vitreous endosperm^8,9^, little is known about quantitative trait loci and their metabolic products that explain the genetic variation in kernel texture found in natural populations.

Maize inbred lines have great natural variation in kernel texture, ranging from nearly completely vitreous to completely opaque. W64A and A619 are typical yellow dent inbred lines that illustrate the natural variation for a normal amount and a small amount of vitreous endosperm. In this work, through backcrossing, we are able to identify and clone the gene responsible for a major QTL, *Ven1*, which affects kernel texture by regulating the β-carotene content. High levels of β-carotene appear to prevent the breakdown of amyloplast membranes and affect the quantity and composition of lipids in desiccating endosperm cells. This disrupts interactions between SGs and PBs, leading to air spaces and, consequently, opacity of the mature endosperm.

## Results

### *Ven1* is a principal QTL controlling maize kernel texture

**Fig. 1:**
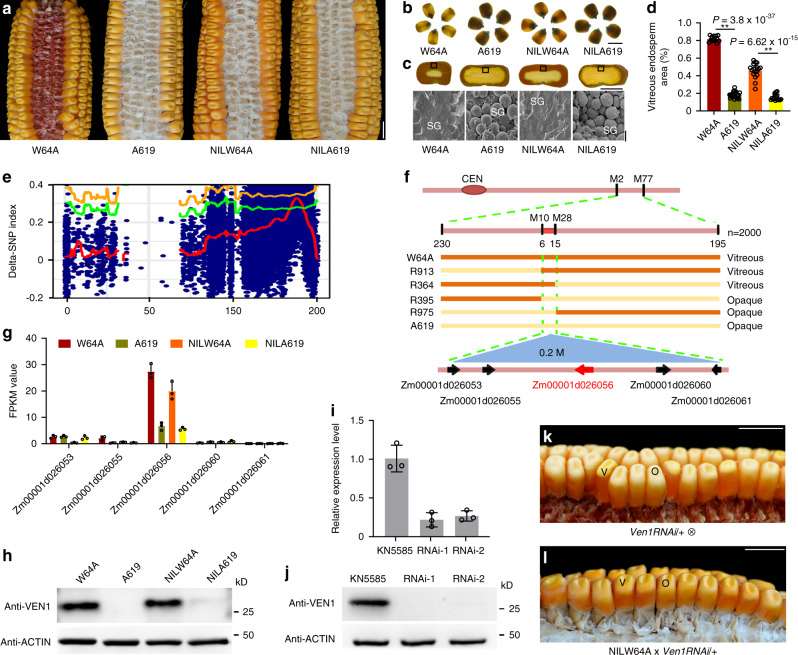
Map-based cloning and genetic verification of *Ven1*. **a** Ear phenotypes of W64A, A619, NILW64A and NILA619. Scale bar, 1 cm. **b** Kernel vitreousness as observed on the light box. W64A and NILW64A kernels are vitreous, and A619 and NILA619 opaque. Scale bar, 1 cm. **c** Kernel transverse sections of W64A, A619, NILW64A and NILA619. Top panel, the sections observed under the natural light. Scale bar, 5 mm; bottom panel, the areas boxed in the top observed by SEM. Scale bar, 10 μm. SG, starch grain. **d** Measurement of the vitreous endosperm area in kernel transverse sections. Data are presented as mean values ± SD, *n* = 15, 23, 18 and 16, respectively. **, significant differences at *P* < 0.01 in two-sided Student’s *t*-test. **e** Mapping-by-sequencing of *Ven1*. Delta SNP index of two pools of vitreous and opaque kernels segregated from F_1_BC_5_ indicated a candidate QTL region in the long arm of chromosome 10. The red line is the mean value of SNP-index, green is the threshold line of 95% confidence level, and orange is 99% confidence level. **f** Fine mapping of *Ven1*. *Ven1* was located in a 0.2 Mb region between markers M10 and M28, containing five genes. **g** Expression level of the candidate genes in 18-DAP endosperms of two parents and two NILs. The values are shown as the means ± sd of three biological replicates. **h** Immunoblotting analysis of VEN1 in seeds. ACTIN was used as an internal control. **i** qRT-PCR analysis of *Ven1* in two independent transgenic lines. The values are shown as the means ± s.d. of three biological replicates. *Actin* was used as an internal control. **j** Immunoblotting analysis of VEN1 in 18-DAP endosperms of two independent transgenic lines. ACTIN was used as an internal control. **k** Segregation of opaque kernels in a self-pollinated *Ven1RNAi*/*+* ear. **l** Ear phenotype of NILW64A x *Ven1RNAi*/+. Vitreous and opaque kernels segregated equally. **k**, **l** V, vitreous kernel; O, opaque kernel. Scale bar, 1 cm. The source data underlying **d, g–j** are provided as a Source Data file.

Maize inbred lines W64A and A619 display differences in endosperm vitreousness (Fig. 1a–d). When measured, W64A kernels contain 80% vitreous endosperm, whereas A619 kernels have only 20% (Fig. 1c, d). On a light box, light transmission was observed for W64A but not A619 kernels (Fig. 1b). Scanning electron microscopy (SEM) revealed that the vitreous region of W64A endosperm contained SGs embedded in a protein matrix, but this matrix was not apparent in A619, which had loosely compacted SGs (Fig. 1c).

We used A619 to make reciprocal crosses with W64A and two other vitreous inbred lines, D1051 and P25, and found that all the F1 hybrids were vitreous (Supplementary Fig. 1), indicating the major QTL (designated *vitreous endosperm 1*, *Ven1*) responsible for the opaque endosperm phenotype in A619 (*Ven1*^*A619*^) is recessive. Maize kernel texture is a complex trait and is affected by many genetic and non-genetic factors. To clone *Ven1*, we created populations by continuously introgressing the dominant *Ven1*^*W64A*^ allele into the A619 background via backcrossing. Progeny from the first two rounds of backcrossing (F_1_BC_1_ and F_1_BC_2_) did not show phenotypic segregation, but in the third generation (F_1_BC_3_), kernels displaying vitreous, opaque and intermediate phenotypes were observed (Supplementary Fig. 2a, b). With more backcrossing, an equal segregation of vitreous and opaque kernels clearly occurred in F_1_BC_5_ (Supplementary Fig. 2c). We also introgressed the *Ven1*^*D1051*^ and *Ven1*^*P25*^ alleles into A619 and found typical Mendelian segregation appeared in F_1_BC_4_ (Supplementary Fig. 2d, e). These results indicate the penetrance of the *Ven1*^*A619*^ allele is strongly influenced by the genetic background. F_1_BC_7_, seeds with vitreous and opaque phenotypes were propagated by self-pollination for two generations, yielding the homozygous nearly isogenic lines (NILs), NILW64A and NILA619. The NILs were nearly identical in other plant phenotypes but exhibited an apparent difference in kernel texture (Fig. 1a–d). Although NILW64A contained a slightly smaller amount of vitreous endosperm than W64A, it had significantly more than A619 and NILA619 (Fig. 1c, d), thereby creating stronger light transmission on a light box (Fig. 1b).

We pooled the vitreous and opaque seeds segregating in the F_1_BC_5_ population of the W64A by A619 cross (Supplementary Fig. 2f) and performed Bulked Segregant Analysis (BSA) sequencing. This revealed only one candidate region located on chromosome 10 (Fig. 1e). Using 2000 F_1_BC_7_ individuals, the QTL was narrowed down to a 0.2 Mb region (Fig. 1f), where five genes were annotated based on the B73 genome. RNA sequencing (RNA-seq) analysis of 18-DAP (day after pollination) endosperms of W64A, A619, NILW64A and NILA619 revealed only Zm00001d026056 was differentially expressed in W64A and NILW64A, versus A619 and NILA619; the other four genes were expressed at a similar level or expressed at low levels in the endosperm of these lines (Fig. 1g). We amplified and sequenced the gene body and regions within 1.2 kb up- and downstream of the coding region and found the *Ven1*^*A619*^ allele contains a 1250-bp deletion in the 3′ end, resulting in truncation of the *Ven1*^*A619*^ protein (Supplementary Fig. 3). Using a specific primer pair flanking this deletion^10^, we verified that *Ven1*^*A619*^ was also associated with the opaque phenotype in the F_1_BC_4_ populations created by A619 crosses with D1051 and P25 (Supplementary Fig. 4). Consistent with the transcript levels, the VEN1 protein was barely detectable in A619 and NILA619, compared with W64A and NILW64A (Fig. 1h), indicating *Ven1*^*A619*^ is a nonfunctional allele.

To confirm that this mutation is responsible for the opaque endosperm phenotype of A619, we used RNA interference (RNAi) technology to knock down *Ven1* expression in KN5585 (Supplementary Fig. 5a), a vitreous inbred line wildly used in China for maize transformation. Twenty independent T_0_ *Ven1RNAi* plants were recovered and all segregated vitreous and opaque seeds when self-pollinated. The transcript and protein levels of *Ven1* in progeny bearing the *RNAi* transgene were dramatically reduced compared with wildtype kernels, as exemplified in photographs of two events (Fig. 1i, j). A representative ear is shown in Fig. 1k and Supplementary 5b, where three quarters of the seeds inherited the *RNAi* construct and exhibited the opaque endosperm phenotype (Supplementary Fig. 5b). Homozygous *Ven1RNAi* ears showing a fully opaque seed set were obtained after propagating the transgenic seeds (Supplementary Fig. 5c). When NILW64A ears were fertilized by the pollen from heterozygous *Ven1RNAi/+* plants, vitreous and opaque F_1_ seeds segregated at a 1:1 ratio (Fig. 1l, Supplementary Fig. 5d). These genetic data showed that Zm00001d026056 is the gene responsible for the opaque endosperm phenotype in A619.

### *Ven1*^*A619*^ causes over-accumulation of nonpolar carotenoids

**Fig. 2:**
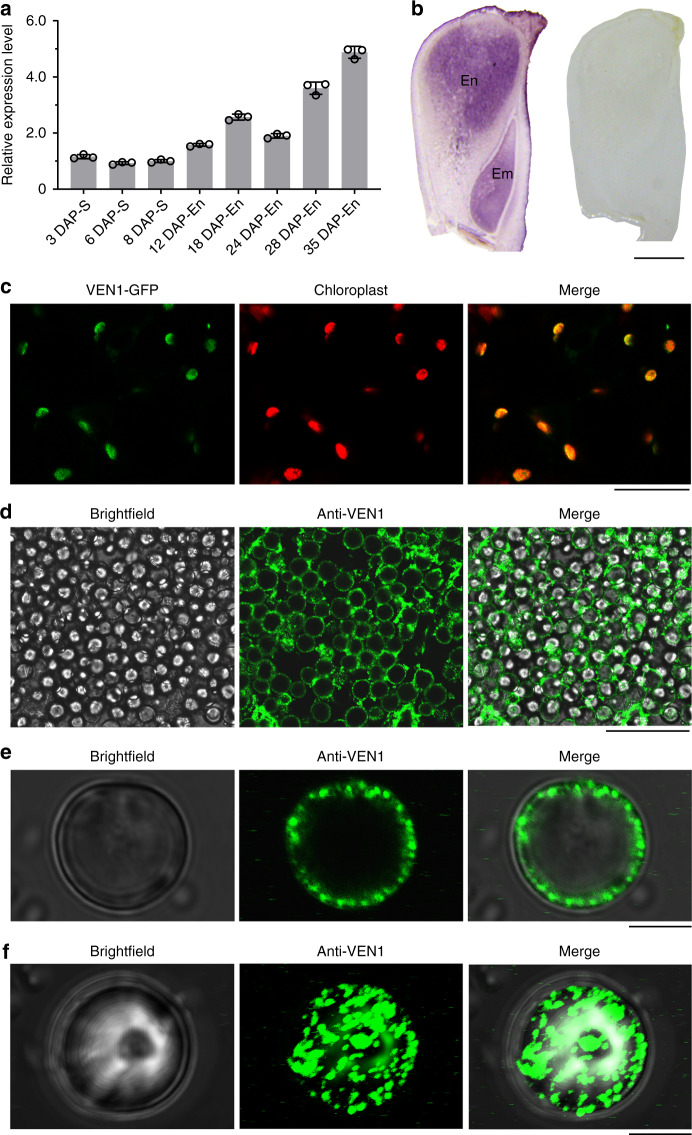
The transcript and protein expression pattern of *Ven1* during seed development. **a** RT-qPCR analysis of *Ven1* expression during seed development. S, Seeds; En, Endosperm. Data are presented as mean values ± SD, *n* = 3 biologically independent samples. **b** RNA in situ hybridization of *Ven1* in the 18-DAP seed. Left panel, hybridization with antisense probes; right panel, hybridization with sense probes. Scale bar, 1 mm **c** Subcellular localization of VEN1-GFP in *Nicotiana benthamiana* chloroplasts. Scale bar, 20 μm. **d** Localization of VEN1 in endosperm starchy cells by immunofluorescence. Scale bar, 50 μm. **e** Ringlike localization of VEN1 on a planar SG. Scale bar, 5 μm. **f** Localization of the VEN1 protein on the surface of a three-dimensional SG by Z-stack imaging. Scale bar, 5 μm. The source data underlying **a** are provided as a Source Data file.

Quantitative RT-PCR revealed *Ven1* is highly expressed in W64A endosperm, where RNA transcript levels gradually increased after 12 DAP, reaching a peak at 35 DAP (Fig. 2a). RNA in-situ hybridization showed *Ven1* was predominantly expressed in starchy endosperm cells from the crown to the kernel center (Fig. 2b). To determine its subcellular localization, the VEN1 protein was fused to GFP and the resulting construct transiently expressed in *Nicotiana benthamiana* leaf cells. The signal from VEN1-GFP was observed to be localized to chloroplasts (Fig. 2c). Specifically, immunohistochemical assays clearly showed the distribution of VEN1 is restricted inside the amyloplast envelope in endosperm cells (Fig. 2d–f).

*Ven1* encodes β-carotene hydroxylase 3 (HYD3), which hydroxylates the β-ionone ring of α-/β-carotene, producing xanthophylls (e.g. lutein and zeaxanthin)^11^. We measured the total carotenoid content in 24- and 35-DAP endosperms and found it was not apparently different between NILW64A and NILA619 (Table 1). However, we found that the nonpolar carotenoids such as phytoene, lycopene, α-carotene and β-carotene, molecules produced upstream of HYD3, accumulated at a significantly higher level in NILA619 than NILW64A, whereas the levels of the polar carotenoids, like lutein and zeaxanthin, downstream of HYD3, were the inverse (Table 1).

**Table 1:** Altered carotenoid composition in endosperm tissues of NILW64A and NILA619.

	Nonpolar carotenoids					Polar carotenoids						Total
	(E/Z)-Phytoene	Lycopene	α-Carotene	β-Carotene	Total	α-Cryptoxanthin	Lutein	β-Cryptoxanthin	Zeaxanthin	Antheraxanthin	Total	
NILW64A-24 DAP	2.38 ± 0.19	0.15 ± 0.02	0.052 ± 0.005	3.17 ± 0.27	5.75 ± 0.42	0.15 ± 0.001	14.13 ± 0.057	1.37 ± 0.12	12.43 ± 0.51	1.20 ± 0.18	29.29 ± 1.30	35.04 ± 1.59
NILA619-24 DAP	6.80 ± 0.47**	0.96 ± 0.20**	0.19 ± 0.08*	6.93 ± 1.02**	14.89 ± 1.41**	0.29 ± 0.03**	11.47 ± 1.65	0.76 ± 0.23*	6.05 ± 1.84**	0.76 ± 0.08*	19.32 ± 3.66*	34.21 ± 4.89
NILW64A-35 DAP	4.03 ± 1.10	0.012 ± 0.004	0.06 ± 0.02	1.69 ± 0.11	5.80 ± 1.18	0.19 ± 0.03	12.93 ± 1.83	1.139 ± 0.13	7.49 ± 0.57	0.69 ± 0.08	22.44 ± 2.51	28.24 ± 2.84
NILA619-35 DAP	9.30 ± 0.27**	0.12 ± 0.02**	0.12 ± 0.002**	4.84 ± 0.24**	14.39 ± 0.23**	0.25 ± 0.02*	10.4 ± 0.35	0.98 ± 0.15	4.15 ± 0.37**	0.46 ± 0.03*	16.25 ± 0.80*	30.64 ± 0.96

* and **, significant differences at *P* < 0.05 and *P* < 0.01 in two-sided Student’s *t*-test, when compared the content of carotenoids in endosperm of NILs at 24 DAP and 35 DAP, respectively. *n* = 3 biologically independent samples; mean and SD were calculated. Source data are provided as a Source Data file.

### *Ven1*^*A619*^ influences SG-PB interactions

To investigate the mechanisms by which *Ven1*^*A619*^ affects vitreous endosperm formation, we first examined zein protein synthesis. We found that A619 accumulates zein proteins at a level that is apparently lower than in W64A (Supplementary Fig. 6a). Therefore, it was possible that insufficient accumulation of zein proteins resulted in a reduction of PB number and size^5^, which in turn caused an opaque phenotype in A619. However, zein RNA transcript and protein levels were not measurably different between NILW64A and NILA619 at all examined stages of grain filling (Supplementary Fig. 6b–e). Since protein content is usually determined by the maternal genotype and endosperm vitreousness by the filial genotype^9,12^, we investigated the correlation of the two traits by pollinating A619 plants with a mixture of W64A and A619 pollen. We found the vitreous and opaque seeds (indicative of the cross with W64A and self-pollination of A619, respectively; Supplementary Fig. 6f) accumulated nearly identical levels of zein proteins (Supplementary Fig. 6g). These results demonstrated that the zein protein level itself is not associated with endosperm texture in this study. We then examined starch synthesis in NILW64A and NILA619 and found the expression of all starch synthetic genes and the total starch and amylose content showed no apparent difference in the two NILs (Supplementary Fig. 7a–d).

Because *Ven1* seems to have no direct effect on the zein proteins and starch synthesis (Supplementary Figs. 6 and 7), we explored how *Ven1*^*A619*^ affects PB and SG development, and subsequently their interaction (Supplementary Fig. 8). At 24 DAP, TEM revealed that SGs and PBs developed normally and showed no apparent difference in size and number between NILW64A and NILA619 (Supplementary Fig. 8a and c). This is consistent with the observation that starch and zein proteins were similarly accumulated in the NILs. When the endosperm cells were examined by SEM, we found that SGs in NILW64A were tightly surrounded by PBs, appearing to be embedded in a proteinaceous cytoskeletal matrix. By contrast, SGs in NILA619 were attached to noticeably fewer PBs and they exhibited an unequal distribution on the SG surface (Supplementary Fig. 8b, d). TEM of 30 DAP kernels revealed PBs in NILW64A endosperm cells increasingly gathered around SGs, whereas in NILA619 PBs were more randomly dispersed among SGs (Supplementary Fig. 8e, g). When observed by SEM, the SGs in NILW64A became embedded in a dense matrix of PBs and cytoplasmic contents. Probably due to tight compacting, the SGs began to form a polygonal shape. In NILA619, the majority of SGs were contacted by a small number of PBs and they maintained their spherical morphology (Supplementary Fig. 8f, h). Thirty five DAP is a critical time point for vitreous endosperm formation. By then most of the starchy endosperm cells have undergone programmed cell death and storage metabolite synthesis is slowing down, water content is rapidly decreasing, and the endosperm is entering the dough stage. In NILW64A, TEM showed that all the PBs converged together, creating a continuous network of matrix grids around the SGs; these grids, with SGs capsuled inside, appeared to function as the basic unit of vitreous endosperm formation. One could imagine that the later-formed vitreous endosperm is composed of numerous interconnecting units like this. In contrast, this grid structure was not organized in NILA619 (Supplementary Fig. 8i, k). In SEM, the SGs in NILW64A were observed to be tightly seamed together, with PBs developing distinctive features of vitreous endosperm formation, whereas the SGs in NILA619 were loosely packed with air space between them, a typical characteristic of opaque endosperm (Supplementary Fig. 8j, l). These results demonstrated that compaction of PBs and SGs is affected during desiccation of NILA619 endosperm.

### *Ven1*^*A619*^ affects the integrity of amyloplast membranes and lipid composition

**Fig. 3:**
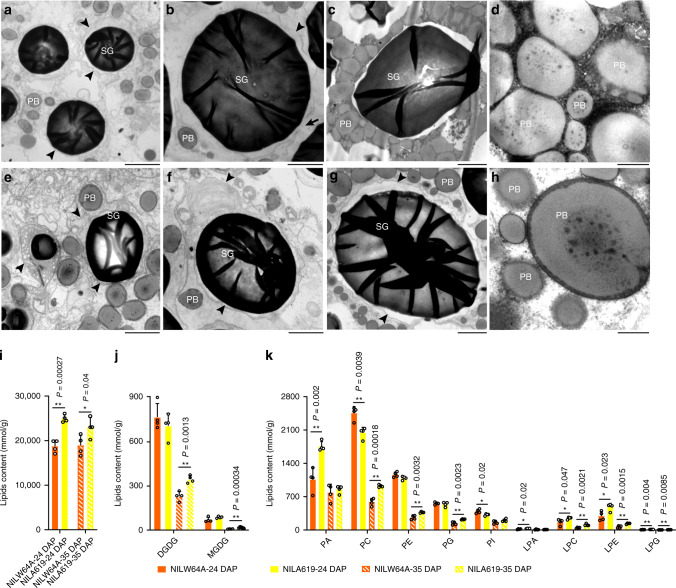
Stability of PB membrane and the changes in lipid composition in endosperm of NILs. **a** NILW64A endosperm cell at 18 DAP. **b** NILW64A endosperm cell at 24 DAP. **c** NILW64A endosperm cell at 35 DAP. **d** Tight packing of PBs and liposome-like structures in NILW64A at 35 DAP. **e** NILA619 endosperm cell at 18 DAP. **f** NILA619 endosperm cell at 24 DAP. **g** NILA619 endosperm cell at 35 DAP. **h**, Loose packing of PBs in NILA619 at 35 DAP. SG, starch grain; PB, protein body. Arrowheads indicate the amyloplast membranes; The arrow indicates broken amyloplast membranes in NILW64A; * indicates liposome-like structures among neighboring PBs in **d** and **h**. Scale bar in **a**–**c**, **e**–**g**, 2 μm; scale bar in **d** and **h**, 500 nm. **i** The total lipid content in endosperms of NILs at 24 and 35 DAP. Data are presented as mean values ± SD, *n* = 4 biological replicates. * and **, significant differences at *P* < 0.05 and *P* < 0.01 in two-sided Student’s *t*-test, respectively. **j**–**k**, The content of galactolipids **j** and phospholipids **k** in endosperms of NILs at 24 and 35 DAP. Data are presented as mean values ± SD, *n* = 4 biological replicates. * and **, significant differences at *P* < 0.05 and *P* < 0.01 in two-sided Student’s *t*-test, respectively. DGDG, digalactosyldiacylglycerols; LPA, lyso-PA; LPC, lyso-PC; LPE, lyso-PE; LPG, lyso-PG; MGDG, monogalactosyldiacylglycerols, PA, phosphatidic acids; PC, phosphatidylcholines; PE, Phosphatidylethanolamines; PG, phosphatidylglycerols; PI, phosphatidylinositols. The source data underlying **i**–**k** are provided as a Source Data file.

To investigate the process of SG and PB interaction during desiccation of NIL-*Ven1*^*A619*^, we examined the organelles by TEM at higher resolution. Amyloplasts are a specialized plastid for starch synthesis and are bounded by two lipoprotein membranes. At 18 DAP, when the SGs in NILW64A were undergoing rapid development, we observed that the amyloplast membranes were intact, indicating they are functional for starch synthesis (Fig. 3a). At 24 DAP, when many SGs in NILW64A were reaching maturity, the amyloplast membranes began to break down, as evidenced by debris in their periphery (Fig. 3b). Because SGs at this stage differed in maturity, those with membranes in integrity or complete degradation (in the latter, the debris was absent in their periphery) were also observed (Supplementary Fig. 9a). At 35 DAP, the amyloplast membranes in NILW64A completely disappeared, enabling PBs to physically interact with SGs (Fig. 3c). It appeared that PBs were pulled together by undefined cytoplasmic components, perhaps by liposome-like structures, through hydrophobic interactions (Fig. 3d). In contrast, the amyloplast membranes in NILA619 behaved differently. At 18 DAP, the membranes appeared to be irregularly expanded, leading to formation of an amorphous structure (Fig. 3e). The membranes in NIL619 appeared to be more persistent; they were intact at 24 DAP (Fig. 3f) and remained intact at 35 DAP (Fig. 3g). It appears the stability of the membranes creates a barrier that prevents PBs and the cytoplasmic matrix from approaching the SGs. There was little of the dense cytoplasmic contents around PBs compared to NILW64A (Fig. 3h). These results suggested *Ven1*^*A619*^ somehow influences the breakdown of amyloplast membranes, which is critical for allowing for SG-PB interaction.

Isolated amyloplast membranes contain about 70% galactolipid and 25% phospholipid (PL), but no neutral lipids like triacylglycerides (TAG) and fatty acids (FA)^13^. Monogalactosyldiacylglycerol (MGDG) and digalactosyldiacylglycerol (DGDG) provide both structural and functional organization of amyloplast. We measured the quantity and composition of lipids in the endosperm at 24 and 35 DAP and found they were markedly different between NILA619 and NILW64A (Fig. 3i–k; Supplementary Fig. 9b–e). In the endosperm, there are mainly neutral lipids, e.g., FA and TAG. In each NIL, total lipid content was not apparently altered at the two time points, but it was always significantly higher in NILA619 than NILW64A (Fig. 3i), suggesting an enhanced capacity for lipid synthesis. At 24 DAP, when amyloplast membranes of many SGs began to break down in NILW64A, only a few polar lipids (PA, LPC and LPE) accumulated at a mildly higher level in NILA619 than NILW64A (Fig. 3j, k). This suggested that although the breakdown of amyloplast membranes occurred in NILW64A, their main lipids, i.e. galactolipids were still present in endosperm cells. The apparent difference between the two NILs were the levels of FA and TAG (Supplementary Fig. 9c). By 35 DAP, when amyloplast membranes of most SGs completely degraded in NILW64A, the level of DGDG, which is a predominant galactolipid in amyloplast membranes, was significantly higher in NILA619 than NILW64A, as well as PL (PC, PE, PG) and lysoPL (LPC, LPE, LPG) (Fig. 3k, Supplementary Fig. 9b). It was evident that the contents of amyloplast lipids, MGDG, DGDG and other membrane phospholipids (PA, PC, PE and PG), were reduced at 35 DAP compared to those at 24 DAP in the two NILs. At this stage, FA and diacylglycerides (DAG, the minor neutral lipid constituent) were significantly higher in NILA619 than NILW64A, although TAG was not apparently different (Supplementary Fig. 9c–e). The difference in lipid composition between NILW64A and NILA619 is consistent with the differential status of amyloplast envelope integrity (Fig. 3b, c, f and g; Supplementary Fig. 9a).

### Epistatic effect of carotenoid synthesis on the opaque phenotype in A619

**Fig. 4:**
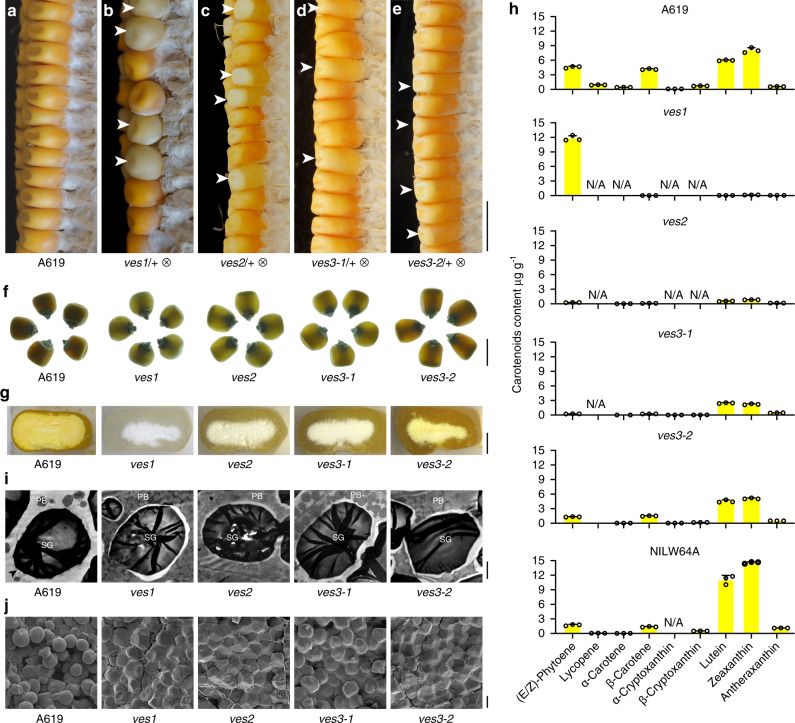
Screening suppressors of *Ven1*^*A619*^. **a** A619 ear; **b**–**e** Ears segregating *ves1* (**b**), *ves2* (**c**), *ves3-1* (**d**) and *ves3-2* (**e**). Vitreous and carotenoid synthesis defective kernels are indicated with white arrowheads. Scale bar, 1 cm. **f** Kernel vitreousness of A619 and the suppressors as observed on the light box. Scale bar, 1 cm. **g** Kernel transverse sections of A619 and the suppressors. Scale bar, 5 mm. **h** Altered carotenoid composition in endosperm tissues of A619, suppressors and NILW64A. Data are presented as mean values ± SD, *n* = 3 biological replicates. **i** TEM of the A619 and suppressors endosperm cells at 30 DAP. SG, starch grain; PB, protein body. Arrowheads indicate the amyloplast membranes. Scale bar, 2 μm. **j** SEM of the A619 and the suppressors endosperm cells at 30 DAP. Scale bar, 10 μm. The source data underlying **h** are provided as a Source Data file.

To further investigate whether over-accumulation of non-polar carotenoids in A619 is associated with the opaque endosperm phenotype, we treated A619 with ethyl methanesulfonate (EMS) and screened for mutants with defects in carotenoid synthesis by observing endosperm color. We identified four single-gene recessive mutants displaying varying differences in endosperm color, suggesting these mutations occurred in different steps of carotenoid synthesis (Fig. 4a–e). A common feature of these mutants was the conversion of the opaque to a vitreous endosperm phenotype. Because these mutations function as suppressors of the *Ven1*^*A619*^ phenotype, we designated them *vitreous endosperm suppressor 1*, *2*, *3-1* and *3-2* (*ves1*, *ves2*, *ves3-1* and *ves3-2*, the last two were subsequently shown to be allelic mutations) (Fig. 4f–g). We measured the carotenoid composition and found the synthesis of lycopene, α-carotene and β-carotene was suppressed in all these mutants (Fig. 4h), confirming a close relationship of the excessive accumulation of non-polar carotenoids and the opaque phenotype in A619 endosperm. We then cloned these genes by BSA sequencing (Supplementary Fig. 10a–d). The carotenoid biosynthesis pathway is conserved in maize and other plants, and the candidate genes, quantitative trait loci, and phenotypic loci are well characterized^11,14–19^. According to these reports, we summarized the maize carotenoid biosynthesis pathway and included the suppressors in Supplementary Fig. 10e. *Ves1* encodes 4-hydroxyphenylpyruvate dioxygenase 1 (HPPD), which is the first committed step in synthesis of both plastoquinone and tocopherols in plants and functionally reverses the *pds1* mutant phenotype in Arabidopsis^20^ (Supplementary Fig. 10e). The enzyme directly downstream of HPPD is homogentisate solanesyl transferase (HST), and its mutation in maize, *w3*, showed blocked carotenoid synthesis by repressing the synthesis of PQ9, the oxidant required for phytoene desaturase (PDS). The *w3* mutant accumulates phytoene and exhibits a white kernel and seeding phenotype. *Ves2* (also known as *seed carotenoid deficient*, *Scd*) encodes a hydroxymethylbutenyl diphosphate synthase (HDS) converting 2C-methyl-D-erytrithol 2,4-cyclodiphosphate to 1-hydroxy-2-methyl-2-(E)-butenyl 4-diphosphate. Mutation of *Scd* inhibits the biosynthesis of MEP-derived isoprenoids (carotenoids, chlorophylls, and tocopherols) and results in a white kennel, albino seedling and eventually lethal phenotype^21^, similar to the *ves2* phenotypes. *Ves3* encodes the 1-deoxy-D-xylulose-5-phosphate synthase (DXS), which is in upstream of VES2, a limiting enzyme for 2-*C*-methyl-D-erythritol 4-phosphate (MEP) for plant plastidic isoprenoid biosynthesis (Supplementary Fig. 10e)^22,23^. Sequencing revealed that *ves1, 2* and *3-1* resulted from a premature stop codon in the second exon (Supplementary Fig. 10a–c) and *ves3-2* was a missense mutation of *DXS*, in which Arg-210 was changed to Trp (Supplementary Fig. 10d). In contrast with the persistent amyloplast membranes in A619, TEM showed that the amyloplast membranes completely degraded in endosperm cells of *ves1*, *2*, *3-1* and *3-2* at 30 DAP, which allowed the PBs to converge around SGs and surround them (Fig. 4i). SEM revealed that the SGs in all suppressor mutant endosperms were tightly compacted with PBs and cytoplasmic contents (Fig. 4j). These results genetically supported that the upstream carotenoid biosynthetic pathway is epistatic to the *Ven1*^*A619*^ phenotype.

### Screening *Ven1*^*A619*^ endosperm modifiers

**Fig. 5:**
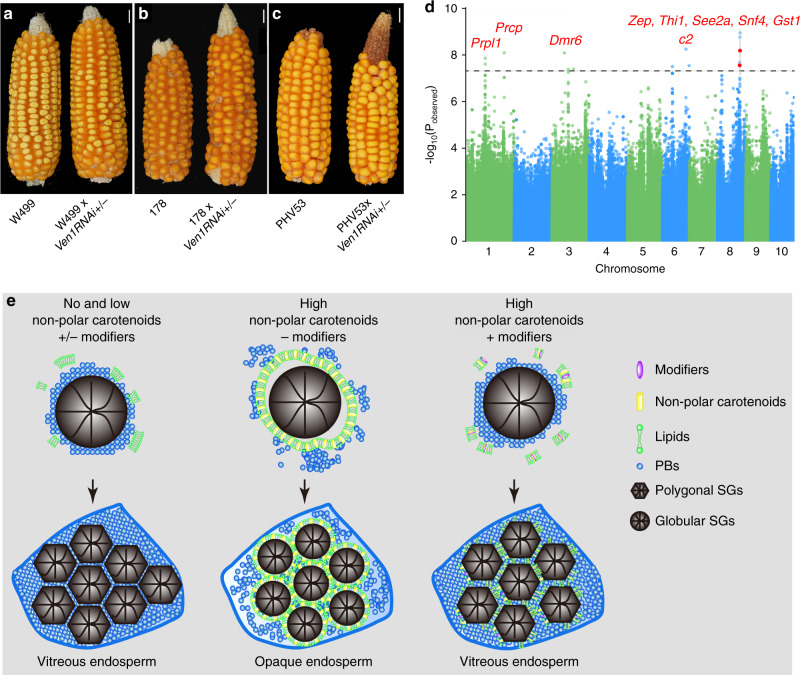
Screening *Ven1*^*A619*^ endosperm modifiers by GWAS and proposed model for vitreous endosperm formation. **a**–**c** Representative ear phenotypes showing full modification (**a**), partial modification (**b**) and totally unmodified (**c**) pollinated by *Ven1RNAi/+*. **d** Manhattan plot of whether the endosperm contains *Ven1*^*A619*^ modifiers. The dashed line indicates the significance threshold of *P*-value 5 × 10^−8^. 27 unique SNPs are labelled with red dots, and the candidate genes are highlighted. The numbers on the horizontal axis indicate the maize chromosomes. **e** Proposed model for vitreous endosperm formation.

During recurrent introgression of *Ven*^*W64A*^ into A619, we did not observe segregation of the opaque phenotype until F_1_BC_3_ (Supplementary Fig. 2a–c). This was different from the cross of NILW64A and *Ven1RNAi/+*, where the F_1_ hybrids of W64A and *Ven1RNAi*/+ were vitreous (Supplementary Fig. 11), suggesting the presence of genetic modifiers in W64A that influence the opaque endosperm phenotype (caused by *Ven1*^*A619*^ or *Ven1RNAi*). We analyzed the *Ven1* genotype in the 262 inbred lines (Supplementary Data 1) and found that 14 bear the *Ven1*^*A619*^ allele, of which 12 are vitreous and only two have an opaque endosperm in the mature kernel (Supplementary Fig. 12a). To identify *Ven1*^*A619*^ modifiers (*Vem*) in the natural population, we screened the 262 lines by crossing them with *Ven1RNAi*/+, no matter whether they are of the *Ven1*^*A619*^ genotype or not. If the inbred lacks modifiers, the F_1_ progeny should segregate vitreous and opaque kernels at a 1:1 ratio; if does, all the progeny are expected to be vitreous. This screen resulted in identification of varying degrees of phenotypic modification in the natural population, wherein 182 were fully modified, 35 partially modified (showing a mosaic phenotype) and 45 totally unmodified (Supplementary Fig. 12a, b; Supplementary Data 1). Examples for each kind of modification are shown in Fig. 5a–c. These data demonstrated that a great proportion of germplasm (30%) in the natural population are not suitable for provitamin A biofortification due to incomplete modification, while *Ven1*^*A619*^ is a rare allele that has potential to increase β-carotene in maize grain.

A genome-wide association study (GWAS) for modifiers identified six obvious peaks (*P* = 5 × 10^−8^) distributed on chromosome 1, 3, 6 and 8 (Fig. 5d). Genetic variation and RNA-seq transcriptome analyses of W64A and A619 endosperms at 18 DAP revealed that nine genes in these regions were highly expressed in W64A, but lowly expressed in A619 (Supplementary Table 1). Among them, glutathione S-transferase 1 catalyzes conjugation of glutathione (GSH; γ-Glu–Cys–Gly) with electrophilic compounds, such as ROS and GSH, becoming an oxidized form, glutathione disulphide (GSSG)^24^. Therefore, higher expression of *Gst1* could alter the redox state in endosperm cells, which may in turn affect the stability of amyloplast membranes and SG-PB interaction during vitreous endosperm formation.

## Discussion

One of the first descriptions of cells in the vitreous endosperm was by Donald Duvick, who likened them to a box of white marbles (SGs) in which buckshot (PBs) was packed between the marbles and viscous cytoplasm functioned as a transparent glue between them, forming a structure like rigid concrete^25^. In this model, mutations affecting synthesis of SGs and zein PBs would affect the formation of vitreous endosperm^9^. Indeed, the starch mutants, *waxy1* and *amylose extender1*, with a low- and high-amylose content in SGs, develop an opaque and nearly fully vitreous endosperm phenotype, respectively. Mutations either reducing zein protein synthesis or altering its deposition in PBs also create an opaque phenotype in the mature endosperm. Besides these mutations that directly affect starch and PB synthesis, there are many others that indirectly affect their synthesis and cause an opaque endosperm phenotype^26–28^. Evidence from these studies support the hypothesis that the number of PBs, the organization of zein proteins within them, and their interaction with SGs are essential factors for vitreous endosperm formation^9^. However, our understanding of the process by which vitreous endosperm forms is incomplete, as other factors can influence it. For example, some mutations, like *opaque1*, have no apparent effect on zein or starch synthesis^29^. Indeed, the chemistry that creates the matrix between PBs and SGs during kernel desiccation is not understood. In this study, we demonstrated that *Ven1*, a QTL affecting the nature of carotenoids in the amyloplast and the stability of the amyloplast membrane, impacts interactions between SGs and PBs. An influence of the amyloplast membrane on vitreous endosperm formation was reported for the *opaque5* mutation^30^. Although the reason is unclear, this mutation affects mono-galactosyldiacylglcerol synthase, the enzyme responsible for synthesis of amyloplast membrane galactolipids. Whether or not this influences amyloplast membrane stability during kernel desiccation was not investigated in detail.

Vitreous endosperm begins to form at the periphery of the kernel by the late dough stage (35–40 DAP). This process could simply be a consequence of condensation of PBs and cellular contents onto SGs. At seed maturity, endomembrane systems would be expected to degrade as a result of PCD and water loss. On the one hand, during vitreous endosperm formation the amyloplast envelope appears to break down before PB membranes (Fig. 3b, c). This would be critical to facilitate PBs and cytoplasmic contents condensing onto the SG surface and creating the matrix grid (Supplementary Fig. 8i, j; Fig. 3c). Our results support the hypothesis that the SG-PB interaction requires degradation of amyloplast membranes, which otherwise would form a barrier to this process (Supplementary Fig. 8k, l; Fig. 3f, g). On the other hand, PBs appear to stay in positions surrounding SGs around the time amyloplast membranes are about to degrade. Timing could be critical to achieve an optimal spatial arrangement of SGs and PBs, otherwise once the starchy endosperm tissue is dehydrated, PBs are unable to approach SGs and condense around them.

While the number and size of PBs matters for vitreous endosperm formation, the assembly of PBs is also a determining factor, as suggested by the *opaque1* and *floury1*^31^ mutations, which are associated with defects in synthesis of myosin or myosin-related proteins, part of the cytoskeleton surrounding the RER^32^. Although the two NILs had identical zein protein abundance (Supplementary Fig. 6b), and thus a similar number and size of PBs (Supplementary Fig. 8a, c), it is evident that PBs don’t gather around SGs (Supplementary Fig. 8c, g, k; Fig. 3e–g). A tentative explanation for the dispersal of PBs in NILA619 is the altered lipid content (Fig. 3i–k and Supplementary Fig. 9). Because zeins are a group of hydrophobic proteins, the association of PBs would involve hydrophobic interactions with lipids in the cytoplasm. In wheat, dark lipid inclusions (containing glycolipids, phospholipids, free fatty acids and monoglycerides) were found to organize in a liquid crystalline phase with protein matrix and SGs during vitreous endosperm formation^33^. The higher level of galactolipids (DGDG and MGDG) in NILA619 endosperms is associated with persistent amyloplast membranes at 35 DAP. In NILW64A, coincident with amyloplast membrane degradation, the membrane lipids significantly decreased (Fig. 3). As a result, the residual membrane lipids might organize the FA and DAG into liposome-like structures. Condensation of liposome-like structures between neighboring PBs is evident in NILW64A, but not NILA619 (Fig. 3c, d, g, h, indicated by asterisks), supporting this hypothesis. Vitreous endosperm formation might be related to the rearrangement of PB membranes and the lipid-mediated aggregation of PBs could be a prerequisite for the adhesion of PBs. As observed in durum wheat^33^, there might be a fusion between PB and amyloplast-derived membranes, especially during the dehydration phase, which will orientate the glass transition of the endosperm matrix towards a vitreous or an opaque solid state. Although maize endosperm contains a low level of lipids, many opaque and floury mutants exhibit defects in the structure of subcellular organelles, e.g., RER, PBs^31^ and amyloplasts^30^, which are bounded by lipoprotein membranes. Alteration in lipid content might affect the PB-PB and SG-PB interactions (Fig. 3i, j), although further molecular and biochemical analyses are required to address this question.

Our data indicate that the composition of carotenoids in amyloplasts influence the stability of amyloplast membranes during kernel desiccation. Although there have been many studies of carotenoid interactions with membranes, little is known about the nature of carotenoids in amyloplast membranes. *Ven1*^*A619*^ is a non-functional allele of *Hyd3*, and results in an increased level of non-polar carotenoids in the kernel (Table 1). Since the VEN1 protein localizes to amyloplast membranes (Fig. 2d–f), there are two possibilities that could explain the abnormalities and persistence of these membranes in mature endosperm of A619. First, VEN1 could function as a structural protein that negatively regulates the membrane stability. This is unlikely, however, because mutations in upstream genes affecting carotenoid synthesis also suppress the opaque kernel phenotype in the presence of *Ven1*^*A619*^ (Fig. 4). Although these mutations affect many other secondary metabolites such as tocopherol biosynthesis, apocarotenoid derivatives and other isoprenoids, they all have the common feature that the levels of lycopene, α-carotene and β-carotene are concurrently reduced, indicating that upstream carotenoid biosynthetic genes are epistatic to the *Ven1*^*A619*^ phenotype and the altered carotenoid composition may trigger an unknown physicochemical or metabolic mechanism to protect amyloplast membranes from breaking down. Second, in contrast to polar carotenoids, e.g., lutein, zeaxamthin, and violaxanthin, that are primarily synthesized in amyloplast membranes in most inbred lines, non-polar carotenoids are in preponderance in NILA619. These carotenoids appear dispensable for the biological functions of amyloplasts, at least in terms of starch synthesis, which is supported by many high yielding commercial white maize varieties containing small amounts of carotenoids. Since the *ves1* endosperm accumulated only one kind of carotenoid, i.e. phytoene, and the level is obviously higher than that in the NILs and other suppressor lines (Fig. 4h), phytoene could be excluded and other nonpolar carotenoids (lycopene, α-carotene or β-carotene) considered most likely responsible for the irregular and persistent amyloplast membranes. Moreover, carotenoids are precursors to hormones and many other apocarotenoids (including many new apocarotenoids recently discovered, such as β-cyclocitral derived from β-carotene, strigolactones, and others)^18,34^. The contents of these apocarotenoids may be changed in the endosperm of NILs and suppressors, which might also influence the stability of amyloplast membranes.

Since carotenoids are located in the membranes of the plastid envelop (here the non-photosynthetic amyloplast), it was logical to hypothesize a connection between the role of carotenoid composition and the physicochemical properties of amyloplast membranes. This hypothesis was strengthened by numerous works showing that, in vitro, carotenoids modulate the physical properties of model lipid membranes. For example, β-carotene tends to be randomly distributed within the hydrophobic interior of the bilayer envelope and increase the membrane fluidity, whereas polar carotenoids span lipid bilayer and have their polar groups anchored in the opposite polar zones of membrane; as a result, they increase the viscosity of the membrane^35–37^. In addition, zeaxanthin, but not β-carotene, increases the rigidity of model phospholipid membranes^35,36^. This effect of polar carotenoids is similar to the effect of cholesterol, i.e., limits the molecular motion of the lipid alkyl chains and favors the extended conformation of the alkyl chains of bilayer membranes. Probably, the elevated nonpolar carotenoids, particularly β-carotene, and decreased polar carotenoids, particularly zeaxanthin, created by *Ven1*^*A619*^ could increase the fluidity and hydrophobicity of amyloplast membranes and result in the irregularly expanded amyloplast membranes (Fig. 3e, f). The altered membrane physical properties could delay breakdown of amyloplast membranes in NILA619 endosperm, although the mechanism is unclear. As a result, NILA619 endosperm cells contain more amyloplast-specific lipids, DGDG, and phospholipids with a higher level of unsaturation than NILW64A (Fig. 3j–k and Supplementary Fig. 9b). Scavenging singlet oxygen (^1^O_2_) in membranes by β-carotene and its polar derivative, zeaxanthin, is effective in membranes containing unsaturated lipids (such as DGDG), but not in membranes containing saturated lipids^38^. One could envision that the higher level of unsaturated DGDG in NILA619 endosperm cells enhances the β-carotene-mediated antioxidant activity to protect the amyloplast membranes from oxidative damage and degradation during kernel dehydration.

*Ven1*^*A619*^, previously identified as *crtRB1* or *Hyd3*, has potential to be applied for high β-carotene breeding in corn^10,11^. Since knock-down of *Ven1* expression causes an undesirable opaque kernel phenotype in many genetic backgrounds, use of the *Ven1*^*A619*^ allele or *Ven1RNAi* could be limited to germplasms with appropriate genetic modifiers (Supplementary Fig. 12). We created an effective screen for genetic modifiers in the natural population that can modify the opaque phenotype caused by *Ven1RNAi* (Fig. 5a). Indeed, several loci were identified, of which *Gst1* encoding Glutathione S-transferase 1 was a major QTL (Fig. 5d), although their mechanisms remain to be understood.

Vitamin A deficiency (VAD) is the leading cause of preventable childhood blindness and is estimated to affect about one-third of the world’s children under the age of five (information from the World Health Organization, https://www.who.int/nutrition/topics/vad/en/). VAD usually results from dietary deficiency, as it mostly occurs in developing countries where the staple food is devoid of β-carotene. One economical approach to prevent VAD is to increase β-carotene levels in cereal grains. Maize, which is one of the most globally grown crops that provides nutritious food for humans, could serve as an ideal crop for provitamin A biofortification^39^. Although the level and composition of total carotenoids in natural maize populations varies dramatically, alleles that increase β-carotene in the grain are rare^10^, suggesting that, for an unknown reason, most selected varieties tend to contain a low β-carotene level. Because carotenoids are more concentrated in the vitreous endosperm than in the starchy endosperm, a better understanding of vitreous endosperm formation is potentially valuable for increasing β-carotene in maize grain.

In any case, maize kernel vitreousness is a complex phenotype and we presented new data that offers insight into how vitreous endosperm forms. Figure 5e shows a model illustrating one possible mechanism for interfering with the vitreous phenotype through carotenoids acting on amyloplast membranes. In white corn varieties or lines with a small amount of non-polar carotenoids, degradation of amyloplast membranes occurs coordinately with interaction between SGs and PBs as endosperm is entering the dough stage, creating the foundation for vitreous endosperm formation. These interactions can be disrupted by increased accumulation of non-polar carotenoids in amyloplast membranes, which stabilizes the membranes during endosperm desiccation by changing the membrane physical properties. In combination, these biochemical changes impair the condensation of PBs and cytoplasm contents onto SGs, leading to the formation of an opaque endosperm phenotype. This phenotype could hinder utilization of superior alleles that increase β-carotene content. Although the nature and mechanisms of genetic modifiers need to be further investigated, their presence in natural populations broadens the germplasm available for breeding high β-carotene varieties of corn that would benefit children suffering from vitamin A deficiencies.

## Methods

### Genetic materials

Yellow dent inbred lines W64A and A619 exhibiting vitreous and opaque endosperm phenotypes, respectively, were used for *Ven1* mapping. By recurrent backcrossing, the *Ven1*^*W64A*^ allele from W64A was introduced into the A619 background for genetic analysis. At F_1_BC_5_, the vitreous and opaque progeny seeds, segregating at a 1:1 ratio, were separated and divided into two pools for BSA sequencing. The vitreous and opaque seeds from F_1_BC_7_ were self-pollinated for two generations, yielding vitreous and opaque NILW64A and NILA619, respectively. Similar experiments were also conducted for two other vitreous inbred lines D1051 and P25 with A619.

Two hundred sixty two temperate maize inbred lines (Supplementary Data 1) provided by Dr. Jinsheng Lai at China Agricultural University, were used for the population genetic analysis.

To make the *Ven1RNAi* construct, reverse and forward *Ven1* cDNA fragments (400 bp in length) were amplified using two primer pairs: *Ven1*-SF2Xba1 and *Ven1*-SR2Sac1, and *Ven1*-AFBspE1 and *Ven1*-ARBamH1. Expression of the *Ven1RNAi* cassette was driven by the *27-kD γ-zein* promoter. The construct was transformed into KN5585, a vitreous inbred line. Transgenic plants were confirmed by PCR using primers described elsewhere^40^.

### Mapping *Ven1*

More than 100 vitreous and opaque kernels were selected for germination from the F_1_BC_5_ ears described above. Then high-quality genomic DNA was extracted individually at the seedling stage for subsequent sequencing. Libraries were constructed using the standard protocol of the Illumina TruSeq DNA PCR-free prep kit. Next-generation sequencing (NGS) technology was used to perform paired-end (PE) sequencing of the libraries based on the Illumina sequencing Nova Seq platform. The sequencing depth was 20x for the parents, and 50x for each mixed pool. Reads of the two bulks and two parents were filtered, quality evaluated, and sequence aligned to the B73 reference genome using bwa software. SNP calling was performed by GATK software and then calculated SNP index. Delta-SNP, the difference between the two bulks, was then determined and the statistical confidence intervals of index were calculated^41^. Sequencing service was provided by Personal Biotechnology Co., Ltd. Shanghai, China.

BSA analysis revealed a candidate interval of approximately 2 Mb. Polymorphic primers were designed (Supplementary Table 2) for fine mapping based on the sequencing data of the two parents. Using 2000 F_1_BC_7_ individuals, the *Ven1* locus was located to an interval between two markers 137.1 M and 137.3 M on chromosome10. These two markers are genetically supported by 6 and 15 recombinant individuals, respectively. Recombinant individuals were selected and then self-pollinated. Further confirmation was by vitreous endosperm phenotypical identification from corresponding recombinants. These data support that *Ven1* was mapped to the target interval segment. We also used these primers to test the F_1_BC_4_ populations created by A619 crosses with D1051 and P25, and found that the *Ven1* markers were linked to the virtuous phenotype.

### Phenotypic analysis

To examine kernel vitreousness, the mature, dry kernels were observed on a light box. To calculate the area of vitreous endosperm relative to the whole endosperm, the kernels were cut transversely and photographed under a light microscope stereoscope (Leica M165 FC). More than 15 seeds of each line were measured by ImageJ software. Then, the percentage of vitreous endosperm was calculated to determine the relative value.

### SEM and TEM observation

For SEM observation, about 2 mm sections in the kernel’s peripheral regions at 24, 30 and 35 DAP were removed and immediately frozen in liquid nitrogen, and then dried using a freeze-vacuum dryer CoolSafe 55-4PRO (ScanLaf). Sections from mature seeds were directly processed for SEM observation.

For TEM observation, about 1 mm sections in the kernel peripheral regions at 24, 30 and 35 DAP were removed and immediately fixed in 2.5% glutaraldehyde in phosphate buffer pH 7.2, then dehydrated, and embedded following the standard protocol^40^. Ultrathin sections of the samples were cut with a diamond knife on a Leica EMUC6-FC6 ultramicrotome and imaged at 80 kV with a Hitachi H-7650 transmission electron microscope.

### Measurement of the proteins and starch

Twenty kernels selected from the middle of each ear had their embryo, seed coat and aleurone removed. The resulting endosperms were dried in an oven (42 °C), and then ground into fine powder with a prototype machine (60HZ, 60S). The powder was filtered through an 80-mesh sieve to prepare samples for starch measurement.

For analysis of zeins in immature kernels, endosperms were collected at 18 and 24 DAP, and ground in the lipid nitrogen into fine powder. The extraction and analysis of zein proteins was according to the standard method of our laboratory^42^. For assaying the total protein content, 60 mg of endosperm powder was used for measurement by the instrument of ELEMENTAR Rapid N exceed. The starch content of endosperm was measured with a Total Starch Assay Kit (K-TSTA; Megazyme) following the standard protocol^43^. Amylose was quantified according to the Megazyme amylose/amylopectin assay procedure (K-AMYL; Megazyme). Six biological replicates were performed.

### RNA-seq and RT-qPCR analysis

Developing endosperms were ground in liquid nitrogen. Total RNA was extracted by TRIzol reagent (Invitrogen, catalog number 15,596,018) and then purified with an RNeasy Mini Kit (Qiagen, catalog number 74,106) after DNase 1 digestion (Qiagen, catalog number 79,254) following the manufacturer’s protocol.

RNA-seq libraries of W64A, A619, NILW64A and NILA619 were prepared with an Illumina Standard library preparation kit and sequenced by HiSeqTM 2500 at OE Biotech Co, Ltd (Shanghai, China). Quality control checks on raw sequencing data were performed by FastQC combined with the NGS QC TOOLKIT v2.3.3. Clean reads were aligned to the B73 reference genome (RefGen_v3) and the reference gene model dataset (FGS 5b) using TopHat/Bowtie2N (ccb.jhu.edu/software/tophat/). The gene expression value was calculated as fragments per kilobase of transcript per million mapped reads (FPKM). The FPKM value should be greater than zero for an expressed gene in the three biological replicates. Candidate genes and genes involved in synthesis of zeins and starch were selected and analyzed, respectively. The related web links are as follow: reference genome [ftp://ftp.ensemblgenomes.org/pub/plants/current/fasta/zea_mays/dna/], reference transcript [ftp://ftp.ensemblgenomes.org/pub/plants/current/fasta/zea_mays/cdna/], and annotations [ftp://ftp.ensemblgenomes.org/pub/plants/current/fasta/zea_mays/cdna/].

For RT-qPCR analysis of *Ven1* expression during seed development and filling, the digested RNA was used for reverse transcription with Super Script III First Strand Kit (Invitrogen). The resulting cDNA was diluted to 50 ng/μL for RT-qPCR with SYBR Green (TAKARA) on a CFX Connect Real-Time System (Bio Rad). The relative gene expression level was calculated using the comparative CT method (_ΔΔ_Ct method), and the maize *Actin* gene was used as a control. The expression level in seeds at 8 DAP was set to 1. In addition, expression of genes involved in zein and starch synthesis were also analyzed using 24 DAP and 30 DAP endosperms. Primers were listed in Supplementary Table 2.

### Amplification of *Ven1* in NILW64A and NILA619

Genomic DNA of NILW64A and NILA619 were extracted from seedling leaves using the CTAB method and used to amplify of the full-length genomic sequence of *Ven1* using primers *Ven1*-GF and *Ven1*-GR. The cDNAs reverse-transcribed from mRNAs of NILW64A and NILA619 endosperms were used for amplification of the *Ven1* coding sequence (CDS) with the primers *Ven1*-CF+*Ven1*W64A-CR, and *Ven1*-CF+*Ven1*A619-CR, respectively. The PCR products were then cloned into the pEASY Vector using pEASY-Blunt Zero Cloning Kit (TransGen, China), and six clones for each fragment were sequenced (TSINGKE Co., Ltd., China).

### RNA in situ hybridization

18-DAP seeds from the middle region of the ears were harvested and fixed in 4% paraformaldehyde solution with 0.1% Triton X-100 and 0.1% Tween 20 in phosphate-buffered saline (PBS) (Takara, Cat# T900) overnight. The seeds were dehydrated using a gradient concentration of ethanol and then were embedded in paraffin. A 400-bp cDNA fragment of *Ven1* was amplified using the primer pair of *Ven1*-probeSac1 and *Ven1*-probeBamH1. The fragment was cloned into the pEASY vector for the synthesis of antisense and sense RNA probes according to the instructions for DIG RNA Labeling Mixture (Roche Cat# 11175025910). 10-μm seed sections were cut and then hybridized with the RNA probes at 50 °C overnight. After blotting with Anti-digoxigenin AP-conjungate (Roche Cat# 11093274910) and incubation with the NBT solution (Roche Cat# 11383213001), the sections were observed and photographed with an ECLIPSE 80i microscope.

### Antibody preparation

A fragment of the partial VEN1 protein from the 223^rd^ to 319^th^ amino acid, was used to produce antibodies by ABclonal, Wuhan, China. After affinity purification, the antibodies were used for Western blotting and immunofluorescence histochemistry.

### Immunoblotting analysis

The nonzein protein extraction was according to the standard method of our laboratory^42^. Twenty μg of nonzein proteins were used for immunoblotting analysis following the manufacture’s protocol, (Bio-Rad, catalog number 162-0177). VEN1 and the control protein ACTIN antibodies were diluted 1:1000, while the secondary antibodies, goat anti-rabbit IgG-horseradish peroxidase (HRP) (Abmart, catalog number M21002L) and goat anti-mouse IgG-HRP (Abmart, catalog number M21001L), were diluted 1:5000 for performing the immune reaction. The membrane was treated with HRP chemiluminescent substrate reagent (Invitrogen, catalog number WP20005) and imaged using a Tanon-5200 system (Tanon).

### Fluorescence immunohistochemistry analysis

Fluorescence immunohistochemistry was used to detect the location of VEN1 in endosperm cells. Briefly, 18-DAP NILW64A endosperms were fixed, embedded and cut as described above for RNA in situ hybridization. 10-μm sections were blocked with 5% bovine serum albumin (BSA) (Yeasen Cat# 36103ES60) in PBS for 1 h and then incubated with anti-VEN1 antibodies (1:100) overnight in the dark at 4 °C. After washing three times with PBS, the samples were incubated with the secondary antibody, Alexa Fluor 488-conjugated goat anti-rabbit lgG (Yeasen Cat# 33106ES60). The sections were observed with an LSM880 confocal microscope under Airyscan modes (Zeiss, Jena, Germany). Z-stack images of SGs were taken in the Airyscan superresolution mode.

### Subcellular localization of VEN1 in Nicotiana benthamiana leaf cells

The full-length *Ven1* coding sequence was amplified using primers *Ven1*-KF and *Ven1*-BR and inserted into a pCAMBIA1301 plasmid driven by the 35 S promoter. The construct was transferred into *Agrobacterium tumefaciens* (GV3101 strain) and injected into 3-week-old *Nicotiana benthamiana* leaves. After two days infiltration, the transfected leaves were observed suing an LSM880 confocal microscope under Airyscan modes (Zeiss, Jena, Germany).

### Determination of carotenoid composition

Carotenoid composition was measured by MetWare (Wuhan, China). To measure carotenoid composition, endosperms from three ears of NILW64A and NILA619 were collected and kept at −80 °C until use. The endosperms were homogenized and ground into fine powder, then dried using a freeze-vacuum dryer. Fifty mg of endosperm powder was extracted with mixture of n-hexane: acetone: ethanol (2:1:1, V/V/V) and an internal standard was added. After two extractions, the supernatant was evaporated to dryness under nitrogen, and reconstituted in a solution of methanol: MTBE. The solution was passed through a 0.22 μm filter and then analyzed with a LC-APCI-MS/MS system (UHPLC, ExionLC™ AD, https://sciex.com.cn/; MS, Applied Biosystems 6500 Triple Quadrupole, https://sciex.com.cn/). A YMC C30 (3 µm, 2 mm × 100 mm) column was used for HPLC analysis. Samples were eluted using a gradient from solvent A, methanol: acetonitrile (3:1, V/V) added 0.01% BHT and 0.1% formic acid), to solvent B, methyl tert-butyl ether (0.01% BHT). The analysis was carried out at 28 °C with a flow rate of 0.8 mL/min. MS analysis was performed using the API 6500 Q TRAP LC/MS/MS System, equipped with an APCI Turbo Ion-Spray interface, operating in a positive ion mode and controlled by Analyst 1.6.3 software (AB Sciex). Carotenoid contents were detected by MetWare (http://www.metware.cn/) based on the AB Sciex QTRAP6500 LC-MS/MS platform.

### Determination of the lipid composition

Lipid composition was measured by MetWare (Wuhan, China) following the standard protocol^44^. For lipidomics analysis, 220 mg dried endosperm powder was extracted with 3 mL of 2-propanol for 1 h and centrifuged (3000 g, 5 min). The pellet was extracted three times in dichloromethane: methanol (2:1, V/V) for 1 h. The supernatant was pooled and dried on a rotary evaporator. Lipids were solubilized in a mixed solution of dichloromethane: methanol, to which 2 mL of 0.9% NaCl in water was added. The lower organic phase was collected and dried under nitrogen flux. Then, lipids were solubilized in 2-propanol before UPLC analysis. Lipids were analyzed on an Ultra High Performance Liquid Chromatography System (UPLC lc-30a) equipped with a Phenomenex Kinetex C18 column (100 × 2.1 mm, 2.6 µm) column. Lipids were eluted using a gradient from solvent A, H_2_O: MeOH: CAN (1:1:1 containing 5 mM NH4Ac), to solvent B, IPA: ACN (5:1 containing 5 mM NH_4_Ac). The analysis was carried out at 60 °C with a flow rate of 0.4 mL/min. MS analysis was performed using the AB Sciex TripleTOF® 6600 System, operating in a positive ion mode and controlled by Analyst 1.6.3 software (AB Sciex). Glycolipids were quantified using PE as the standard.

### EMS mutagenesis and cloning of suppressors

A619 pollen was collected in a 50 mL tube, added to EMS reagent diluted in mineral oil (1:1000, vol/vol), and incubated for 45 min. The mutagenized pollen was applied to the A619 ears. The resulting seeds were planted and surviving plants self-pollinated, yielding more than 2000 ears. Among them, four segregated kernels that were one quarter vitreous and defective in carotenoid synthesis. The four mutants were cloned by BSA sequencing. The BSA sequencing and analyses were provided by OE biotech Co., Ltd. (Shanghai, China).

### Screening genetic modifiers of *Ven1RNAi* in the natural population

Two hundred sixty two inbred lines were pollinated by *Ven1RNAi*/+. The F_1_ endosperm phenotypes were divided into 3 levels: vitreous (completely modified), mosaic (partially modified) and opaque (completely unmodified). According to the phenotype, GAWS analysis was performed using the vitreous and opaque groups to screen the modifiers in the natural population following the standard protocol^45^.

### Statistics and reproducibility

Three independent replications were performed to observe the vitreous endosperm formation in W64A, A616, NILW64A and NILA619 by SEM shown in Fig. 1c and at least six kernels were analyzed in each experiment. Western blotting experiments in Fig. 1h and j were repeated three times. Confocal analyses of the HYD3 localization in leaf and endosperm cells were performed three independent times in Fig. 2c–f. TEM and SEM analyses in Figs. 3, 4 and Supplementary Fig. 8 were performed with 20 kernels from three cobs in each experiment. At least 100 endosperm cells were observed to investigate the changes in starch granule membranes shown in Figs. 3a–h, 4i and Supplementary Fig. 9a. 182 and 200 individual plants were used to perform genetic linkage analysis by PCR in two BC4F1 populations, respectively, shown in Supplementary Fig. 4b and d. Three independent replications were performed to analyze the zein protein content shown in Supplementary Fig. 6a, b, g.

### Reporting summary

Further information on research design is available in the Nature Research Reporting Summary linked to this article.

## Supplementary information

Supplementary Information

Peer Review File

Reporting Summary

Description of Additional Supplementary Files

Supplementary Data 1

## Data Availability

Data supporting the findings of this work are available within the paper and its Source Data. A reporting summary for this Article is available as a Supplementary Information file. The genetic materials generated and analyzed during the current study are available from the corresponding author upon request. Source data are provided with this paper.

## Source data

Source Data

## References

[1] Gibbon BC, Larkins BA (2005). Molecular genetic approaches to developing quality protein maize. Trends Genet..

[2] Vasal S. K., Villegas E., Bjarnason M., Gelaw B., Goertz P. Genetic modifiers and breeding strategies in developing hard endosperm *opaque-2* materials. In *Improvement of Quality Traits of Maize for Grain and Silage Use* (eds Pollmer, W. G. & Phillips, R. H.). Martinus Nijhoff (1980).

[3] Caballero-Rothar NN, Abdala LJ, Borras L, Gerde JA (2019). Role of yield genetic progress on the biochemical determinants of maize kernel hardness. J. Cereal Sci..

[4] Gayral M (2016). Transition from vitreous to floury endosperm in maize (Zea mays L.) kernels is related to protein and starch gradients. J. Cereal Sci..

[5] Fox G, Manley M (2009). Hardness methods for testing maize kernels. J. Agric. Food Chem..

[6] Larkins BA, Hurkman WJ (1978). Synthesis and deposition of zein in protein bodies of maize endosperm. Plant Physiol..

[7] Lending CR, Larkins BA (1989). Changes in the zein composition of protein bodies during maize endosperm development. Plant cell.

[8] Wu, Y., Messing, J. Understanding and improving protein traits in maize. In *Achieving sustainable cultivation of maize* Vol 1: *From improved varieties to local applications* (eds Watson, D.) (Burleigh Dodds Science Publishing, 2017).

[9] Larkins B. A., Wu Y., Song R., Messing J. Maize Seed Storage Proteins. In: *Maize Kernel Development* (ed. Larkins, B. A.) (CABI, 2017).

[10] Yan J (2010). Rare genetic variation at Zea mays crtRB1 increases beta-carotene in maize grain. Nat. Genet..

[11] Vallabhaneni R (2009). Metabolite sorting of a germplasm collection reveals the hydroxylase3 locus as a new target for maize provitamin A biofortification. Plant Physiol..

[12] Wu Y, Messing J (2012). RNA interference can rebalance the nitrogen sink of maize seeds without losing hard endosperm. PLoS ONE.

[13] Gardner HW, Miernyk JA, Christianson DD, Khoo U (1987). Isolation and characterization of an amyloplast envelope-enriched fraction from immature maize endosperm. Physiol. Plant.

[14] Li F, Murillo C, Wurtzel ET (2007). Maize Y9 encodes a product essential for 15-cis-zeta-carotene isomerization. Plant Physiol..

[15] Chen Y, Li F, Wurtzel ET (2010). Isolation and characterization of the Z-ISO gene encoding a missing component of carotenoid biosynthesis in plants. Plant Physiol..

[16] Quinlan RF (2012). Synergistic interactions between carotene ring hydroxylases drive lutein formation in plant carotenoid biosynthesis. Plant Physiol..

[17] Wurtzel ET, Cuttriss A, Vallabhaneni R (2012). Maize provitamin a carotenoids, current resources, and future metabolic engineering challenges. Front. Plant Sci..

[18] Wurtzel ET (2019). Changing form and function through carotenoids and synthetic biology. Plant Physiol..

[19] Beltran J (2015). Control of carotenoid biosynthesis through a heme-based cis-trans isomerase. Nat. Chem. Biol..

[20] Norris SR, Shen X, DellaPenna D (1998). Complementation of the Arabidopsis pds1 mutation with the gene encoding p-hydroxyphenylpyruvate dioxygenase. Plant Physiol..

[21] Zhang L (2019). Seed carotenoid deficient functions in isoprenoid biosynthesis via the plastid MEP pathway. Plant Physiol..

[22] Estevez JM, Cantero A, Reindl A, Reichler S, Leon P (2001). 1-Deoxy-D-xylulose-5-phosphate synthase, a limiting enzyme for plastidic isoprenoid biosynthesis in plants. J. Biol. Chem..

[23] Vallabhaneni R, Wurtzel ET (2009). Timing and biosynthetic potential for carotenoid accumulation in genetically diverse germplasm of maize. Plant Physiol..

[24] Labrou NE, Papageorgiou AC, Pavli O, Flemetakis E (2015). Plant GSTome: structure and functional role in xenome network and plant stress response. Curr. Opin. Biotechnol..

[25] Duvick DN (1961). Protein granules of maize endosperm cells. Cereal Chem..

[26] Miclaus M, Wu Y, Xu JH, Dooner HK, Messing J (2011). The maize high-lysine mutant opaque7 is defective in an acyl-CoA synthetase-like protein. Genetics.

[27] Wang G (2011). Opaque7 encodes an acyl-activating enzyme-like protein that affects storage protein synthesis in maize endosperm. Genetics.

[28] Yang J, Fu M, Ji C, Huang Y, Wu Y (2018). Maize oxalyl-CoA decarboxylase1 degrades oxalate and affects the seed metabolome and nutritional quality. Plant Cell.

[29] Wang G (2012). Opaque1 encodes a myosin XI motor protein that is required for endoplasmic reticulum motility and protein body formation in maize endosperm. Plant Cell.

[30] Myers AM (2011). Maize opaque5 encodes monogalactosyldiacylglycerol synthase and specifically affects galactolipids necessary for amyloplast and chloroplast function. Plant Cell.

[31] Holding DR (2007). The maize floury1 gene encodes a novel endoplasmic reticulum protein involved in zein protein body formation. Plant Cell.

[32] Clore AM, Dannenhoffer JM, Larkins BA (1996). EF-1[alpha] is associated with a cytoskeletal network surrounding protein bodies in maize endosperm cells. Plant Cell.

[33] Saleh AA, Marion D, Gallant DJ (1986). Microstructure of mealy and vitreous wheat endosperms (Triticum durum L.) with special emphasis on location and polymorphic behaviour of lipids. Food Struct..

[34] Dickinson AJ (2019). beta-Cyclocitral is a conserved root growth regulator. Proc. Natl Acad. Sci. USA.

[35] Gabrielska J, Gruszecki WI (1996). Zeaxanthin (dihydroxy-beta-carotene) but not beta-carotene rigidifies lipid membranes: a 1H-NMR study of carotenoid-egg phosphatidylcholine liposomes. Biochim. Biophys. Acta.

[36] Gruszecki WI, Strzałka K (2005). Carotenoids as modulators of lipid membrane physical properties. Biochim. Biophys. Acta.

[37] Popova A. V., Andreeva A. S. Chapter Eight - carotenoid–lipid interactions. In: *Advances in Planar Lipid Bilayers and Liposomes* (eds Iglič, A. & Genova, J.) (Academic Press, 2013).

[38] Widomska J, Welc R, Gruszecki WI (2019). The effect of carotenoids on the concentration of singlet oxygen in lipid membranes. Biochim. Biophys. Acta.

[39] Harjes CE (2008). Natural genetic variation in lycopene epsilon cyclase tapped for maize biofortification. Science.

[40] Wu Y, Messing J (2010). RNA interference-mediated change in protein body morphology and seed opacity through loss of different zein proteins. Plant Physiol..

[41] Li C (2020). Long-read sequencing reveals genomic structural variations that underlie creation of quality protein maize. Nat. Commun..

[42] Zheng X (2019). Intra-kernel reallocation of proteins in maize depends on VP1-mediated scutellum development and nutrient assimilation. Plant Cell.

[43] Deng Y, Wang J, Zhang Z, Wu Y (2020). Transactivation of Sus1 and Sus2 by Opaque2 is an essential supplement to sucrose synthase-mediated endosperm filling in maize. Plant Biotechnol. J.

[44] Xie Y (2020). Ultrasound-assisted one-phase solvent extraction coupled with liquid chromatography-quadrupole time-of-flight mass spectrometry for efficient profiling of egg yolk lipids. Food Chem..

[45] Liu H (2016). Gene duplication confers enhanced expression of 27-kDa gamma-zein for endosperm modification in quality protein maize. Proc. Natl Acad. Sci. USA.

