# Supplementary material for: Carotenoids modulate kernel texture in maize by influencing amyloplast envelope integrity
Source: Nat Commun. 2020 Oct 22;11:5346. doi: 10.1038/s41467-020-19196-9 (PMC7582188; doi:10.1038/s41467-020-19196-9)
Supplement: Supplementary file 1 — Supplementary Information [file 41467_2020_19196_MOESM1_ESM.pdf]

# Carotenoids modulate kernel texture in maize by influencing amyloplast envelope integrity

Wang *et al.*

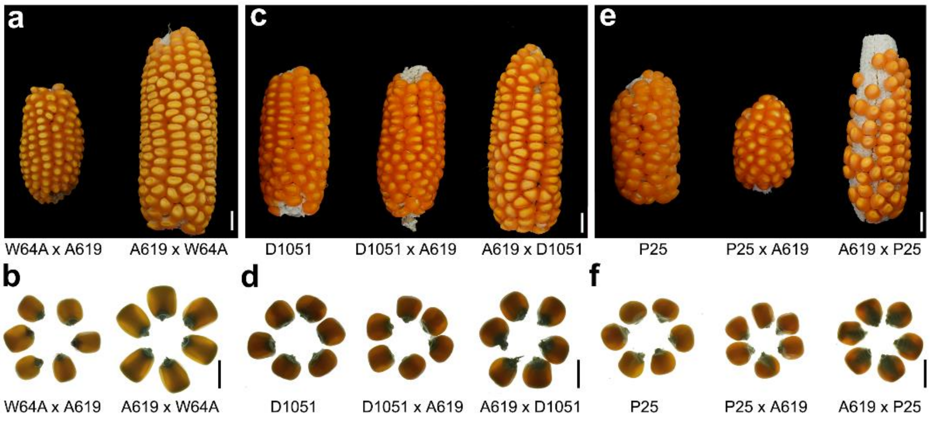


**Supplementary Figure 1. Ear and kernel phenotypes of reciprocal crosses of three vitreous inbred lines and the opaque A619.**

**a**-**b,** Ear (**a**) and kernel (**b**) phenotypes of reciprocal crosses of W64A and A619. **c**-**d,** Ear (**c**) and kernel (**d**) phenotypes of reciprocal crosses of D1051 and A619. **e**-**f,** Ear (**e**) and kernel (**f**) phenotypes of reciprocal crosses of P25 and A619. Scale bar, 1 cm.

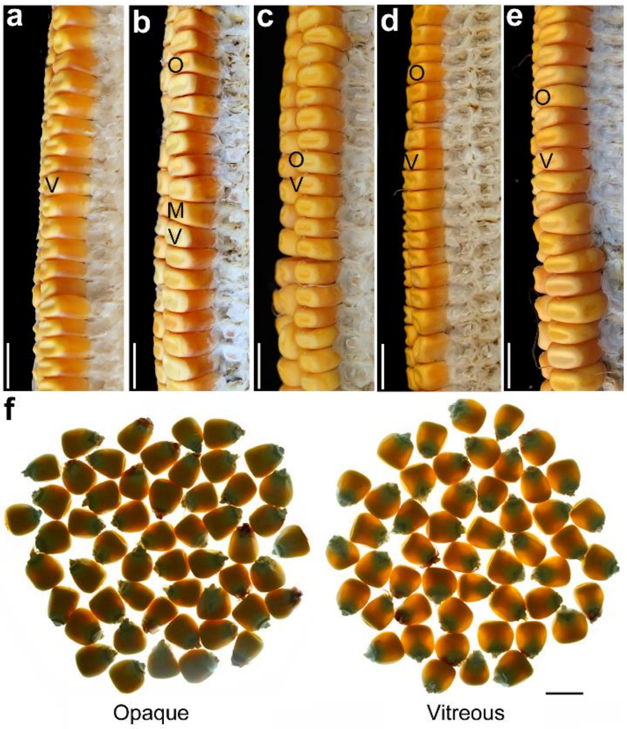


**Supplementary Figure 2. Introgression of *Ven1* alleles from W64A, D1051 and P25 into the A619 background.**

**a,** The $F_1BC_1$ ear phenotype of W64A and A619. **b,** The $F_1BC_3$ ear phenotype of W64A and A619. **c,** The $F_1BC_5$ ear phenotype of W64A and A619. **d,** The $F_1BC_4$ ear phenotype of D1051 and A619. **e,** The $F_1BC_4$ ear phenotype of P25 and A619. V, vitreous kernels; M, kernels showing mosaic phenotype; O, opaque kernels. Scale bar, 1 cm. **f,** Vitreous and opaque kernels segregating from the $F_1BC_5$ population created by W64A and A619. Scale bar, 1 cm.

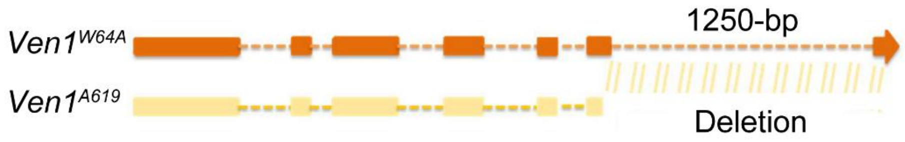


**Supplementary Figure 3. Gene structures of *Ven1*$^{W64A}$ and *Ven1*$^{A619}$.** *Ven1*$^{A619}$ contains a 1250-bp deletion at the 3' terminal compared with *Ven1*$^{W64A}$.

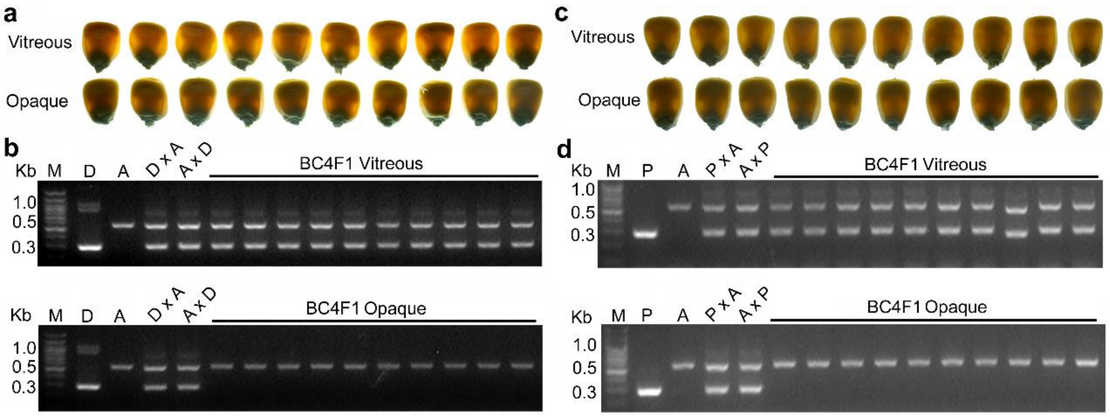


**Supplementary Figure 4. Linkage analysis of *Ven1* and kernel vitreousness in $F_1BC_4$ created by backcrossing of D1051 and P25 with A619.**

**a,** Segregation of vitreous and opaque kernels in $F_1BC_4$ created by backcrossing of D1051 with A619. **b,** The vitreous phenotype is linked with *Ven1*$^{D1051}$. **c,** Segregation of vitreous and opaque kernels in $F_1BC_4$ created by backcrossing of P25 with A619. **d,** The vitreous phenotype is linked with *Ven1*$^{P25}$. Scale bar, 1 cm. The source data underlying Supplementary Figure 4b and 4d are provided as a Source Data file.

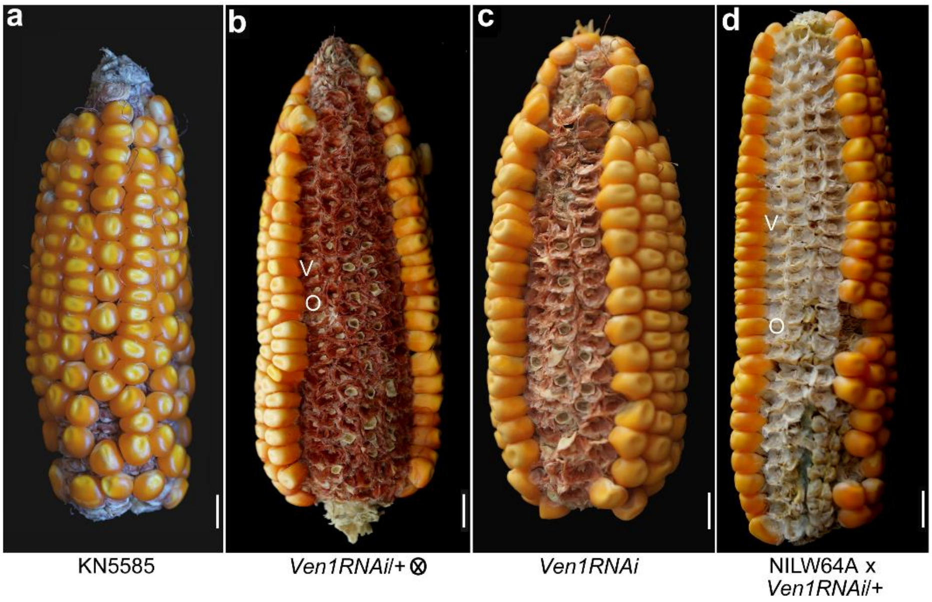


**Supplementary Figure 5. Genetic validation of the *Ven1* function by RNAi.**

**a,** Ear phenotype of the transgenic recipient KN5585. **b,** Ear phenotype of a self-pollinated *Ven1RNAi/+* ear segregating vitreous and opaque seeds. **c,** Ear phenotype of a homozygous *Ven1RNAi* showing all opaque kernels. **d,** A representative ear from a cross of NILW64A x *Ven1RNAi/+* pollen segregating vitreous and opaque kernels at a 1:1 ratio. V, vitreous kernels; O, opaque kernels. Scale bar, 1 cm.

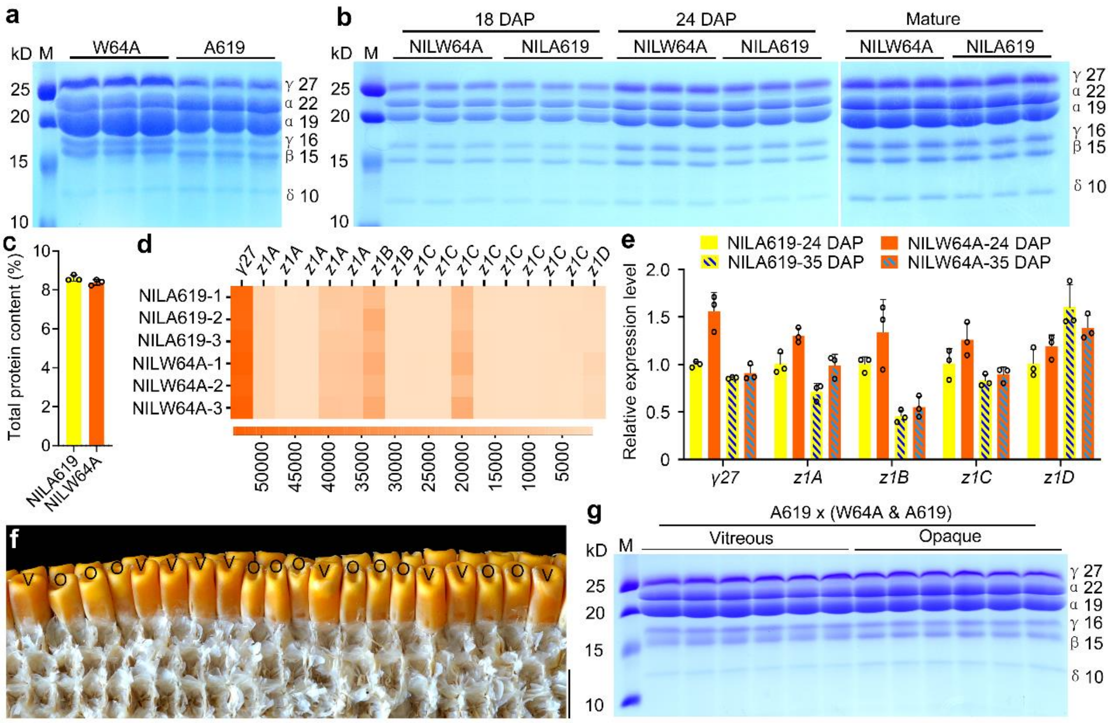


**Supplementary Figure 6. Analysis of zein proteins in endosperms of NILs.**

**a,** SDS-PAGE analysis of zein proteins in W64A and A619 mature seeds. The size of each zein protein band is indicated beside it. **b,** SDS-PAGE analysis of zein proteins in NILW64A and NILA619 at 18, 24 DAP and mature seeds. **c,** Total protein content in NILA619 and NILW64A endosperms. Data are presented as mean values ± SD, n=3 biological replicates. **d,** RNA-seq data of *zein* genes in 18-DAP endosperms of NILW64A and NILA619. **e,** RT-qPCR analysis of *zein* gene expression in NILW64A and NILA619 endosperms at 24 DAP and 35 DAP. The values are shown as the means ± SD. of three biological replicates. **f,** The phenotype of an ear from A619 plant pollinated by equally mixed pollen of A619 and W64A. The genotypes of the vitreous and opaque kernels are heterozygous $Ven1^{W64A}/Ven1^{A619}$ and homozygous $Ven1^{A619}/Ven1^{A619}$, respectively. V, vitreous kernels; O, opaque kernels. Scale bar, 1 cm. **g**, SDS-PAGE analysis of zein proteins from vitreous and opaque kernels from **f**. The source data underlying Supplementary Figure 6b-e are provided as a Source Data file.

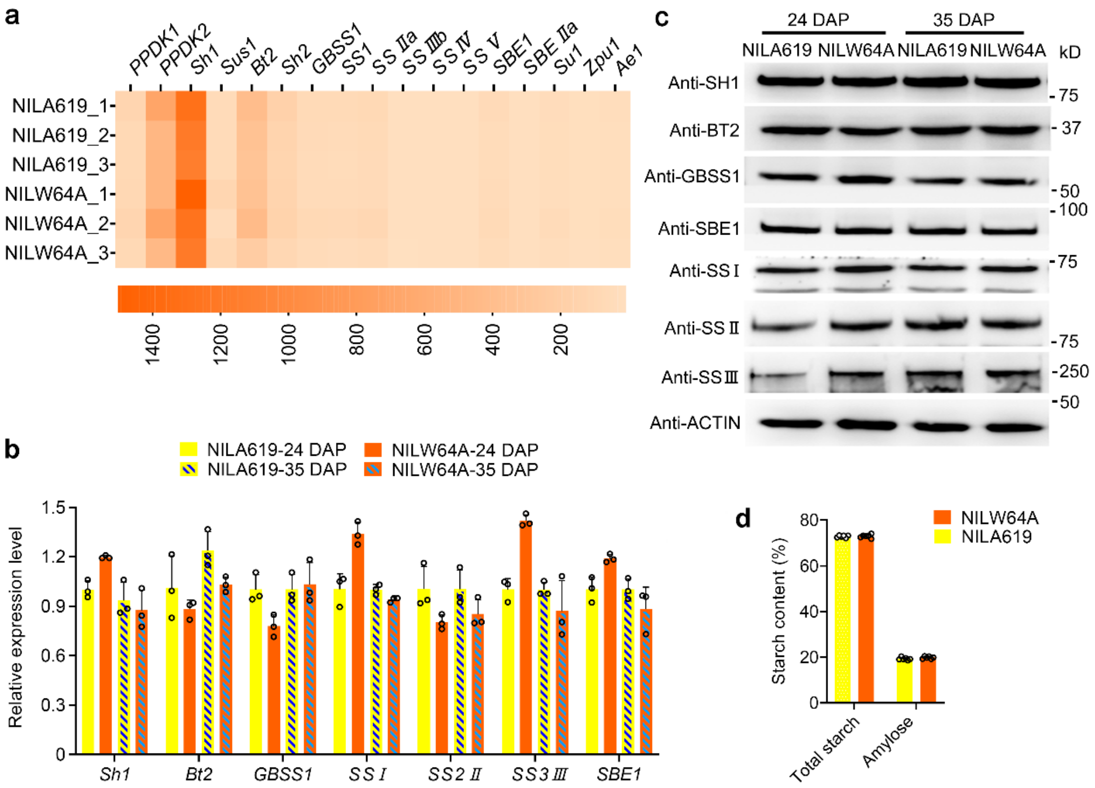


**Supplementary Figure 7. Analysis of the starch synthesis in endosperms of NILs.**

**a,** RNA-seq data of primary starch synthetic genes in 18-DAP endoderms of NILW64A and NILA619. **b,** RT-qPCR analysis of primary starch synthetic genes in NILW64A and NILA619 endosperms at 24 DAP and 35 DAP. The values are shown as the means ± SD of three biological replicates. **c,** Immunoblotting analysis of starch synthetic proteins in NILW64A and NILA619 endosperms at 24 DAP and 35 DAP. ACTIN was used as an internal control. **d,** Starch content in mature endosperms of NILW64A and NILA619. Data are presented as mean values ± SD, n=6 biological replicates. Source data are provided as a Source Data file.

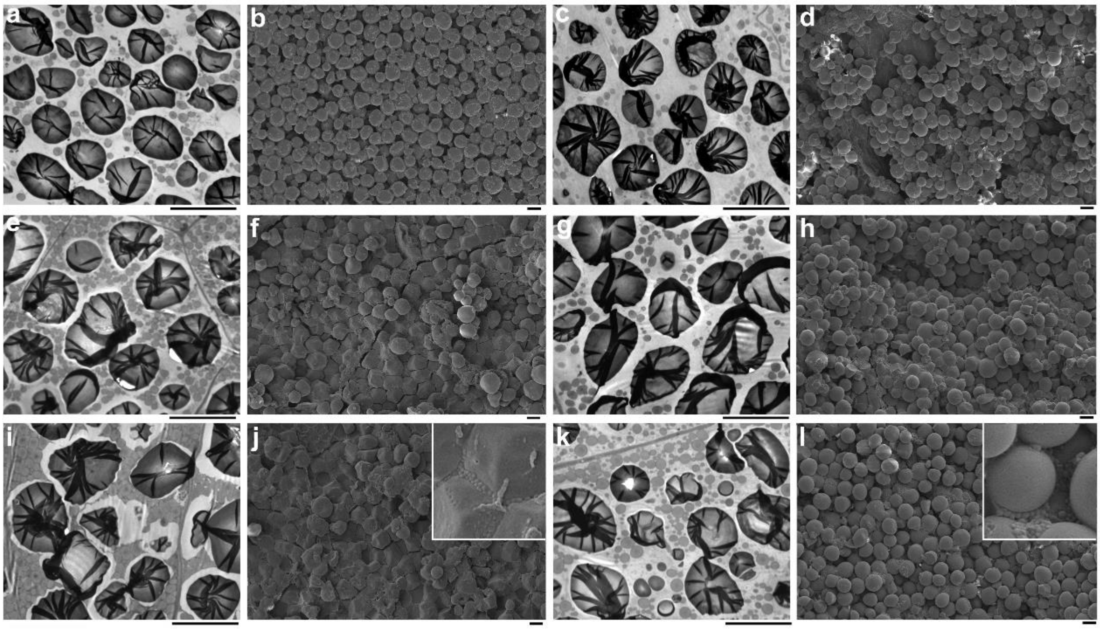


**Supplementary Figure 8. TEM and SEM observation of SGs and PBs in NILW64A and NIL619. a-b,** TEM (**a**) and SEM (**b**) analysis of SGs and PBs in 24-DAP endosperms of NILW64A. **c-d,** TEM (**c**) and SEM (**d**) analysis of SGs and PBs in 24-DAP endosperms of NILA619. **e-f,** TEM (**e**) and SEM (**f**) analysis of SGs and PBs in 30-DAP endosperms of NILW64A. **g-h,** TEM (**g**) and SEM (**h**) analysis of SGs and PBs in 30-DAP endosperm of NILA619. **i-j,** TEM (**i**) and SEM (**j**) analysis of SGs and PBs in 35-DAP endosperms of NILW64A. **k-l,** TEM (**k**) and SEM (**l**) analysis of SGs and PBs in 35-DAP endosperms of NILA619. Scale bar, 10 μm.

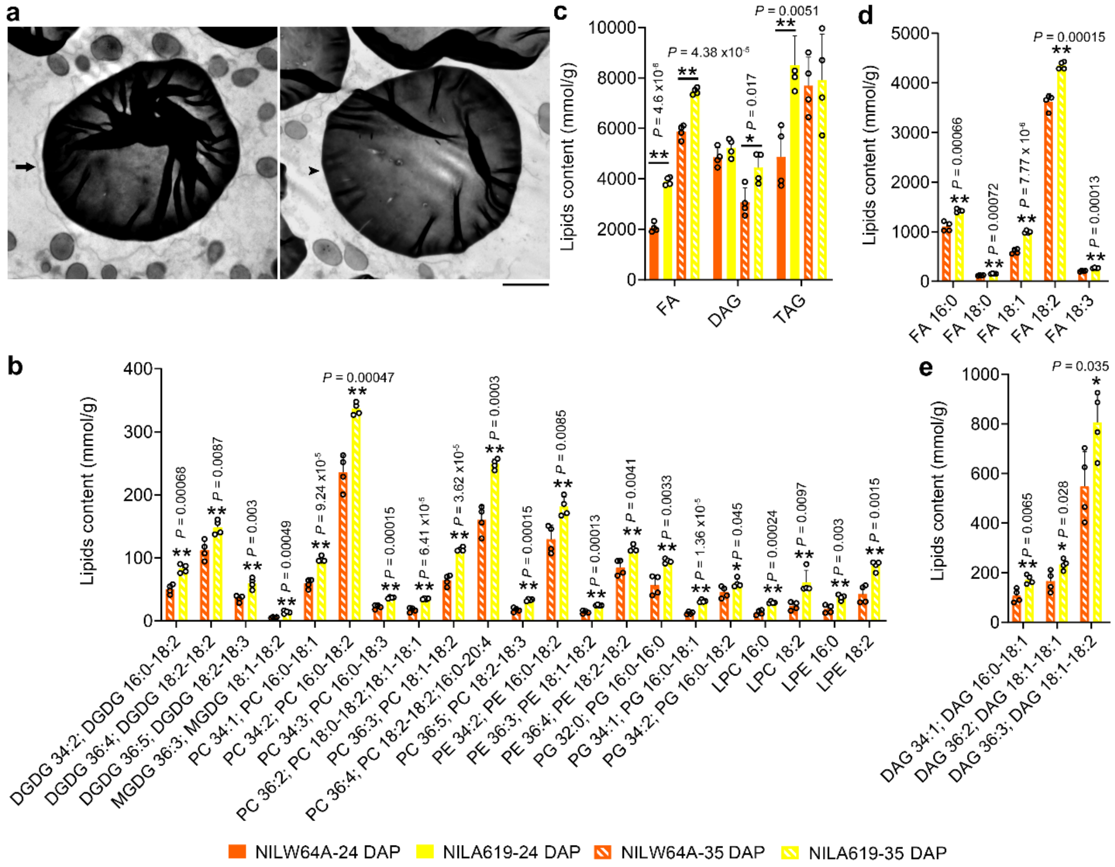

**Supplementary Figure 9. Analysis of lipid content in endosperm of NILs.**

**a,** SGs with membranes in integrity (indicated by the arrow) and complete degradation (indicated by the arrowhead) in NILW64A at 24 DAP. Scale bar, 2 μm. **b,** Contents of individual molecular species of DGDG, MGDG, PC, PE, PG, LPC and LPE in NILs at 35 DAP. n=4 biological replicates. * and **, significant differences at *P* < 0.05 and *P* < 0.01 in two-sided Student's *t*-test, respectively. **c,** The content of neutral lipids in endosperms of NILs at 24 DAP and 35 DAP. n=4 biological replicates. * and **, significant differences at *P* < 0.05 and *P* < 0.01 in two-sided Student's *t*-test, respectively. **d-e,** Contents of individual molecular species of FA (**d**) and DAG (**e**) in NILs at 35 DAP. Data are shown as means ± SD, n=4 biological replicates. * and **, significant differences at *P* < 0.05 and *P* < 0.01 in two-sided Student's *t*-test, respectively. The source data underlying Supplementary Figure 9b-e are provided as a Source Data file.

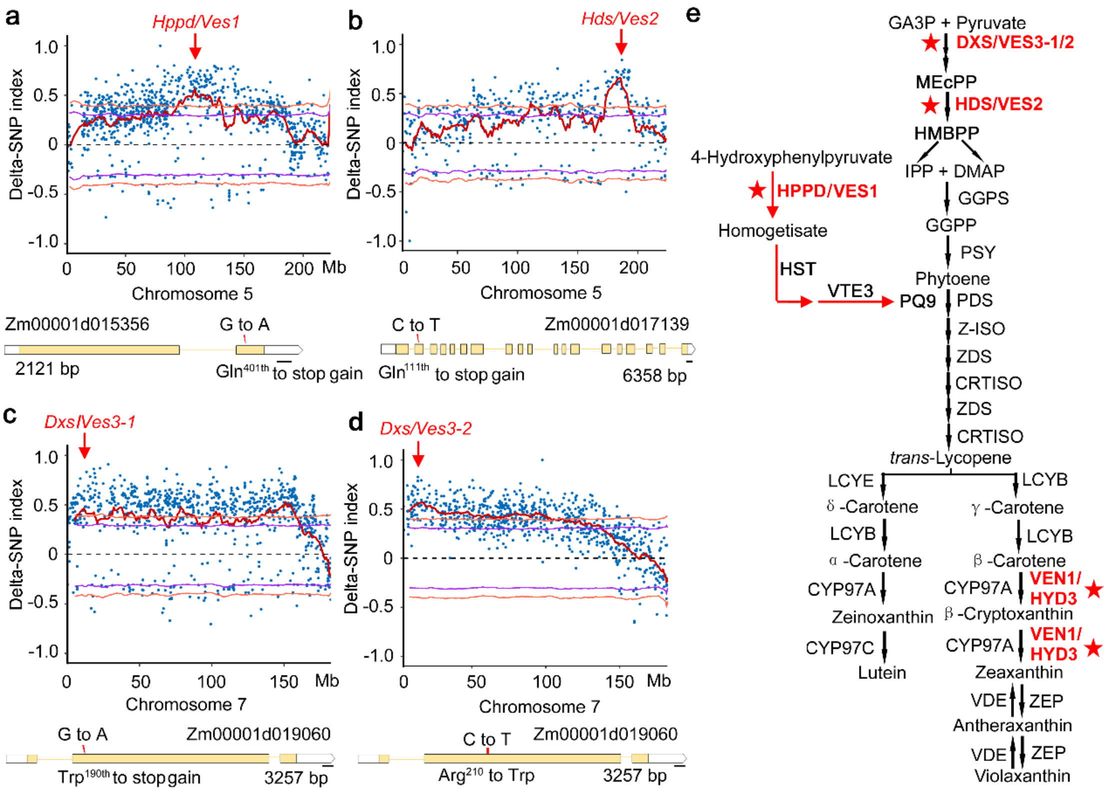


**Supplementary Figure 10. Mapping-by-sequencing of the suppressors.** Delta SNP index of two pools of mutant and wild type kernels segregated from $F_2$ of the suppressors. The red arrowhead indicates the candidate region. The red line is the mean value of SNP-index, purple is the threshold line of 95% confidence level, and orange is 99% confidence level. Scale bar, 100 bp.

**a,** Mapping-by-sequencing of *ves1* and the gene structure. **b,** Mapping-by-sequencing of *ves2* and the gene structure. **c,** Mapping-by-sequencing of *ves3-1* and the gene structure. **d,** Mapping-by-sequencing of *ves3-2* and the gene structure. **e**, The metabolic pathways of carotene. The red asterisks indicate the positions of the suppressors and *Ven1* in the carotenoid biosynthetic pathway.

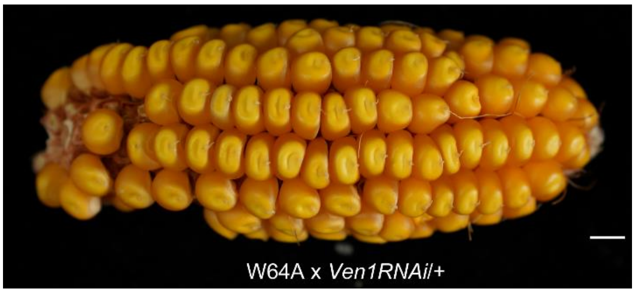


**Supplementary Figure 11. Ear phenotype of W64A x *Ven1RNAi/+*.** All progeny seeds are vitreous. Scale bar, 1 cm.

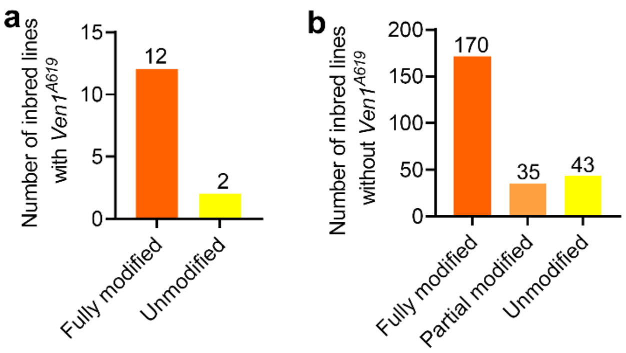


**Supplementary Figure 12. Number of inbred lines with or without *Ven1* modifiers.**
**a,** Test of modifiers in inbred lines with *Ven1*$^{A619}$. **b,** Test of modifiers in inbred lines without *Ven1*$^{A619}$. See Supplementary Data 1 for *Ven1* genotype in the 262 inbred lines.

**Supplementary Table 1. RNA-seq data of nine candidate genes in W64A and A619 endosperms at 18 DAP.**

| Start | Gene-symbol | RNA-seq Reads in A619 18 DAP En | RNA-seq Reads in W64A 18 DAP En | Description | Function | Reference |
|---|---|---|---|---|---|---|
| Zm00001d030314 | *Prpl1* | 348.027 | 3071.832 | Proline-rich protein like protein | Controls elongation of root hairs in Arabidopsis thaliana | Boron *et al.*[1] |
| Zm00001d032900 | *Prcp* | 234.801 | 610.819 | Pentatricopeptide repeat-containing protein | Required for chloroplast protein synthesis and accumulation of subunits of the thylakoid protein complexes. | Ferrari *et al.*[2] |
| Zm00001d041726 | *Dmr6* | 356.781 | 3894.344 | 2-oxoglutarate (2OG)-Fe(II) oxygenase; | Converts salicylic acid (SA) to 2,3-dihydroxybenzoic acid (2,3-DHBA) (By similarity). Suppressor of immunity. Regulates negatively defense associated genes expression | van Damme *et al.*[3] |
| Zm00001d038218 | *c2* | 281.160 | 520.240 | Cytochrome c-2 | Electron carrier protein | De March *et al.*[4] |
| Zm00001d011168 | *Zep* | 121.117 | 203.693 | ZEP | Zeaxanthin epoxidase converts zeaxanthin into antheraxanthin and subsequently violaxanthin. | Niyogi *et al.*[5] |
| Zm00001d011174 | *See2a* | 0.319 | 268.897 | Senescence enhanced2a | Involved in PCD-related processes such as protein degradation | Yandeau-Nelson *et al.*[6] |

| | | | | | | |
|---|---|---|---|---|---|---|
| Zm00001d011183 | *Thi1* | 1.324 | 133.874 | Thiamine biosynthesis 1 | Bifunctional enzyme that catalyzes the phosphorylation of hydroxymethylpyrimidine phosphate (HMP-P) to HMP-PP and condenses 4-methyl-5-(beta-hydroxyethyl)thiazole monophosphate (THZ-P) and 2-methyl-4-amino-5-hydroxymethyl pyrimidine pyrophosphate (HMP-PP) to form thiamine monophosphate (TMP). | Komeda *et al.*[7] |
| Zm00001d011253 | *Snf4* | 1.717 | 873.361 | Sucrose nonfermenting 4-like protein | Regulatory subunit of the probable trimeric SNF1-related protein kinase (SnRK) complex,plays a key role in the global control of plant carbon metabolism . | Halford *et al.*[8] |
| Zm00001d012675 | *Gst1* | 2145.166 | 5216.705 | Glutathione S-transferase 1 | Catalyzes the conjugation of glutathione (GSH; γ-Glu–Cys–Gly) into electrophilic compounds such as ROS and plays major roles in the detoxification of xenobiotics and oxidative stress metabolism. | Labrou *et al*.[9]; Liu *et al*.[10] |

**Supplemental Table 2. Primers used in this study.**

| Primer name | Sequence (5'-3') | Usage |
|---|---|---|
| *Ven1*-GF | ACTCATGACGTTGGCAGTGT | Full-length genomic sequence and CDS |
| *Ven1*-GR | CTCCTCCAGCTCCATGTACG | |
| *Ven1*-CF | CACTCTGCCTTCCCCTCCTAT | |
| *Ven1W64A*-CR | TGAAAGGAAGATGGCGATAGATGTA | |
| *Ven1A619*-CR | GATGGCCCTTCTGGTTCGCG | |
| *Ven1*-RTR | TACCCTGTCCATCCGTAGCG | Real-time PCR |
| *Ven1*-RTR | GGCGACGAAGCTGGTCATC | |
| Actin-RTF | GCTACGAGATGCCTGATGGTC | |
| Actin-RTR | CCCCCACTGAGGACAACG | |
| *Ven1*-probeSac1 | CGAGCTCAACGACGTGTTCGCCATCGTC | *In situ* probe |
| *Ven1*-probeBamH1 | CGGGATCCTGTTCGGACAACGCATACAAC | |
| *Ven1*-KF | GGGGTACCATGGCCGCCGCGATGACCAGCTT | Subcellular localization |
| *Ven1*-BF | CGGGATCCGAACTCATTTGGCACACTCT GCC | |
| *Ven1*-AFBspE1 | CGGTCCGGAAGCTCAACGACGTGTTCGCC | RNAi vector construction and Identification |
| *Ven1*-ARBamH1 | CGGGATCCTGTTCGGACAACGCATACAAC | |
| *Ven1*-SF2Xba1 | GCTCTAGAAGCTCAACGACGTGTTCGCC | |
| *Ven1*-SR2Sac1 | CGAGCTCTGTTCGGACAACGCATACAAC | |
| P27RNAi –F | ATGCTTACAGCTCACAAGAC | |
| GFPRNAi –R | TGAACTTGTGGCCGTTTAC | |
| 134.7M-F | ATCTGCCAGGTAGCTGTTGG | Fine mapping of *Ven1* |
| 134.7M-R | GTTCTCCTGCTCCTCCTCCT | |
| 135.6M-F | GGTGGTGATTGGTGTTCTCC | |
| 135.6M-R | CGTGCGGCTACTTCTACCAT | |
| 137.0M-F | GAGCATTGCGGAACAGATG | |
| 137.0M-R | GAACGAGGAAAGCAATAGCG | |
| 137.1M-F | GGACGAAACAACAAAACACAGGA | |
| 137.1M-R | GAAATGGTTGCACATCTGGGG | |
| 137.3M-F | TCTACAGCCAGTGTGGGACA | |
| 137.3M-R | CGAGAGTGGTTCCCGTGTAT | |
| 138.3M-F | TGCACCGCAATTAAACACAT | |
| 138.3M-R | CCAAGACCAAGAGTCGGAAG | |
| 139.3M-F | CTCGCTGGATGTTAGAGGGG | |
| 139.3M-R | ACAGCCACTGCCTTCTGC | |
| 141.8M-F | CACAAGCAACAGCTCGACTC | |
| 141.8M-R | TATGGCTTGTTCTCAACCCC | |

## Supplementary References


1. Boron AK*, et al.* Proline-rich protein-like PRPL1 controls elongation of root hairs in *Arabidopsis thaliana*. *J Exp Bot* **65**, 5485-5495 (2014).

2. Ferrari R*, et al.* CRP1 Protein: (dis)similarities between *Arabidopsis thaliana* and *Zea mays*. *Front Plant Sci* **8**, 163 (2017).

3. van Damme M, Huibers RP, Elberse J, Van den Ackerveken G. Arabidopsis DMR6 encodes a putative 2OG-Fe(II) oxygenase that is defense-associated but required for susceptibility to downy mildew. *Plant J* **54**, 785-793 (2008).

4. De March M, Brancatelli G, Demitri N, De Zorzi R, Hickey N, Geremia S. A general exit strategy of monoheme cytochromes c and c2 in electron transfer complexes? *IUBMB Life* **67**, 694-700 (2015).

5. Niyogi KK, Grossman AR, Bjorkman O. Arabidopsis mutants define a central role for the xanthophyll cycle in the regulation of photosynthetic energy conversion. *Plant Cell* **10**, 1121-1134 (1998).

6. Yandeau-Nelson MD, Laurens L, Shi Z, Xia H, Smith AM, Guiltinan MJ. Starch-branching enzyme IIa is required for proper diurnal cycling of starch in leaves of maize. *Plant Physiology* **156**, 479-490 (2011).

7. Komeda Y, Tanaka M, Nishimune T. A *th-1* mutant of *Arabidopsis thaliana* is defective for a thiamin-phosphate-synthesizing enzyme: thiamin phosphate pyrophosphorylase. *Plant Physiology* **88**, 248-250 (1988).

8. Halford NG*, et al.* Metabolic signalling and carbon partitioning: role of Snf1-related (SnRK1) protein kinase. *J Exp Bot* **54**, 467-475 (2003).

9. Labrou NE, Papageorgiou AC, Pavli O, Flemetakis E. Plant GSTome: structure and functional role in xenome network and plant stress response. *Curr Opin Biotechnol* **32**, 186-194 (2015).

10. Liu YJ, Han XM, Ren LL, Yang HL, Zeng QY. Functional divergence of the glutathione *S*-transferase supergene family in *Physcomitrella patens* reveals complex patterns of large gene family evolution in land plants. *Plant Physiology* **161**, 773-786 (2013).
