# Supplementary material for: Carotenoids modulate kernel texture in maize by influencing amyloplast envelope integrity
Source: Nat Commun. 2020 Oct 22;11:5346. doi: 10.1038/s41467-020-19196-9 (PMC7582188; doi:10.1038/s41467-020-19196-9)
Supplement: Supplementary file 4 — Description of Additional Supplementary Files [file 41467_2020_19196_MOESM4_ESM.pdf]

Description of Additional Supplementary Files

Supplementary Data 1

Ven1 genotype in the 262 inbred lines
