# Supplementary material for: Carotenoids modulate kernel texture in maize by influencing amyloplast envelope integrity
Source: Nat Commun. 2020 Oct 22;11:5346. doi: 10.1038/s41467-020-19196-9 (PMC7582188; doi:10.1038/s41467-020-19196-9)
Supplement: Supplementary file 2 — Peer Review File [file 41467_2020_19196_MOESM2_ESM.pdf]

Reviewer #1 (Remarks to the Author):

This manuscript focuses on the mechanistic basis of vitreous vs. opaque endosperm, implicating the direct role of polar vs. nonpolar carotenoids in mediating the vitreous phenotype through physicochemical changes in the amyloplast membrane.

Major point:
1. The authors postulate that carotenoid composition influences the physicochemical properties of amyloplast envelope membranes and as a result, there is an alteration in the interactions between the starch grains and protein bodies which affects the vitreous phenotype. While carotenoid composition correlates with the changes in these endosperm properties, no evidence is presented to support a causative and direct role of carotenoids. Moreover, even in the mutant there is a significant amount of both polar and nonpolar carotenoids, so the hypothesis is weak. An alternate hypothesis is that the increase in betacarotene leads to some other metabolic change which is responsible. For example, carotenoids are precursors to hormones and to many other apocarotenoids (including many new apocarotenoids recently discovered, such as beta-cyclocitral derived from betacarotene, strigolactones, and others) which might influence this interaction (see: Plant Physiology [2019] 179 (3): 830–843). In addition, it is known that modification of carotenoid composition can lead to pleiotropic effects on primary metabolism (as supported by numerous papers). Thus, the data presented do not show a direct effect of carotenoids on the quality of the vitreous phenotype. At this time, it is too speculative to suggest a mechanism involving the physical properties of the polar or nonpolar carotenoids in the amyloplast membrane as being responsible for the altered interaction with the protein bodies. In addition, the carotenoids themselves are not simply grouped into polar vs. nonpolar, which becomes obvious when examining the actual structures that form during carotenoid biosynthesis (see: Plant Physiology [2019] 179 (3): 830–843); Moise et al [2014] Chemical Reviews 114:164-19).

Some other points:
2. If the Ven1 mutant is a deletion, how is it that any xanthophylls accumulate? It would help if more details were provided on the molecular changes in the gene and putative gene product. The authors are apparently not aware of the early work on HYD3 and the A619 line that appeared online in 2009, but instead cite a later 2010 paper by Yan et al. This paper should be cited instead, see:
a. Vallabhaneni et al. (2009) Metabolite sorting of a germplasm collection reveals the Hydroxylase3 locus as a new target for maize provitamin A biofortification. Plant Physiology 151 (3): 1635-1645.

3. Abstract:
a. "integrity of amyloplast membranes is increased"- what is meant by integrity? Fluidity?
b. "lipid composition in endosperm cells in A619 is altered, giving rise to a persistent amyloplast envelope."- what is meant by "persistent"?
c. "These changes prevent protein bodies from interacting with starch grains, creating air spaces that cause an opaque kernel phenotype"- the authors should make clear in the writing what is the connection between the amyloplast envelope membrane composition and interaction between protein bodies and starch grains.

4. Supplementary Fig. 10 and other references to the pathway should be corrected:
a. CYP97A and CYP97C are missing on the lutein side. Both enzymes are needed for lutein biosynthesis. See:
i. Quinlan et al. (2012) Plant Physiology 160: 204–214.
b. Delete LUT2; use LCYE which is the nomenclature for maize.
c. the figure is missing an important new pathway enzyme Z-ISO. See:
i. Beltrán et al. (2015) Control of carotenoid biosynthesis through a heme-based cis-trans isomerase. Nature Chemical Biology 11(8):598-605
ii. Chen et al. (2010) Isolation and characterization of the Z-ISO gene encoding a missing component of carotenoid biosynthesis in plants. Plant Physiology 153: 66-79

iii. Li et al. (2007) Maize Y9 is essential for 15-cis-zetacarotene isomerization. Plant Physiology 144 (2): 1181-1189.

5. Discussion
a. "The altered carotenoid composition of NILA619 endosperm protects amyloplast membranes from breaking down."
No direct evidence is provided to support this statement.

b. "Although these mutations affect many other secondary metabolites, they all have the common feature that the levels of lycopene, alpha carotene and beta carotene are concurrently reduced, indicating that upstream carotenoid biosynthetic genes are epistatic to the Ven1A619 phenotype."
The interpretation is simplistic.

c. "Except for their role in ABA production, which directly affects seed dormancy, carotenoids appear dispensable for the biological functions of amyloplasts, at least in terms of starch synthesis. This is supported by many high yielding commercial white maize varieties, where carotenoid biosynthesis does not occur."

Carotenoid biosynthesis must occur- this statement is false. If there were no carotenoids at all in the endosperm, then the resulting plant would be lethal, which is not the case. White varieties do make small amounts of carotenoids, especially to support ABA biosynthesis (just as the progenitor teosinte, which carries the ancestral state of the carotenoid alleles). We are learning now that there are many apocarotenoid derivatives with important hormonal and biological functions, besides ABA. At this point, it is not possible to state that the change in composition of polar vs nonpolar carotenoids is mediating the effect on kernel vitreous phenotypes. The papers that support this hypothesis describe experiments using artificial bilayers. Moreover, the inbreds also differed in lipid composition.

6. Fix multiple typos.

Reviewer #2 (Remarks to the Author):

This work reports a new relationship between carotenoid biosynthesis and endosperm texture (vitreousness). Especially a major QTL (vitreous endosperm 1, i.e. ven1) was highlighted in dent inbred lines and the corresponding gene was identified through backcrossing. This gene encodes a β-carotene 3-hydroxylase, an enzyme that produces zeaxanthin by hydroxylation at 3 and 3' positions 3 of β-ionone rings. Absence of the expression of this enzyme in the opaque/floury inbred lines (A619) and corresponding near-isogenic line (NILA619) led to a significant decrease of zeaxanthin content and an expected increase of the β-carotene content. On the contrary, the opposite is observed for the vitreous inbred line (W64A) and its corresponding isogenic line (NILW64A). This led to significant differences in the ratio of the polar vs. nonpolar carotenoid contents between vitreous and opaque lines while total carotenoid contents of the vitreous and opaque lines are identical. The correlation between endosperm vitreousness and the polar vs. nonpolar carotenoid ratio is unquestionable and clearly demonstrated. Especially, knock-down of Ven1 expression in another vitreous inbred line (KN5585) induced an opaque endosperm. This work provides also interesting data and tools to investigate the carotenoid metabolism which is still poorly understood.

However, it should be pointed out that zeaxanthin synthesis takes place in the opaque lines, even if at a lower level. Is there another redundant gene (β-carotene hydroxylase) that could be involved in zeaxanthin synthesis? Using BLAST, a highly homologous gene Zm00001d002589 (hyd4 - hydroxylase4) is expressed (although weakly) in the endosperm. This is not commented in the

manuscript.

The molecular and physicochemical causes of this effect were further explored. By different approaches including electron microscopy (TEM) and lipid analyses (UPLC/mass spectrometry), the authors conclude that these changes of the carotenoid composition impact the stability of amyloplast membranes. This conclusion is however questionable.

First of all, this hypothesis is only based on TEM observations. The authors observed some discontinuities in the amyloplast membranes of the vitreous lines that are correlated with adhesion of protein bodies to starch granules. However owing to the particular hard texture of the vitreous endosperm, fixation and sectioning could induce membrane disruption. Secondly, to strengthen this hypothesis, no changes in protein and starch accumulation were reported between the vitreous and floury lines. The zein contents and compositions seem strictly identical on the basis of SDS-PAGE. However, in supplementary fig.6, only zein bands are highlighted and changes in the content of the other proteins (albumins, globulins and glutelins) cannot be determined. Furthermore, SDS-PAGE is not as sensitive as a chemical or colorimetric assay (e.g., a nitrogen assay would be compatible with the extraction buffer). The composition of the storage carbohydrate polymers was not determined, while the ratio of the amylose to amylopectin ratio could strongly impact endosperm vitreousness. It should be noted that this ratio may vary even if the starch content does not change. As mentioned by the authors (beginning of the discussion), the high-amylose maize are vitreous while the waxy (high amylopectin maize) are floury. This was also observed in maize inbred lines (ref 4) where the vitreous regions of the maize endosperms have higher amylose contents than the floury ones. It is difficult to predict this ratio on the basis of the transcript level of starch biosynthetic enzymes (supplementary fig.7).Indeed, the activity of these enzymes is highly regulated by post-translational modifications (phosphorylation, formation of protein-protein complexes).
Furthermore considering that carotenoids and starch polymers are synthesized in the same subcellular compartment, i.e., the amyloplast, interference between these metabolic pathways could occur.

Finally, in regard to the suggested degradation of amyloplast membranes, the lipids were analyzed in the vitreous and opaque lines. Lipid analyses has to be performed, since carotenoids and lipids compete for the same carbon flux (and starch as well). The main drawback of these lipid analyses is the endosperm isolation. In this work, the endosperm was obtained after seed coat and embryo removal. This leaves most of the aleurone cell layer surrounding the starchy endosperm. The aleurone cells contain high amount of lipids, especially storage lipids, i.e. triacylglycerols (TAG), while most of the TAG of the starchy endosperm are degraded into free fatty acids during endosperm development (Tan, S. L., Morrison, W. R. Distribution of lipids in the germ, endosperm, pericarp and tip cap of amylomaize, LG-11 hybrid maize and waxy maize. J. Am. Oil Chem. Soc. 1979, 56, 531−535); Rolletschek et al. Positional cues for the starch/lipid balance in maize kernels and resource partitioning to the embryo. Plant J. 2005, 42, 69-83). The high amounts of TAG reported in fig.3 suggest that endosperm is highly contaminated by aleurone cells. Although this contamination is not a major problem for the main storage compounds, i.e., proteins and starch (aleurone cells do not accumulate zeins and starch), this is not the case for storage lipids which are about thirty times more concentrated in the aleurone layer than in the starchy endosperm. It is only possible to conclude for the free fatty acids (FA) and galactosyldiacylglycerol (DGDG, MGDG) contents, which concentrate and are specific of the starchy endosperm, respectively. Concerning galactosyldiacylglycerol, it is surprising to observe very low amounts of MGDG (fig. 3k) while previous studies reports similar contents (Tan & Morrison, 1979 cited above; Gayral, et al. Lipid partitioning in maize (Zea mays L.) endosperm highlights relationships among starch lipids, amylose, and vitreousness. Journal of agricultural and food chemistry, 2015, 63, 3551-3558). In this regard, it was not specified whether standards were used to quantify lipids in UPLC-mass spectrometry in the Methods section. Furthermore, lipids were extracted using CH2Cl2-MeOH and 2-propanol, a procedure that does not extract lipids embedded within starch granules. These lipids can be extracted by hot 1-propanol-water (see Gayral et al, 2015, cited above). Actually, starch lipids account for about 50% of total lipids of maize starchy endosperm and are composed of free fatty acids (FA) and lysophospholipids (LPC, LPE).

These starch-bound lipids are a specificity of cereal starches and are related to the cell death (PCD, programmed cell death) development of cereal endosperms, which induces a specific endoplasmic reticulum (ER)-plastid trafficking. This trafficking drives MGDG and DGDG synthesis as well as the amylose to amylopectin ratio and finely is correlated to endosperm vitreousness (see Gayral et al, 2015 cited above and Gayral et al The spatiotemporal deposition of lysophosphatidylcholine within starch granules of maize endosperm and its relationships to the expression of genes involved in endoplasmic reticulum–amyloplast lipid trafficking and galactolipid synthesis. Plant Cell Physiol., 2019, 60, 139-151). It could be expected that changes in the carotenoid composition impact this lipid trafficking. Carotenoid trafficking can also occur between ER and amyloplast as observed for chloroplasts and changes in the carotenoid composition can also modify this trafficking. This can impact fatty acid desaturation in the ER as observed in this work (Mehrshahi et al. Transorganellar complementation redefines the biochemical continuity of endoplasmic reticulum and chloroplasts. Proc Natl Acad Sci U S A. 2013, 12126-12131). It is therefore essential to determine both the amylose-amylopectin ratio as well as the composition of starch lipids in the vitreous and opaque inbred lines.

Finally the authors should consider that more than "membrane degradation" the impact of the modifications of the carotenoid biosynthetic pathway can induce huge modifications of the endosperm metabolism. Especially, changing the carotenoid composition and probably the ER-amyloplast interactions and ER-amyloplast trafficking should amplify the ER stress and the UPR responses that drive endosperm PCD and endosperm metabolism (Shank et al. Induction of lipid metabolic enzymes during the endoplasmic reticulum stress response in plants. Plant Physiol. 2001, 126, 267-277; Gayral et al. Responses to hypoxia and endoplasmic reticulum stress discriminate the development of vitreous and floury endosperms of conventional maize (Zea mays) inbred lines. Front Plant Sci. 2017, 8:557). Increase of storage lipids (TAG) and-or their hydrolysis products (FA) in NILA619 was also observed in the opaque floury2 maize (Shank et al, 2001) or in the opaque regions of maize inbred lines (Rolletschek et al, 2005; Gayral et al, 2015) and was related to a boost of UPR (Shank et al, 2001; Gayral et al, 2017).

Other comments:
In NILA619 endosperm, the formation of multilamellar vesicles could support an ER-phagy process (Fig 3f) that strengthens an increase of stress responses in opaque maize (similar to autophagy)(Bernales et al (2007). ER-phagy: selective autophagy of the endoplasmic reticulum. Autophagy, 2007, 3, 285-287). Autophagy is triggered by UPR in agreement with the comments done above. These lamellar structures could trap protein bodies (Fig 3g), thus preventing fusion of PBs. The fusion of protein bodies could be a prerequisite for the adhesion of the protein bodies to the surface of the starch granules.

It is questionable whether in fact the kinetics of events (UPR, autophagy) leading to cell death (contributing to PCD) would be earlier in opaque lines than in vitreous lines. This would be a common molecular mechanism induced here by changes in the carotenoid composition and by other metabolic changes in other opaque lines (or in opaque regions of dent lines). Indeed the extent of vitreousness could be the result of a balance between the management of stress responses during endosperm development.

In fig1c, SEM was performed on the vitreous region of the endosperm of W64A and NILW64A while SEM was performed on the opaque region of A619 and NILA619. It would have been more pertinent to compare the vitreous and opaque regions of the different maize line. Eventually SEM at the interface of the opaque-vitreous regions of each line should give more information on the relationship between carotenoid composition and packing of PBs and SG in the endosperm.

At this stage of the data, fig 5e is quite speculative

There are many typing errors in the manuscript:
About quantitative trait the loci >> About quantitative trait loci

« . ..ranging from nearly completely vitreous to completely starchy.” Use “opaque” or “floury” instead of starchy; starchy could suggest that the endosperm contains only starch.

“Although the reason is unclear, this mutation affects monogalactosyldiacylglcerol,
the enzyme responsible for synthesis of amyloplast membrane
phospholipids ». Although the reason is unclear, this mutation affects monogalactosyldiacylglcerol synthase, the enzyme responsible for synthesis of amyloplast membrane galactolipids

Use membrane disruption instead of membrane degradation; degradation means that components are metabolically transformed. Here, it’s more a mechanical effect.

Reviewer #3 (Remarks to the Author):

Wang et al.
This paper presents genetic evidence implicating carotenoid metabolism in determination of endosperm texture in maize. Vitreous and non-vitreous (opaque) endosperm phenotypes, which have been studied extensively in maize, have uncovered deep connections among diverse metabolic and cellular processes that affect concretion of protein bodies and starch grains in the mature endosperm. The new results show that carotenoid pigments that accumulate in the amyloplast envelop membranes affect membrane stability during endosperm maturation.

A recessive non-vitreous factor in inbred A619 is identified as a partial deletion of a carotenoid 3-hydroxylase involved in biosynthesis of xanthophylls. Accumulation of non-polar carotenoids in the Ven1-A619 background is proposed to stabilize amyloplast envelop membranes delaying degradation and preventing contact between starch grains and protein bodies. In support of this hypothesis, several carotenoid deficient mutants recovered as suppressors are shown to be epistatic to the Ven1-A619 mutation. One complication is that the suppressors identified are pleiotropic with effects that are not limited to carotenoids.

Regardless of the mechanism, the Ven1 phenotype reveals an unexpected barrier for efforts directed toward increasing pro vitamin A content of maize grain.

For these reasons, the results are novel and will be of substantial interest to the plant genetics and breeding communities.

Specific comments

The suppressor screen uncovered four mutants in known genes that have carotenoid deficient phenotypes. However, all of them are in enzymes that are not specific to the carotenoid pathway affecting tocopherol biosynthesis and other isoprenoid pathways. While the screen may not have reached saturation, there are at least a comparable number of known loci that are specific to the carotenoid pathway that would also be predicted to suppress Ven1. Is y1 (phytoene synthase) also epistatic to Ven1? In addition to being more specific to the non-polar carotenoids, y1 is the principle genetic determinant of carotenoid content in breeding lines.

The evidence implicating non-polar carotenoids specifically is indirect. The authors dismiss the possibility that a product of Ven1 could cause membrane degradation on the basis that suppressors are carotenoid deficient. However, suppose for example that in vitreous endosperm membrane

degradation is induced by an interaction between xanthophylls and tocopherols. In that case, Ven1-A619 would block degradation by blocking synthesis of xanthophylls whereas suppressors that reduce tocopherols obviate the need for xanthophylls. In any case, the apparent complexity of genetic background effects suggests that the Ven1-A619 phenotype requires more than accumulation of carotenoids.

Regarding the sentence containing “Since protein content is usually determined by the maternal genotype and endosperm vitreousness by the filial genotype”. This statement implying endosperm protein content is generally under maternal control is made without a citation. What is the basis for this statement?

The nomenclature is non-standard for maize genetics. The naming of suppressors is especially confusing as these have the form of allele designations.

Don McCarty

Reviewer #1 (Remarks to the Author):

This manuscript focuses on the mechanistic basis of vitreous vs. opaque endosperm, implicating the direct role of polar vs. nonpolar carotenoids in mediating the vitreous phenotype through physicochemical changes in the amyloplast membrane.

Major point:

1. The authors postulate that carotenoid composition influences the physicochemical properties of amyloplast envelope membranes and as a result, there is an alteration in the interactions between the starch grains and protein bodies which affects the vitreous phenotype. While carotenoid composition correlates with the changes in these endosperm properties, no evidence is presented to support a causative and direct role of carotenoids. Moreover, even in the mutant there is a significant amount of both polar and nonpolar carotenoids, so the hypothesis is weak. An alternate hypothesis is that the increase in betacarotene leads to some other metabolic change which is responsible. For example, carotenoids are precursors to hormones and to many other apocarotenoids (including many new apocarotenoids recently discovered, such as beta-cyclocitral derived from betacarotene, strigolactones, and others) which might influence this interaction (see: Plant Physiology [2019] 179 (3): 830–843). In addition, it is known that modification of carotenoid composition can lead to pleiotropic effects on primary metabolism (as supported by numerous papers). Thus, the data presented do not show a direct effect of carotenoids on the quality of the vitreous phenotype. At this time, it is too speculative to suggest a mechanism involving the physical properties of the polar or nonpolar carotenoids in the amyloplast membrane as being responsible for the altered interaction with the protein bodies. In addition, the carotenoids themselves are not simply grouped into polar vs. nonpolar, which becomes obvious when examining the actual structures that form during carotenoid biosynthesis (see: Plant Physiology [2019] 179 (3): 830–843); Moise et al [2014] Chemical Reviews 114:164-19).

Response: Thanks for your comment and suggestion. Carotenoids are arranged into two big subgroups with respect to the presence of oxygen

functional groups: nonpolar (apolar) carotenes (β-carotene, lycopene) and polar xanthophylls, containing hydroxyl, keto, or epoxy group, attached to one (β-cryptoxanthin) or at both ends of the molecule (lutein, zeaxanthin). (Popova, A.V. & Andreeva, A.S. Chapter Eight - Carotenoid–Lipid Interactions. in *Advances in Planar Lipid Bilayers and Liposomes*, Vol. 17, 215-236, 2013).

In this study, we cloned a major QTL, *Ven1* (encoding the β-carotene hydroxylase 3), that affects the important kernel trait, virtuousness. The A619 inbred contains a nonfunctional *Ven1* allele, leading to a decrease in polar and an increase in non-polar carotenoids in the amyloplast. The alteration in carotenoid composition or probably the downstream metabolites could influence the physicochemical properties of amyloplast envelope membranes as well as the lipid composition, as a result, affect the assembly of protein bodies and the interactions between protein bodies and starch grains, which determines the vitreous phenotype. As you suggested, we have added the following possibility in the Discussion of the revised manuscript: "Moreover, carotenoids are precursors to hormones and many other apocarotenoids (including many new apocarotenoids recently discovered, such as β-cyclocitral derived from β-carotene, strigolactones, and others)[16,41]. The contents of these apocarotenoids may be changed in the endosperm of NILs and suppressors, which might also influence the stability of amyloplast membranes.

Because our EMS mutagenesis did not reach saturation for genetic screen (otherwise it was a huge amount of field work), we couldn't precisely identify which compound was a causative factor that affects the vitreous endosperm formation in A619. At this stage, we were simply trying to offer a logical hypothesis based on the data. Maize kernel vitreousness is a complex phenotype and we were presenting new data that offers insight into how vitreous endosperm forms. The unsolved mechanism can be left for subsequent research.

Some other points:

2. If the Ven1 mutant is a deletion, how is it that any xanthophylls accumulate? It would help if more details were provided on the molecular changes in the

gene and putative gene product. The authors are apparently not aware of the early work on HYD3 and the A619 line that appeared online in 2009, but instead cite a later 2010 paper by Yan et al. This paper should be cited instead, see:

a. Vallabhaneni et al. (2009) Metabolite sorting of a germplasm collection reveals the Hydroxylase3 locus as a new target for maize provitamin A biofortification. Plant Physiology 151 (3): 1635-1645.

Response: Thanks for your comment. We are sorry for missing the important reference paper that reported *HYD3* and the A619 line earlier. This paper was cited and the reference 11 was replaced in the corresponding place of the revised manuscript.

Based on our RNA-seq data, the transcript level of *Ven1/HYD3* was dramatically reduced in NILA619 compared to NILW64A, while those of *HYD4*, *HYD5*, *HYD6*, *CYP97A*, *CYP97C* were not apparently affected. This may explain that xanthophylls accumulates in NILA619.

FPKM value of maize carotene hydroxylase genes in NILW64A and NILA619.

gene_id	gene_symbol	NILA619_1	NILA619_2	NILA619_3	NILW64A_1	NILW64A_2	NILW64A_3
**Zm00001d026056**	*Ven1/HYD3*	5.4328	6.22873	5.14103	18.9842	23.7906	16.9743
**Zm00001d002589**	*HYD4*	0.479365	0.642864	0.766779	0.488093	0.628979	0.456653
**Zm00001d048469**	*HYD5*	0.022482	0	0.177048	0.020934	0	0.042379
**Zm00001d027443**	*HYD6*	0.272699	0.071853	0.874325	0.126148	0.165311	0.25856
**Zm00001d018488**	*CYP97A*	3.58885	3.24915	3.37286	2.19734	3.19095	2.23842
**Zm00001d029822**	*CYP97C*	1.01872	0.606432	1.53814	0.975302	1.27565	0.722895

3. Abstract:

a. “integrity of amyloplast membranes is increased”- what is meant by integrity? Fluidity?

Response: Based on the TEM observation in Fig 3 (b, c, f and g), amyloplast membranes broke down in the vitreous NILW64A endosperm at 24 and 35 DAP, whereas they still maintained intact in the opaque NILA619 endosperm. We meant that the stability of amyloplast membranes is increased in A619. We have replaced the "integrity" with "stability" here in the revised manuscript.

b. "lipid composition in endosperm cells in A619 is altered, giving rise to a persistent amyloplast envelope."- what is meant by "persistent"?

Response: Amyloplast membranes did not break down in NILA619 endosperms even at 35 DAP, so the membranes were persistent in NILA619.

c. "These changes prevent protein bodies from interacting with starch grains, creating air spaces that cause an opaque kernel phenotype"- the authors should make clear in the writing what is the connection between the amyloplast envelope membrane composition and interaction between protein bodies and starch grains.

Response: We found that the changes in properties of amyloplast envelope membranes and probably the lipid composition as well could affect the assembly of protein bodies and the interactions between protein bodies and starch grains. We have rephrased this sentence as the following: These changes may impede the gathering of protein bodies and prevent them from interacting with starch grains.

4. Supplementary Fig. 10 and other references to the pathway should be corrected:

a. CYP97A and CYP97C are missing on the lutein side. Both enzymes are needed for lutein biosynthesis. See:

i. Quinlan et al. (2012) Plant Physiology 160: 204–214.

b. Delete LUT2; use LCYE which is the nomenclature for maize.

c. the figure is missing an important new pathway enzyme Z-ISO. See:

i. Beltrán et al. (2015) Control of carotenoid biosynthesis through a heme-based cis-trans isomerase. Nature Chemical Biology 11(8):598-605

ii. Chen et al. (2010) Isolation and characterization of the Z-ISO gene encoding a missing component of carotenoid biosynthesis in plants. Plant Physiology 153: 66-79

iii. Li et al. (2007) Maize Y9 is essential for 15-cis-zetacarotene isomerization. Plant Physiology 144 (2): 1181-1189.

Response: Thanks for your comment! In the new Supplementary Fig. 10, we have also cloned the other two suppressors. The second encodes a hydroxymethylbutenyl diphosphate synthase (HDS) converting 2C-methyl-D-erytrithol 2,4-cyclodiphosphate to 1-hydroxy-2-methyl-2-(E)-butenyl 4-diphosphate, and the forth is a missense variant allelic to the third suppressor encoding DXS. According to the standard nomenclature for maize genes, the four *ven1* suppressors were renamed *vitreous endosperm suppressor 1, 2, 3-1* and *3-2* (*ves1*, *ves2*, *ves3-1* and *ves3-2*). We have added all the information in the new Supplementary Fig. 10 according to your suggestions and cited the papers in the revised manuscript as follows:

"We then cloned these genes by BSA sequencing (Supplementary Fig. 10a-d). The carotenoid biosynthesis pathway is conserved in maize and other plants, and the candidate genes, quantitative trait loci and phenotypic loci were well characterized[12-17]. According to these reports, we summarized the maize carotenoid biosynthesis pathway and included the suppressors in Supplementary Fig. 10e."

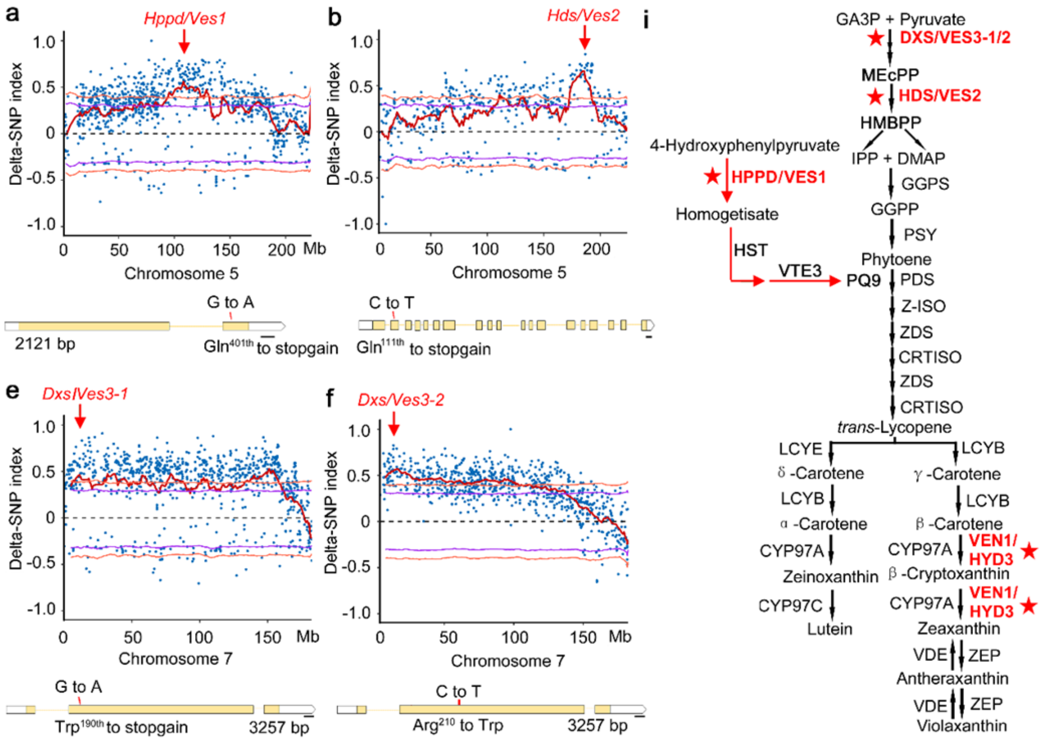

5. Discussion

a. “The altered carotenoid composition of NILA619 endosperm protects amyloplast membranes from breaking down.”

No direct evidence is provided to support this statement.

Response: Thanks for your comment! We have rephrased this sentence as follows: The altered carotenoid composition of NILA619 endosperm is associated with persistent amyloplast membranes.

b. “Although these mutations affect many other secondary metabolites, they all have the common feature that the levels of lycopene, alpha carotene and beta carotene are concurrently reduced, indicating that upstream carotenoid biosynthetic genes are epistatic to the Ven1A619 phenotype.”

The interpretation is simplistic.

Response: Thanks for your suggestion. We have rephrased this sentence as follows:

Although these mutations affect many other secondary metabolites such as tocopherol biosynthesis, apocarotenoid derivatives and other isoprenoids, they all have the common feature that the levels of lycopene, α-carotene and β-carotene are concurrently reduced, indicating that upstream carotenoid biosynthetic genes are epistatic to the *Ven1*^{A619} phenotype and the altered carotenoid composition may trigger an unknown physicochemical or metabolic mechanism to protect amyloplast membranes from breaking down.

c. "Except for their role in ABA production, which directly affects seed dormancy, carotenoids appear dispensable for the biological functions of amyloplasts, at least in terms of starch synthesis. This is supported by many high yielding commercial white maize varieties, where carotenoid biosynthesis does not occur."

Carotenoid biosynthesis must occur- this statement is false. If there were no carotenoids at all in the endosperm, then the resulting plant would be lethal, which is not the case. White varieties do make small amounts of carotenoids, especially to support ABA biosynthesis (just as the progenitor teosinte, which carries the ancestral state of the carotenoid alleles). We are learning now that there are many apocarotenoid derivatives with important hormonal and biological functions, besides ABA. At this point, it is not possible to state that the change in composition of polar vs nonpolar carotenoids is mediating the effect on kernel vitreous phenotypes. The papers that support this hypothesis describe experiments using artificial bilayers. Moreover, the inbreds also differed in lipid composition.

Response: Thanks for your comment! We have corrected this as follows: These carotenoids appear dispensable for the biological functions of amyloplasts, at least in terms of starch synthesis, which is supported by many high yielding commercial white maize varieties containing residual levels of carotenoids."

Considering that apocarotenoid derivatives have important biological functions, which might influence vitreous endosperm formation, we have discussed this as you suggested above:

Moreover, carotenoids are precursors to hormones and many other apocarotenoids (including many new apocarotenoids recently discovered, such as β-cyclocitral derived from β-carotene, strigolactones, and others)[16,41]. The contents of these apocarotenoids may be changed in the endosperm of NILs and suppressors, which might also influence the stability of amyloplast membranes.

6. Fix multiple typos.

Thanks for the comment. We have checked and corrected the typos throughout the manuscript.

Reviewer #2 report:

1 This work reports a new relationship between carotenoid biosynthesis and endosperm texture (vitreousness). Especially a major QTL (vitreous endosperm 1, i.e. ven1) was highlighted in dent inbred lines and the corresponding gene was identified through backcrossing. This gene encodes a β-carotene 3-hydroxylase, an enzyme that produces zeaxanthin by hydroxylation at 3 and 3' positions 3 of β-ionone rings. Absence of the expression of this enzyme in the opaque/floury inbred lines (A619) and corresponding near-isogenic line (NILA619) led to a significant decrease of zeaxanthin content and an expected increase of the β-carotene content. On the contrary, the opposite is observed for the vitreous inbred line (W64A) and its corresponding isogenic line (NILW64A). This led to significant differences in the ratio of the polar vs. nonpolar carotenoid contents between vitreous and opaque lines while total carotenoid contents of the vitreous and opaque lines are identical. The correlation between endosperm vitreousness and the polar vs. nonpolar carotenoid ratio is unquestionable and clearly demonstrated. Especially, knock-down of Ven1 expression in another vitreous inbred line (KN5585) induced an opaque endosperm. This work provides also interesting data and tools to investigate the carotenoid metabolism which is still poorly understood.

Response: Thanks for your professional and positive comments.

2 However, it should be pointed out that zeaxanthin synthesis takes place in the opaque lines, even if at a lower level. Is there another redundant gene (β-carotene hydroxylase) that could be involved in zeaxanthin synthesis? Using BLAST, a highly homologous gene Zm00001d002589 (hyd4-hydroxylase4) is expressed (although weakly) in the endosperm. This is not commented in the manuscript.

Response: Thanks for the comment. We have addressed this question above. There are five carotene β-ring hydroxylase genes, *HYD3*, *HYD4*, *HYD5*, *HYD6*, and *CYP97A*, and the one ε-ring hydroxylase gene, *CYP97C,* in maize (Vallabhaneni et al. 2009). Based on our RNA-seq data, the transcript level of *Ven1/HYD3* was dramatically reduced in NILA619 compared to NILW64A,

while those of *HYD4*, *HYD5*, *HYD6*, *CYP97A*, *CYP97C* were not apparently affected. This may explain that xanthophylls accumulates in NILA619.

FPKM value of maize carotene hydroxylase genes in NILW64A and NILA619.

gene_id	gene_symbol	NILA619_1	NILA619_2	NILA619_3	NILW64A_1	NILW64A_2	NILW64A_3
**Zm00001d026056**	*Ven1/HYD3*	5.4328	6.22873	5.14103	18.9842	23.7906	16.9743
**Zm00001d002589**	*HYD4*	0.479365	0.642864	0.766779	0.488093	0.628979	0.456653
**Zm00001d048469**	*HYD5*	0.022482	0	0.177048	0.020934	0	0.042379
**Zm00001d027443**	*HYD6*	0.272699	0.071853	0.874325	0.126148	0.165311	0.25856
**Zm00001d018488**	*CYP97A*	3.58885	3.24915	3.37286	2.19734	3.19095	2.23842
**Zm00001d029822**	*CYP97C*	1.01872	0.606432	1.53814	0.975302	1.27565	0.722895

3 The molecular and physicochemical causes of this effect were further explored. By different approaches including electron microscopy (TEM) and lipid analyses (UPLC/mass spectrometry), the authors conclude that these changes of the carotenoid composition impact the stability of amyloplast membranes. This conclusion is however questionable. First of all, this hypothesis is only based on TEM observations. The authors observed some discontinuities in the amyloplast membranes of the vitreous lines that are correlated with adhesion of protein bodies to starch granules. However, owing to the particular hard texture of the vitreous endosperm, fixation and sectioning could induce membrane disruption.

Response: Thanks for your comment. Based on my long-term experience in TEM of maize endosperms, we never found that the breakdown of amyloplast membranes in vitreous endosperm was induced by the fixation and sectioning. We have cut a large number of sections of maize endosperms from 18 to 35 DAP. Simply, if the membrane breakdown was caused by the fixation and

sectioning, the membranes around starch granules in all sections of NILW64A and NILA619 should be disrupted.

Secondly, to strengthen this hypothesis, no changes in protein and starch accumulation were reported between the vitreous and floury lines. The zein contents and compositions seem identical on the basis of SDS-PAGE. However, in Supplementary fig.6, only zein bands are highlighted and changes in the content of the other proteins (albumins, globulins and glutelins) cannot be determined. Furthermore, SDS-PAGE is not as sensitive as a chemical or colorimetric assay (e.g., a nitrogen assay would be compatible with the extraction buffer).

Response: Thanks for your suggestion. We have measured the total protein content. The result showed no apparent difference in total protein levels between the NILs. These data have been added in the new Supplementary fig. 6.

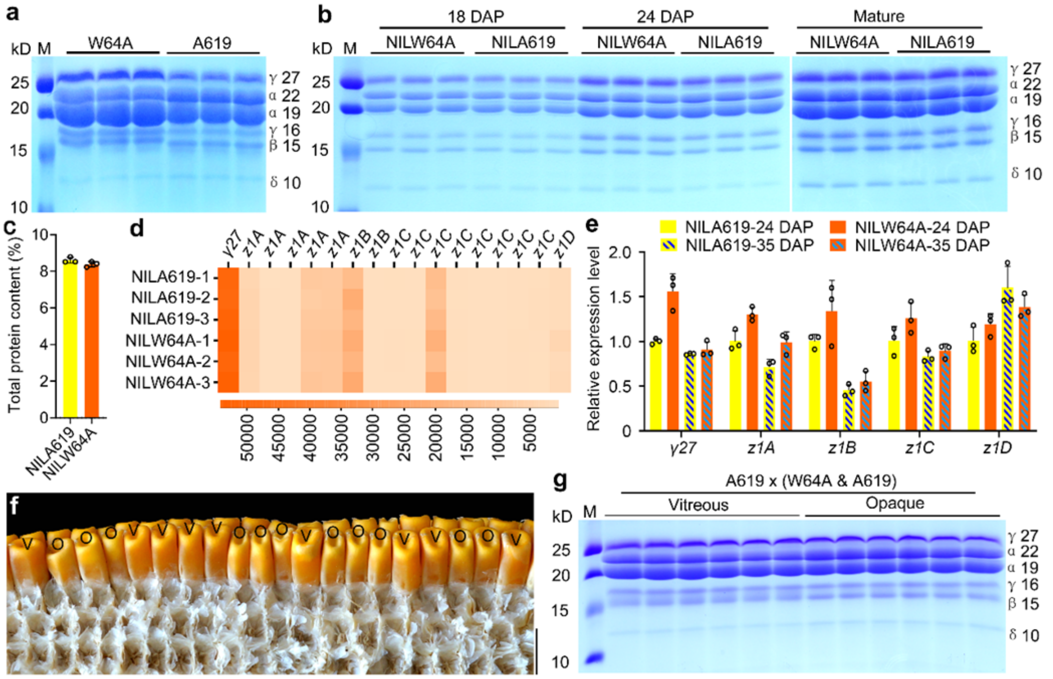

The composition of the storage carbohydrate polymers was not determined, while the ratio of the amylose to amylopectin ratio could strongly impact endosperm vitreousness. It should be noted that this ratio may vary even if

the starch content does not change. As mentioned by the authors (beginning of the discussion), the high-amylose maize is vitreous while the waxy (high amylopectin maize) are opaque. This was also observed in maize inbred lines (ref 4) where the vitreous regions of the maize endosperms have higher amylose contents than the floury ones. It is difficult to predict this ratio on the basis of the transcript level of starch biosynthetic enzymes (supplementary fig.7). Indeed, the activity of these enzymes is highly regulated by post-translational modifications (phosphorylation, formation of protein-protein complexes). Furthermore, considering that carotenoids and starch polymers are synthesized in the same subcellular compartment, i.e., the amyloplast, interference between these metabolic pathways could occur.

Finally, in regard to the suggested degradation of amyloplast membranes, the lipids were analyzed in the vitreous and opaque lines. Lipid analyses has to be performed, since carotenoids and lipids compete for the same carbon flux (and starch as well). The main drawback of these lipid analyses is the endosperm isolation. In this work, the endosperm was obtained after seed coat and embryo removal. This leaves most of the aleurone cell layer surrounding the starchy endosperm. The aleurone cells contain high amount of lipids, especially storage lipids, i.e. triacylglycerols (TAG), while most of the TAG of the starchy endosperm are degraded into free fatty acids duringendosperm development (Tan, S. L., Morrison, W. R. Distribution of lipids in the germ, endosperm, pericarp and tip cap of amylomaize, LG-11 hybrid maize and waxy maize. J. Am. Oil Chem. Soc. 1979, 56, 531−535); Rolletschek et al. Positional cues for the starch/lipidbalance in maize kernels and resource partitioning to the embryo. Plant J. 2005, 42, 69-83). The high amounts of TAG reported in fig.3 suggest that endosperm is highly contaminated by aleurone cells. Although this contamination is not a major problem for the main storage compounds, i.e., proteins and starch (aleurone cells do not accumulate zeins and starch), this is not the case for storage lipids which are about thirty times more concentrated in the aleurone layer than in the starchy endosperm. It is only possible to conclude for the free fatty acids (FA) and galactosyldiacylglycerol (DGDG, MGDG) contents, which concentrate and are specific of the starchy endosperm, respectively.

Concerning galactosyldiacylglycerol, it is surprising to observe very low amounts of MGDG (fig. 3k) while previous studies reports similar contents (Tan & Morrison, 1979 cited above; Gayral, et al. Lipid partitioning in maize (Zea mays L.) endosperm highlights relationships among starch lipids, amylose, and vitreousness. Journal of agriculturaland food chemistry, 2015, 63, 3551-3558). In this regard, it was not specified whether standards were used to quantify lipids in UPLC-mass spectrometry in the Methods section.

Furthermore, lipids were extracted using CH2Cl2-MeOH and 2-propanol, a procedurethat does not extract lipids embedded within starch granules. These lipids can be extracted by hot 1-propanol-water (see Gayral et al, 2015, cited above). Actually, starch lipids account for about 50% of total lipids of maize starchy endosperm and are composed of free fatty acids (FA) and lysophospholipids (LPC, LPE). These starch-bound lipids are a specificity of cereal starches and are related to the cell death (PCD, programmed cell death) development of cereal endosperms, which induces a specific endoplasmicreticulum (ER)-plastid trafficking. This trafficking drives MGDG and DGDG synthesis as well as the amylose to amylopectin ratio and finely is correlated to endosperm vitreousness (see Gayral et al, 2015 cited above and Gayral et al The spatiotemporal deposition of lysophosphatidylcholine within starch granules of maize endosperm and its relationships to the expression of genes involved in endoplasmic reticulum–amyloplast lipid trafficking and galactolipid synthesis. Plant Cell Physiol., 2019, 60, 139-151). It could be expected that changes in the carotenoid composition impact this lipid trafficking. Carotenoid trafficking can also occur between ER and amyloplast as observed for chloroplasts and changes in the carotenoid composition can also modify this trafficking. This can impact fatty acid desaturation in the ER as observed in this work (Mehrshahi et al. Transorganellar complementation redefines the biochemical continuity of endoplasmic reticulum and chloroplasts. Proc Natl Acad Sci U S A. 2013, 12126-12131). It is therefore essential to determine both the amylose-amylopectin ratio as well as the composition of starch lipids in the vitreous and opaque inbred lines.

Finally, the authors should consider that more than “membrane degradation” the impact of the modifications of the carotenoid biosynthetic pathway can

induce huge modifications of the endosperm metabolism. Especially, changing the carotenoid composition and probably the ER-amyloplast interactions and ER-amyloplast trafficking should amplify the ER stress and the UPR responses that drive endosperm PCD and endosperm metabolism (Shank et al. Induction of lipid metabolic enzymes during the endoplasmic reticulum stress response in plants. Plant Physiol. 2001, 126, 267-277; Gayral et al. Responses to hypoxia and endoplasmic reticulum stress discriminate the development of vitreous and floury endosperms of conventional maize (Zea mays) inbred lines. Front Plant Sci. 2017, 8:557). Increase of storage lipids (TAG) and-or their hydrolysis products (FA) in NILA619 was also observed in the opaque floury2 maize (Shank et al, 2001) or in the opaque regions of maize inbred lines (Rolletschek et al, 2005; Gayral et al, 2015) and was related to a boost of UPR (Shank et al, 2001; Gayral et al, 2017).

Response: Thanks for your suggestion. We have measured the content of total starch and amylose in the NIL endosperms, which showed no difference (Supplementary Fig. 7d). The amylose content has been added.

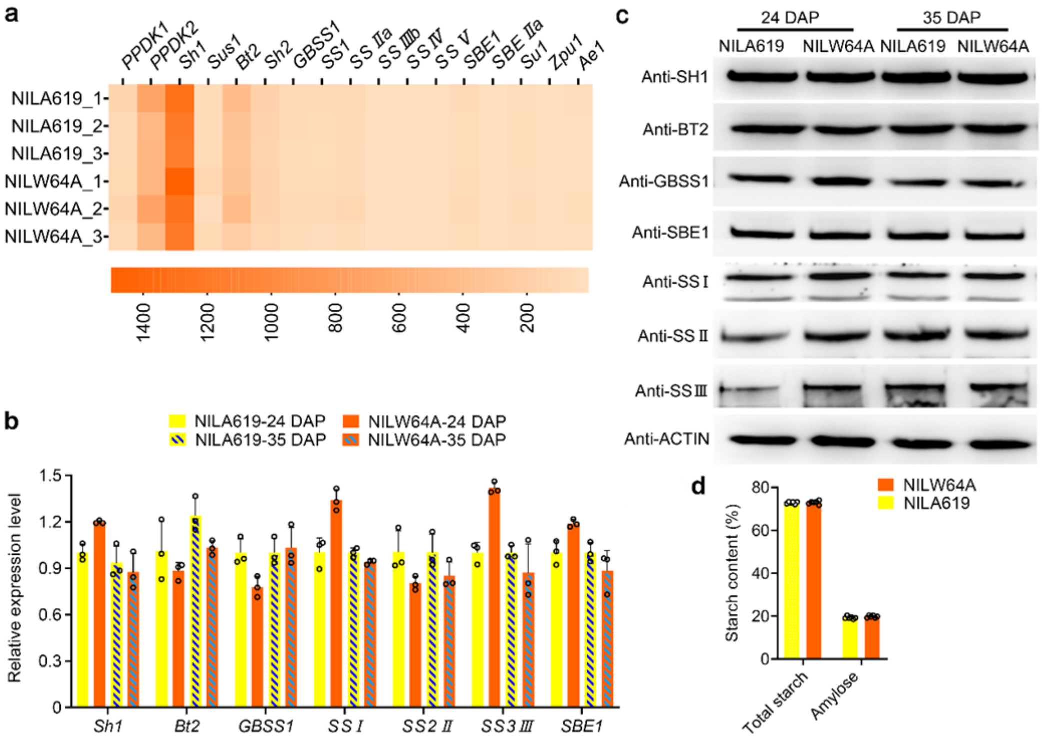

We missed some important information about the endosperm sample collection. The seed coat, embryo and aleurone were clearly separated from the starchy endosperm during the filling stage from 18-35 DAP. Tissue contamination was easily avoided. The description has been added in the Methods.

Lipid composition was according to the report (Xie et al, Ultrasound-assisted one-phase solvent extraction coupled with liquid chromatography-quadrupole time-of-flight mass spectrometry for e□cient profiling of egg yolk lipids. Food Chemistry 319 (2020) 126547). All lipid standards were the same as described in this study.

Other comments:

In NILA619 endosperm, the formation of multilamellar vesicles could support an ER-phagy process (Fig 3f) that strengthens an increase of stress responses in opaque maize (similar to autophagy) (Bernales et al (2007). ER-phagy: selective autophagy of the endoplasmic reticulum. Autophagy, 2007, 3, 285-287). Autophagy is triggered by UPR in agreement with the comments done above. These lamellar structures could trap protein bodies (Fig 3g), thus preventing fusion of PBs. The fusion of protein bodies could be a prerequisite for the adhesion of the protein bodies to the surface of the starch granules. It is questionable whether in fact the kinetics of events (UPR, autophagy) leading to cell death (contributing to PCD) would be earlier in opaque lines than in vitreous lines. This would be a common molecular mechanism induced here by changes in the carotenoid composition and by other metabolic changes in other opaque lines (or in opaque regions of dent lines). Indeed, the extent of vitreousness could be the result of a balance between the management of stress responses during endosperm development.

Response: Thanks for your comment and suggestion. Vitreous endosperm formation in maize involves all manner of things, including the synthesis of storage proteins, starch and some known and unknown components in cytoplasm. The formation of multilamellar vesicles and ER-phagy process

might affect vitreous endosperm formation. However, at the current stage, it is difficult for us to test this hypothesis without correct mutants. Since UPR and PCD also occur in wild type, if you change one thing it could change 100 other things. Our EMS mutagenesis did not reach saturation for genetic screen (otherwise it was a huge amount of field work), we couldn't precisely identify which compound was a causative factor that affects the vitreous endosperm formation in A619. Regardless of the mechanism, the *Ven1-A619* phenotype reveals an unexpected barrier for efforts directed toward increasing pro vitamin A content of maize grain. Currently, we were simply trying to offer a logical hypothesis based on the data. Maize kernel vitreousness is a complex phenotype and we were presenting new data that offers insight into how vitreous endosperm forms. The unsolved mechanism can be left for subsequent research.

In fig1c, SEM was performed on the vitreous region of the endosperm of W64A and NILW64A while SEM was performed on the opaque region of A619 and NILA619. It would have been more pertinent to compare the vitreous and opaque regions of the different maize line. Eventually SEM at the interface of the opaque-vitreous regions of each line should give more information on the relationship between carotenoid composition and packing of PBs and SG in the endosperm.

Response: Thanks for your suggestion. In Fig1c, we compared the packing of PBs and SGs in the same region of kernels of NILs. The results clearly showed the difference in vitreous and opaque endosperm of different lines.

At this stage of the data, fig 5e is quite speculative.

Response: In any case, maize kernel vitreousness is a complex phenotype and we were presenting new data that offers insight into how vitreous endosperm forms. The unsolved mechanism can be left for subsequent research.

There are many typing errors in the manuscript: About quantitative trait the loci >> About quantitative trait loci

Response: Thanks for the comment. We have checked and corrected the typos throughout the manuscript.

« . ..ranging from nearly completely vitreous to completely starchy.” Use “opaque” or “floury” instead of starchy; starchy could suggest that the endosperm contains only starch.

Response: Thanks for the suggestion. We have used “opaque” instead of “starchy”.

“Although the reason is unclear, this mutation affects monogalactosyldiacylglcerol,the enzyme responsible for synthesis of amyloplast membranephospholipids ».

Although the reason is unclear, this mutation affects monogalactosyldiacylglcerol synthase, the enzyme responsible for synthesis of amyloplast membrane galactolipids

Response: Revised.

Use membrane disruption instead of membrane degradation; degradation means that components are metabolically transformed. Here, it’s more a mechanical effect.

Response: We meant the membrane degradation here, because membrane galactolipids are transformed to other FAs or TAGs accompanying the amyloplast membrane degradation.

Reviewer #3 report:

Wang et al.

This paper presents genetic evidence implicating carotenoid metabolism in determination of endosperm texture in maize. Vitreous and non-vitreous (opaque) endosperm phenotypes, which have been studied extensively in maize, have uncovered deep connections among diverse metabolic and cellular processes that affect concretion of protein bodies and starch grains in the mature endosperm. The new results show that carotenoid pigments that accumulate in the amyloplast envelop membranes affect membrane stability during endosperm maturation.

A recessive non-vitreous factor in inbred A619 is identified as a partial deletion of a carotenoid 3-hydroxylase involved in biosynthesis of xanthophylls. Accumulation of non-polar carotenoids in the Ven1-A619 background is proposed to stabilize amyloplast envelop membranes delaying degradation and preventing contact between starch grains and protein bodies. In support of this hypothesis, several carotenoid deficient mutantsrecovered as suppressors are shown to be epistatic to the Ven1-A619 mutation. One complication is that the suppressors identified are pleiotropic with effects that are not limited to carotenoids.

Regardless of the mechanism, the Ven1 phenotype reveals an unexpected barrier for efforts directed toward increasing pro vitamin A content of maize grain.

For these reasons, the results are novel and will be of substantial interest to the plant genetics and breeding communities.

Response: Thanks for your professional and positive comments.

Specific comments

1 The suppressor screen uncovered four mutants in known genes that have carotenoid deficient phenotypes. However, all of them are in enzymes that are not specific to the carotenoid pathway affecting tocopherol biosynthesis and other isoprenoid pathways. While the screen may not have reached saturation,

there are at least a comparable number of known loci that are specific to the carotenoid pathway that would also be predicted to suppress Ven1. Is y1 (phytoene synthase) also epistatic to Ven1? In addition to being more specific to the non-polar carotenoids, y1 is the principle genetic determinant of carotenoid content in breeding lines.

Response: Thanks for your suggestion. In the revised manuscript, we have also cloned the other two suppressors. The second (also known as *seed carotenoid deficient*, *Scd*) encodes a hydroxymethylbutenyl diphosphate synthase (HDS) converting 2C-methyl-D-erytrithol 2,4-cyclodiphosphate to 1-hydroxy-2-methyl-2-(E)-butenyl 4-diphosphate, and the forth is a missense variant allelic to the third suppressor encoding DXS. According to the standard nomenclature for maize genes, the four *ven1* suppressors were renamed *vitreous endosperm suppressor 1, 2, 3-1* and *3-2* (*ves1*, *ves2*, *ves3-1* and *ves3-2*).

Because our EMS mutagenesis did not reach saturation for genetic screen (otherwise it was a huge amount of field work), we don't have *psy* mutants in A619. This year, we screened at least eight new A619 EMS mutants with a white to pale-yellow endosperm. We don't know whether a *y1* mutant was included, but all these mutants are vitreous. Although we have not cloned these genes yet, we believed that *y1* is also epistatic to *Ven1*^{A619}.

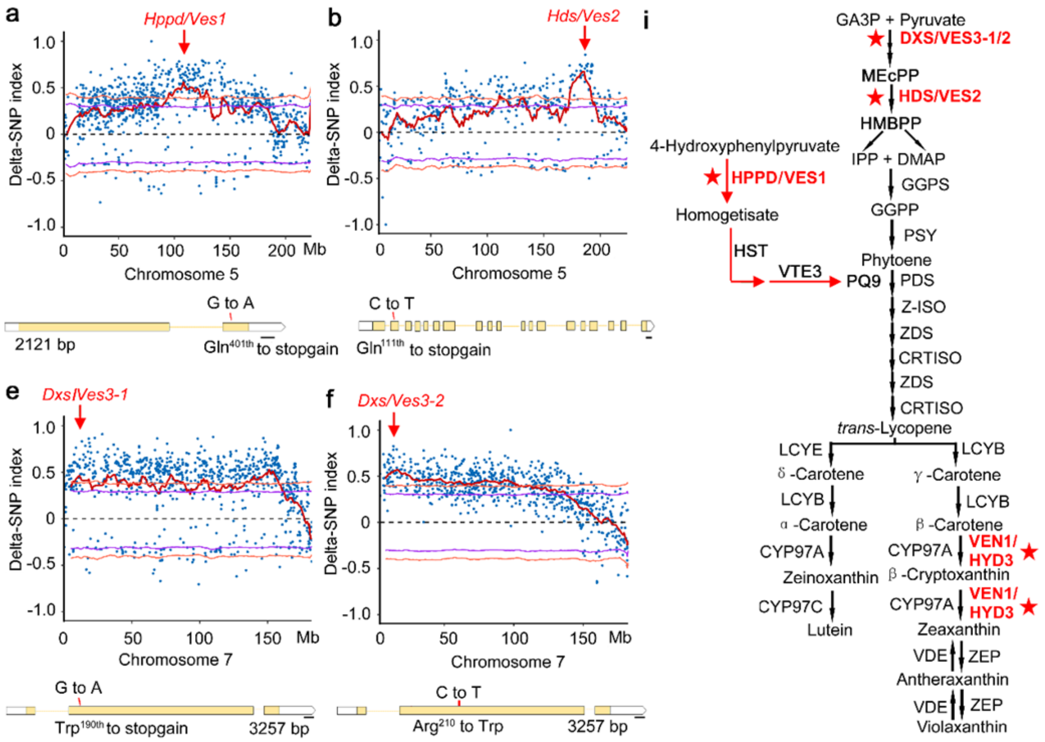

2 The evidence implicating non-polar carotenoids specifically is indirect. The authors dismiss the possibility that a product of Ven1 could cause membrane degradation on the basis that suppressors are carotenoid deficient. However, suppose for example that in vitreous endosperm membrane degradation is induced by an interaction between xanthophylls and tocopherols. In that case, Ven1-A619 would block degradation by blocking synthesis of xanthophylls whereas suppressors that reduce tocopherols obviate the need for xanthophylls. In any case, the apparent complexity of genetic background effects suggests that the Ven1-A619 phenotype requires more than accumulation of carotenoids.

Response: Thanks for your comment. When we first cloned the *Ven1* gene, we considered the possibility that a product of *Ven1* could cause membrane degradation in W64A, but it is unlikely based on the later studies. *Ves1* encodes 4-hydroxyphenylpyruvate dioxygenase 1 (HPPD), which is the first committed step in synthesis of both plastoquinone and tocopherols in plants. In *ves1* endosperms, the syntheses of tocopherols and xanthophylls are both blocked and the endosperm is vitreous, excluding the possibility that vitreous endosperm membrane degradation is induced by an interaction between xanthophylls and tocopherols. This observation suggests that tocopherols and carotenoids are not required for vitreous endosperm formation.

As you pointed out, in any case, the apparent complexity of genetic background effects suggests that the Ven1-A619 phenotype requires more than accumulation of carotenoids. Maize kernel vitreousness is a complex phenotype and we were presenting new data that offers insight into how vitreous endosperm forms. The unsolved mechanism can be left for subsequent research.

3 Regarding the sentence containing "Since protein content is usually determined by the maternal genotype and endosperm vitreousness by the filial genotype".This statement implying endosperm protein content is

generally under maternal control is made without a citation. What is the basis for this statement?

Response: Thanks for the comment. Yongrui Wu and Joachim Messing published a paper that showed the protein levels of the crosses of Illinois Protein Strains and α-zeinRNAi lines were determined by maternal genotype (Wu and Messing, PLos One, 2012). Brian A. Larkins et al. summarized the opaque mutants in a book chapter "Maize Seed Storage Proteins" (Larkins, et al., 2017). These mutants are recessive and determined by the filial genotype. The two references have been cited.

4 The nomenclature is non-standard for maize genetics. The naming of suppressors is especially confusing as these have the form of allele designations.

Response: Thanks for your suggestion. In the revised manuscript, we have also cloned the other two suppressors. The second (also known as *seed carotenoid deficient*, *Scd*) encodes a hydroxymethylbutenyl diphosphate synthase (HDS) converting 2C-methyl-D-erytrithol 2,4-cyclodiphosphate to 1-hydroxy-2-methyl-2-(E)-butenyl 4-diphosphate, and the forth is a missense variant allelic to the third suppressor encoding DXS. According to the standard nomenclature for maize genes, the four *ven1* suppressors were renamed *vitreous endosperm suppressor 1, 2, 3-1* and *3-2* (*ves1*, *ves2*, *ves3-1* and *ves3-2*).

Reviewer #1 (Remarks to the Author):

The manuscript is much improved and addresses an important biological phenomenon of economic and nutritional importance. Very minor points remain to be addressed.

1. “The carotenoid biosynthesis pathway is conserved in maize and other plants, and the candidate genes, quantitative trait loci, and phenotypic loci are well characterized 11,14-18”

-Missing is the paper that definitively shows the Z-ISO enzyme. Please add:
Beltrán et al. (2015) Control of carotenoid biosynthesis through a heme-based cis-trans isomerase. Nature Chemical Biology 11(8):598-605

2. “According to these reports, we summarized the maize carotenoid biosynthesis pathway and included the suppressors in Supplementary Fig. 10e”

-where is “Supplementary Fig. 10e pathway”? There seems to be something wrong with several of the figure labels in Supplementary Fig. 10. Also, the pathway is there but it is not labelled as 10e.

3. “Ves3 encodes the 1-deoxy-D-xylulose-5-phosphate synthase (DXS), which is in upstream of VES2, a limiting enzyme for 2-C-methyl-D-erythritol 4-phosphate (MEP) for plant plastidic isoprenoid biosynthesis (Supplementary Fig. 10e)21.”

-DXS has been characterized in maize as a gene family with specific family members important for carotenoids in maize endosperm (DXS3). Please indicate which family member (locus) is represented by the suppressor. This result builds nicely on earlier work- see:
Vallabhaneni, R., and Wurtzel, E.T. (2009) Timing and biosynthetic potential for provitamin A accumulation in maize. Plant Physiology 150 (2):562-572.

4. Discussion- “Moreover, carotenoids are precursors to hormones and many other apocarotenoids (including many new apocarotenoids recently discovered, such as β-cyclocitral derived from β-carotene, strigolactones, and others)23,32.”
-ref #23 does not seem to be correct.
-Instead of Moise reference #32, better to use ref #18
-add a ref for b-cyclocitral, since it is specifically mentioned:
Dickinson et al. PNAS May 21, 2019 116 (21) 10563-10567

5. Fig 5e. As presented in the earlier manuscript, the discussion retains the idea that carotenoids destabilize the membranes leading to the vitreous phenotype. As pointed out in the previous reviews, the revised paper does not present a direct role of carotenoids. If model is presented, the text needs to be softened to reiterate that carotenoids acting directly on the membranes is just one possible mechanism for interfering with the vitreous phenotype.

Eleanore T. Wurtzel

Reviewer #2 (Remarks to the Author):

This manuscript reports i) a correlation between carotenoid biosynthesis and endosperm vitreousness in two dent maize inbred lines and ii) the physicochemical basis of this correlation.

While the correlation was unambiguously demonstrated and led to the identification of a gene, i.e., ven1, involved in carotenoid hydroxylation, the physicochemical basis of the phenotypic effect of ven1 (β-carotene 3-hydroxylase) on endosperm vitreousness is still questionable. The absence of the

expression of this enzyme led to a significant decrease of zeaxanthin content and an expected increase of the β-carotene content, i.e., to a significant change of the ratio between polar and non-polar carotenoids, while the total carotenoid content was unaffected. Since carotenoids are located in the membranes of the plastid envelop (here the non-photosynthetic amyloplast), the authors have logically focused their investigation on the role of carotenoid composition on the physicochemical properties of amyloplast membranes. This hypothesis was also strengthened by numerous works showing that, in vitro, carotenoids modulate the physical properties of model lipid membranes. Especially, zeaxanthin, but not β-carotene, increases the rigidity of model phospholipid membranes (ref 33, 34). This effect of polar carotenoids is similar to the effect of cholesterol, i.e., limits the molecular motion of the lipid alkyl chains and favors the extended conformation of the alkyl chains of bilayer membranes.

By electron microscopy, the authors observed leakage of amyloplast membranes in the endosperm of vitreous inbred lines as early as 24 DAP, but not in the endosperm of opaque inbred lines (Fig. 3). They postulate that membrane leakage (preferred to "integrity") facilitates the interaction (collapse) between protein bodies and starch granules. Plastid membrane leakage is probably an artifact of the sectioning process, due, however, to changes of membrane rigidity. If membrane leakage happened during the development of the endosperm, then starch synthesis would stop. This does not occur since the starch content as well as the amylose-amylopectin ratio of the opaque and vitreous lines are strictly similar. At least, it can be suggested that the mechanical properties of amyloplast membranes might have been impacted by changes in the carotenoid composition. At this stage, we cannot say whether the physical properties of the membranes determine the interaction strength between starch and proteins. Especially, because vitreousness can only be observed after endosperm dehydration (glass transition), a process that induces major liquid-crystalline phase transitions of membranes at the interface between starch granule and proteins (see ref 31 and references therein).

Furthermore, comparing figures 3c-d and figures 3g-h, it could be suggested that vitreousness is also related to the rearrangement of PB membranes. Indeed, numerous liposome-like structures are observed in the interfacial spaces between PBs of vitreous endosperms while PB membranes are persistent in opaque endosperms. The lipid-mediated aggregation of PBs could be a prerequisite for the adhesion of PB

This collapse could be due to changes in the composition of membranes. Unfortunately, the lipid analyses are not sound. First, the TAG level is high and shows evidence of contamination of the starchy endosperm, at least, by aleurone cells. Indeed, the aleurone layer concentrates TAG, while the starchy endosperm concentrates FA and DAG, hydrolytic products of TAG, as previously demonstrated (Tan, S. L., Morrison, W. R. Distribution of lipids in the germ, endosperm, pericarp and tip cap of amylomaize, LG-11 hybrid maize, and waxy maize. J. Am. Oil Chem. Soc. 1979, 56, 531−553; Rolletschek et al. Positional cues for the starch/lipid balance in maize kernels and resource partitioning to the embryo. Plant J. 2005, 42, 69-83). Secondly, the phosphatidic acid level is abnormally high (ref 28 ; Gayral, et al. JAFC, 2015, 63, 3551-3558) and might be due to contamination of the hand-dissected starchy endosperms by scutellum and-or embryo (see Tan & Morrison 1979). This contamination will also artefactually increase the TAG content of the starchy endosperm fractions. Finally, the DGDG/MGDG ratio is not consistent with what has been found in many previous studies, including a previous one on the same W64A lineage (ref 28). The MGDG content is atypically low. Quantitative analyses by mass spectrometry need the use of standards. In the material and methods section, the authors refer to the work of Xie et al (ref 42) for the UPLC-MS analysis. However, Xie et al. did not report the analysis of plant galactolipids. The standards used for MS analyses of endosperm galactolipids have to be mentioned. Since galactolipids are specific of amyloplast membranes, it is essential to correctly determine the content of these lipids to better explore the relationship between carotenoid synthesis and the stability of amyloplast membranes.

Finally, at this stage, I suggest to moderate the discussion concerning the role of amyloplast membranes and modify the title, e.g. “Carotenoids modulate kernel texture in maize by influencing

the organization of endosperm membranes ". The persistence of the membrane of PBs in opaque and their collapse in the vitreous endosperm is certainly a sound and significant result of this study. It could also drive the fusion of PB and amyloplast membranes. Indeed, amyloplast membrane lipids are still present while amyloplast membranes are not observed in TEM images.

Other comments:

<i>"At 35 DAP, the amyloplast membranes in NILW64A completely disappeared, enabling PBs to physically interact with SGs (Fig. 3c)."</i>

Amyloplast membranes are not observed on TEM images but their main lipids, i.e. galactolipids are present. As observed in durum wheat (ref 31), there is probably a fusion between PB and amyloplast membranes, especially during the dehydration phase which will orientate the glass transition of the endosperm matrix towards a vitreous or an opaque solid state.

<i>"It appears the stability of the membranes creates a barrier that prevents PBs and the cytoplasmic matrix from approaching the SGs. There was little of the dense cytoplasmic contents around PBs compared to NILW64A (Fig. 3h). These results suggested Ven1A619 somehow influences the breakdown of amyloplast membranes, which is critical for allowing for SG-PB interaction."</i>

As mentioned above, the persistence of PB membranes could also prevent interaction between PBs and SGs.

<i>"FA and diacylglycerides (DAG, the minor neutral lipid constituent) were significantly higher in NILA619 than NILW64A, although TAG was not apparently different (Fig. 3j)"</i>

As previously mentioned, TAG are probably provided by contamination of aleurone cells (and also by scutellum-embryo in regard to the PA content) while FA and DAG are the major neutral lipids of starchy endosperm (see Tan & Morrison, 1979 cited above)

<i>"For both neutral and membrane polar lipids, there was a remarkable shift in their saturation levels. Polyunsaturated fatty acids were more abundant in lipids extracted from NILA619 compared with NILW64A (Supplementary Fig. 9a-b)"</i>

However, it seems that their saturation level is not shifted. To determine the saturation level, it is necessary to express in mol % of total FA and not in mol% per endosperm mass. Indeed, fig 9(a and b) reflects the difference of lipid contents between opaque and vitreous inbred lines.

<i>"A tentative explanation for the dispersal of PBs in NILA619 is the altered lipid composition of the amyloplast membranes (Fig. 3i-k and Supplementary Fig. 9)."</i>

Fig 3i-j presents total lipids (mainly composed of neutral lipids) and neutral lipids not present in amyloplast membranes. Fig 3k presents the content of polar lipids. Only MGDG and DGDG are specific of amyloplast membranes. PC is present in both amyloplast and PB membranes. It seems that the galactolipid composition is not altered, only the galactolipid content. The same comment could be done from suppl. Fig 9b. As for the FA saturation level, it is necessary to consider the mol% of total galactolipids (for example) to conclude on the alteration of lipid composition.

<i>"In wheat, dark lipid inclusions (containing glycolipids, phospholipids, free fatty acids, and monoglycerides) were found to organize in a liquid crystalline phase with PBs and SGs during vitreous endosperm formation31"</i>

No PBs are found in mature wheat endosperm; replace PB by protein matrix.

<i>"In NILW64A, coincident with amyloplast membrane degradation, the membrane lipids significantly decreased and their composition shifted to neutral lipids, TAG, and FA (Fig. 3). As a result, the residual membrane lipids might organize the FA and TAG into liposome-like structures."</i>

TAG organize in lipid droplets ("oil bodies") and cannot organize in liposome-like structures. On the contrary, FA (and also DAG) can be dispersed in lamellar structures ("liposome-like"). Lipid droplets are not observed in TEM images strengthening the fact that TAG reveals contamination from lipid-rich cells (aleurone, scutellum, embryo).

<i>"Alteration in lipid composition might affect the PB- PB and SG-PB interactions (Fig. 3i-j), although further molecular and biochemical analyses are required to address this question"</i>

Lipid analyses highlight significant differences of lipid contents of all lipid classes between vitreous and opaque endosperms, but not of lipid composition. As mentioned above, the alteration of lipid composition means a modification of the proportion of the different lipid classes. These proportions are not different between opaque and vitreous endosperms.

<i>"We measured the quantity and composition of lipids in the endosperm at 35 DAP and found they were markedly different between NILA619 and NILW64A. In the endosperm, there are mainly neutral lipids, e.g., FA and TAG. Total lipid content was significantly higher in NILA619 than NILW64A (Fig. 3i), suggesting an enhanced capacity for lipid synthesis."</i>

This enhanced capacity for lipid synthesis was already observed in the opaque mutant floury2 (Shank et al, Plant Physiol. 2001, 126, 267-277) and was related to UPR which is intense in opaque endosperms (Gayral et al. Front Plant Sci. 2017, 8:557). As mentioned by the reviewer1("An alternate hypothesis is that the increase in beta-carotene leads to some other metabolic change which is responsible"). Indeed, UPR strongly modulates cell metabolism (see the numerous reviews on this particular response to ER stress).

<i>"An influence of the amyloplast membrane on vitreous endosperm formation was reported for the opaque5 mutation28. Although the reason is unclear, this mutation affects mono-galactosyldiacylglcerol synthase, the enzyme responsible for synthesis of amyloplast membrane galactolipids. Whether or not this influences amyloplast membrane stability during kernel desiccation was not reported."</i>

In <i>o5</i> there is a huge decrease in galactolipid content (fourfold decrease). This is quite different from what it is observed for the galactolipid contents of the NILW64A and NILA619 endosperms. In <i>o5</i>, this led to fewer and smaller starch granules and abnormalities in the morphology of starch granules. Although not discussed in the Myers et al publication, the amyloplast envelops (double-membrane) display some leakages and abnormalities (see figure 6 of the corresponding publication ref 28).

<i>"On the one hand, during vitreous endosperm formation the amyloplast envelope appears to break down before PB membranes (Fig. 3b-c)."</i>

As previously mentioned, this will induce differences of starch contents between opaque and vitreous endosperms. This is not observed, strengthening the fact that this is an artifact from the sectioning process, due however to changes in the mechanical properties of amyloplast membranes. Therefore, this artifact becomes a way of showing changes in the mechanical properties of amyloplast membranes.

There are typos in the manuscript, e.g. in the "methods" section (determination of the lipid

composition) replace Kinete by Kinetex and CAN by ACN, NH4Ac by NH₄Ac

Reviewer #3 (Remarks to the Author):

The revisions address most of my concerns. Due to the complexity of the genetics underlying this phenotype several key experiments that could shed light on the central hypothesis that non-polar carotenoids are responsible are probably not feasible in a reasonable time frame. These would include introducing carotenoid specific mutants, e.g. y1 and White cap,etc. The suppressor screen is a good alternative approach, but has not reached saturation. Pushing that screen to saturation would take considerable time.
In any case, regardless of whether their hypothesis is correct the implication of isoprenoid/carotenoid pathways in stability of amyloplast membranes is interesting.

Reviewer #2 (Remarks to the Author):

The revisions adress most of my concern, especially concerning the lipid/membrane part of the manuscript. Thank you for the fruitful exchanges. This work opens very interesting perspectives to investigate maize endosperm development.

REVIEWER COMMENTS

Reviewer #1 (Remarks to the Author):

The manuscript is much improved and addresses an important biological phenomenon of economic and nutritional importance. Very minor points remain to be addressed.

1. "The carotenoid biosynthesis pathway is conserved in maize and other plants, and the candidate genes, quantitative trait loci, and phenotypic loci are well characterized 11,14-18"

-Missing is the paper that definitively shows the Z-ISO enzyme. Please add:

Beltrán et al. (2015) Control of carotenoid biosynthesis through a heme-based cis-trans isomerase. Nature Chemical Biology 11(8):598-605

Response: Thanks for your suggestion. This reference has been added in the revised manuscript.

2. "According to these reports, we summarized the maize carotenoid biosynthesis pathway and included the suppressors in Supplementary Fig. 10e"

-where is "Supplementary Fig. 10e pathway"? There seems to be something wrong with several of the figure labels in Supplementary Fig. 10. Also, the pathway is there but it is not labelled as 10e.

Response: Thanks for your suggestion. We have corrected the wrong labels in Supplementary Fig. 10 as follows:

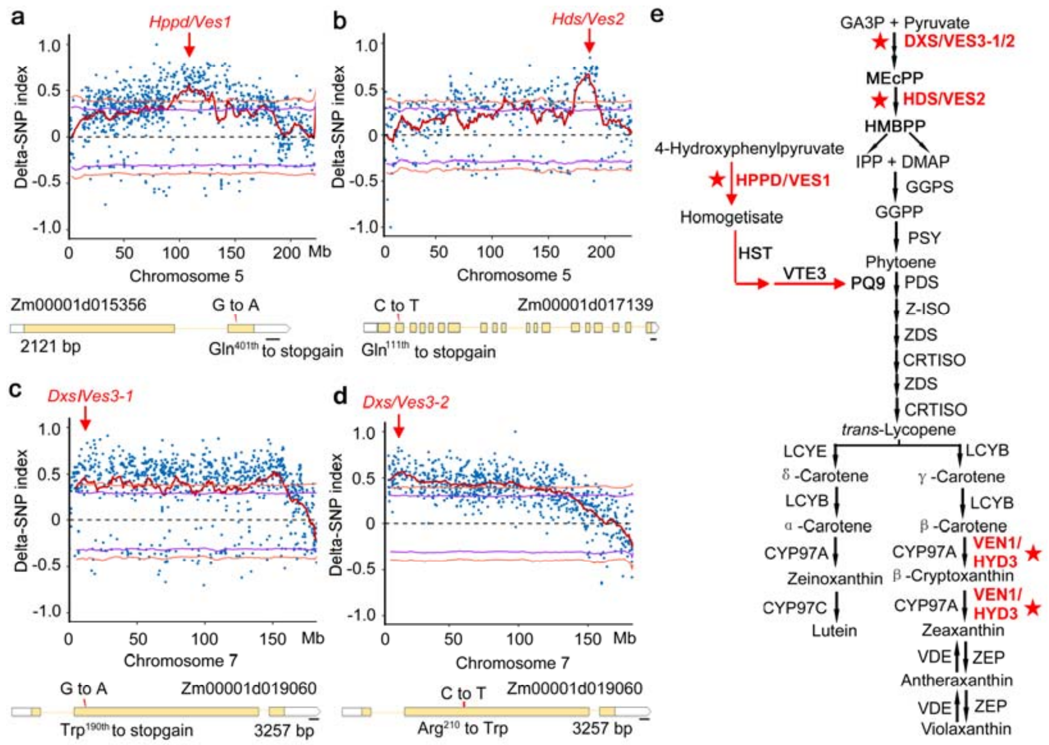

3. “Ves3 encodes the 1-deoxy-D-xylulose-5-phosphate synthase (DXS), which is in upstream of VES2, a limiting enzyme for 2-C-methyl-D-erythritol 4-phosphate (MEP) for plant plastidic isoprenoid biosynthesis (Supplementary Fig. 10e)21.”

-DXS has been characterized in maize as a gene family with specific family members important for carotenoids in maize endosperm (DXS3). Please indicate which family member (locus) is represented by the suppressor. This result builds nicely on earlier work- see:
Vallabhaneni, R., and Wurtzel, E.T. (2009) Timing and biosynthetic potential for provitamin A accumulation in maize. Plant Physiology 150 (2):562-572.

Response: Thanks for your comment. We have marked the gene id of all suppressors in Supplementary Fig. 10. Ves3-1 and Ves3-2 are the DXS2 according to your suggested paper, which has been cited in the revised manuscript.

4. Discussion- “Moreover, carotenoids are precursors to hormones and many other apocarotenoids (including many new apocarotenoids recently discovered, such as β-cyclocitral derived from β-carotene, strigolactones, and others)23,32.”
-ref #23 does not seem to be correct.
-Instead of Moise reference #32, better to use ref #18
-add a ref for b-cyclocitral, since it is specifically mentioned:
Dickinson et al. PNAS May 21, 2019 116 (21) 10563-10567

Response: Thanks for your suggestion. We have revised this according to your suggestion.

5. Fig 5e. As presented in the earlier manuscript, the discussion retains the idea that carotenoids destabilize the membranes leading to the vitreous phenotype. As pointed out in the previous reviews, the revised paper does not present a direct role of carotenoids. If model is presented, the text needs to be softened to reiterate that carotenoids acting directly on the membranes is just one possible mechanism for interfering with the vitreous phenotype.

Response: Thanks for your comment. We have softened the text at the beginning of the last paragraph as follows:
“In any case, maize kernel vitreousness is a complex phenotype and we presented new data that offers insight into how vitreous endosperm forms. Figure 5e shows a model illustrating one possible mechanism for interfering with the vitreous phenotype through carotenoids acting on amyloplast membranes.”

Eleanore T. Wurtzel

Reviewer #2 (Remarks to the Author):

This manuscript reports i) a correlation between carotenoid biosynthesis and endosperm vitreousness in two dent maize inbred lines and ii) the physicochemical basis of this correlation.

While the correlation was unambiguously demonstrated and led to the identification of a gene, i.e., ven1, involved in carotenoid hydroxylation, the physicochemical basis of the phenotypic effect of ven1 (β-carotene 3-hydroxylase) on endosperm vitreousness is still questionable.
Response: Thanks for your comment. In any case, maize kernel vitreousness is a complex phenotype and we presented new data that offers insight into how vitreous endosperm forms. Although the physicochemical basis of the phenotypic effect of *ven1* on endosperm vitreousness is not fully understood in this study, the implication of isoprenoid/carotenoid pathways in stability of amyloplast membranes will create an interesting area for investigation in the future.

The absence of the expression of this enzyme led to a significant decrease of zeaxanthin content and an expected increase of the β-carotene content, i.e., to a significant change of the ratio between polar and non-polar carotenoids, while the total carotenoid content was unaffected. Since carotenoids are located in the membranes of the plastid envelop (here the non-photosynthetic amyloplast), the authors have logically focused their investigation on the role of carotenoid composition on the physicochemical properties of amyloplast membranes. This hypothesis was also strengthened by numerous works showing that, in vitro, carotenoids modulate the physical properties of model lipid membranes. Especially, zeaxanthin, but not β-carotene, increases the rigidity of model phospholipid membranes (ref 33, 34). This effect of polar carotenoids is similar to the effect of cholesterol, i.e., limits the molecular motion of the lipid alkyl chains and favors the extended conformation of the alkyl chains of bilayer membranes.

Response: Thanks for your comment. We have revised the discussion as follows:

"Since carotenoids are located in the membranes of the plastid envelop (here the non-photosynthetic amyloplast), it was logical to hypothesize a connection between the role of carotenoid composition and the physicochemical properties of amyloplast membranes. This hypothesis was strengthened by numerous works showing that, in vitro, carotenoids modulate the physical properties of model lipid membranes. For example, β-carotene tends to be randomly distributed within the hydrophobic interior of the bilayer envelope and increase the membrane fluidity, whereas polar carotenoids span lipid bilayer and have their polar groups anchored in the opposite polar zones of membrane; as a result, they increase the viscosity of the membrane [35-37]. In addition, zeaxanthin, but not β-carotene, increases the rigidity of model phospholipid membranes[35,36]. This effect of polar carotenoids is similar to the effect of

cholesterol, i.e., limits the molecular motion of the lipid alkyl chains and favors the extended conformation of the alkyl chains of bilayer membranes. Probably, the elevated nonpolar carotenoids, particularly β-carotene, and decreased polar carotenoids, particularly zeaxanthin, created by *Ven1*^{A619} could increase the fluidity and hydrophobicity of amyloplast membranes and result in the irregularly expanded amyloplast membranes (Fig. 3e-f)."

In addition, we have softened the text at the beginning of the last paragraph as follows:

"In any case, maize kernel vitreousness is a complex phenotype and we presented new data that offers insight into how vitreous endosperm forms. Figure 5e shows a model illustrating one possible mechanism for interfering with the vitreous phenotype through carotenoids acting on amyloplast membranes."

By electron microscopy, the authors observed leakage of amyloplast membranes in the endosperm of vitreous inbred lines as early as 24 DAP, but not in the endosperm of opaque inbred lines (Fig. 3). They postulate that membrane leakage (preferred to "integrity") facilitates the interaction (collapse) between protein bodies and starch granules. Plastid membrane leakage is probably an artifact of the sectioning process, due, however, to changes of membrane rigidity. If membrane leakage happened during the development of the endosperm, then starch synthesis would stop. This does not occur since the starch content as well as the amylose-amylopectin ratio of the opaque and vitreous lines are strictly similar. At least, it can be suggested that the mechanical properties of amyloplast membranes might have been impacted by changes in the carotenoid composition. At this stage, we cannot say whether the physical properties of the membranes determine the interaction strength between starch and proteins. Especially, because vitreousness can only be observed after endosperm dehydration (glass transition), a process that induces major liquid-crystalline phase transitions of membranes at the interface between starch granule and proteins (see ref 31 and references therein).

Response: Thanks for your comment. Based on our many years' experience in this field and the data presented, it is impossible that the breakdown of amyloplast membranes in NIL-W64A is an artifact of the sectioning process.

First, if it were that case, we should have observed the membrane breakdown in NIL-W64A at 18 DAP (however, we didn't). Although vitreousness can only be observed after endosperm dehydration, it has been determined much earlier during endosperm development. Many opaque mutants provide evidence for this, e.g., *o2*, *fl2* and *Mc1*. We can predict that most mutants, if not all, are vitreous or opaque by observing the PBs and SGs during the filling stage. The size, number and organization of PBs affect SG-PB interactions. PBs must stay in positions surrounding SGs around the time amyloplast membranes are about to degrade. Timing could be critical to achieve an optimal spatial arrangement of SGs and PBs, otherwise once the starchy endosperm tissue is dehydrated, PBs are unable to approach SGs and

condense around them. This hypothesis is supported by the observation in Fig. 3g and Supplementary Fig. 8k.

Second, SGs in endosperm cells differ in maturity. At 18 DAP, most SGs, if not all, are immature and have intact membranes in NIL-W64A. At 24 DAP, many SGs in NIL-W64A were reaching maturity and their amyloplast membranes began to break down. At this stage, we could find starch grains at different extents of maturity. As shown in the below, SGs with membranes in integrity, in partial and complete degradation (the debris is present and absent in their periphery) were observed. This observation could explain that the starch content as well as the amylose-amylopectin ratio in NIL-W64A and NIL-A619 are similar. This also exclude the possibility that the breakdown of amyloplast membranes in NIL-W64A is an artifact of the sectioning process, otherwise the broken pieces of membranes should not disappear. The A and C figures in the following have been presented as Supplementary Fig. 9a. In the corresponding text, it was described as follows:
"Because SGs at this stage differed in maturity, those with membranes in integrity or complete degradation (in the latter, the debris was absent in their periphery) were also observed (Supplementary Fig. 9a)."

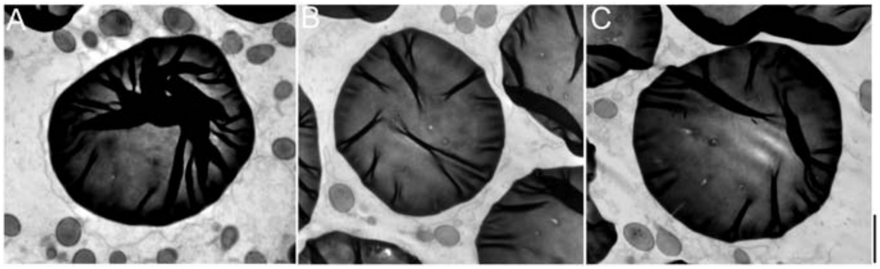

Third, the tissue fixation was performed before sectioning. As shown in Fig. 3c and 3g, if the breakdown of amyloplast membranes were an artifact of the sectioning process, the average distance between PBs and SGs should be the similar in NILA619 and NILW64A. Instead, we observed that PBs and SGs in NILW64A had a tight interaction. This close interaction could only occur before the sectioning process. In contrast, it was clear that PBs in NILA619 were precluded from contacting SGs by the amyloplast membranes.

Furthermore, comparing figures 3c-d and figures 3g-h, it could be suggested that vitreousness is also related to the rearrangement of PB membranes. Indeed, numerous liposome-like structures are observed in the interfacial spaces between PBs of vitreous endosperms while PB membranes are persistent in opaque endosperms. The lipid-mediated aggregation of PBs could be a prerequisite for the adhesion of PB.

Response: Good suggestion! We have added this in the discussion as follows: "Condensation of liposome-like structures between neighboring PBs is evident in NILW64A, but not NILA619 (Fig. 3c-d and g-h, indicated by asterisks), supporting this

hypothesis. Vitreous endosperm formation might be related to the rearrangement of PB membranes and the lipid-mediated aggregation of PBs could be a prerequisite for the adhesion of PBs."

This collapse could be due to changes in the composition of membranes. Unfortunately, the lipid analyses are not sound. First, the TAG level is high and shows evidence of contamination of the starchy endosperm, at least, by aleurone cells. Indeed, the aleurone layer concentrates TAG, while the starchy endosperm concentrates FA and DAG, hydrolytic products of TAG, as previously demonstrated (Tan, S. L., Morrison, W. R. Distribution of lipids in the germ, endosperm, pericarp and tip cap of amylomaize, LG-11 hybrid maize, and waxy maize. J. Am. Oil Chem. Soc. 1979, 56, 531−553; Rolletschek et al. Positional cues for the starch/lipid balance in maize kernels and resource partitioning to the embryo. Plant J. 2005, 42, 69-83). Secondly, the phosphatidic acid level is abnormally high (ref 28 ; Gayral, et al. JAFC, 2015, 63, 3551-3558) and might be due to contamination of the hand-dissected starchy endosperms by scutellum and-or embryo (see Tan & Morrison 1979). This contamination will also artefactually increase the TAG content of the starchy endosperm fractions. Finally, the DGDG/MGDG ratio is not consistent with what has been found in many previous studies, including a previous one on the same W64A lineage (ref 28). The MGDG content is atypically low. Quantitative analyses by mass spectrometry need the use of standards. In the material and methods section, the authors refer to the work of Xie et al (ref 42) for the UPLC-MS analysis. However, Xie et al. did not report the analysis of plant galactolipids. The standards used for MS analyses of endosperm galactolipids have to be mentioned. Since galactolipids are specific of amyloplast membranes, it is essential to correctly determine the content of these lipids to better explore the relationship between carotenoid synthesis and the stability of amyloplast membranes.

Response: Thanks for your comment. In fact, Tan and Morrison' data (see the table below. The front is too small, you can drag the table to a larger size or go to the original paper) showed that nonstarch lipids contains FA, DAG and TAG. The TAG content is higher than DAG in the three kinds of maize lines, which is consistent with our data (Fig. 3l). In addition, FA, DAG and TAG contents were largely different between these lines (Tan and Morrison 1979).

TABLE II

Composition of Lipids in Amylomaize, LG-11 Maize and Waxy Maize Kernels (mg Lipid/100 g Dry Wt.)

Amylomaize

Lipid	Endosperm: Nonstarch + aleurone	Endosperm: Nonstarch - aleurone	Endosperm: Aleurone (by difference)	Endosperm: Starch	Pure starch	Germ	Pericarp	Tip cap
Steryl ester	53	24	29	5	9	450	59	73
Triglyceride	898	92	806	14	16	32854	115	1308
Diglyceride	64	45	19	4	16	2013	31	76
Free fatty acid	657	471	186	447	650	694	38	182
Monoglyceride	19^a	10^a	9^a	9	13	169^a	7^a	27^a
Estd. steryl glycoside	15	13	7	8	13	258	14	29
Monogalactosyl monoglyceride	{11	{19	{−8	—	—	{173	{6	15
Monogalactosyl monoglyceride				22	18			10
Digalactosyl diglyceride	10	9	1	—	—	129	7	21
Digalactosyl monoglyceride	13	24	−11	34	17	213	10	16
N-acyl phosphatidyl ethanolamine	trace			—	—	73	5	10
N-acyl lysophosphatidyl ethanolamine	{2	2		—	—	—	—	trace
Diphosphatidyl glycerol				—	—			
Phosphatidyl glycerol	6		6	—	—	19	{2	8
Phosphatidyl ethanolamine	8		8	—	—	174		8
Phosphatidyl choline	31	8	23	—	—	336	7	29
Phosphatidyl inositol	9	2	7	—	—	229	3	19
Phosphatidic acid	12	3	9	—	—	333	5	14
Lysophosphatidyl glycerol				4	7			
Lysophosphatidyl ethanolamine	4^b		4^b	17	16	11^b	1^b	3^b
Lysophosphatidyl choline	29	16	13	166	183	9	7	16
Lysophosphatidyl inositol				4	6			16^c
Nonpolar lipids	1691	642	1049	479	704	36180	250	1666
Glycolipids	49	65	−16	64	48	773	37	91
Phospholipids	101	31	70	191	212	1224	30	123
Unsaponifiable lipids	47	21	26	29	67	9095	2143	1036
Total lipids	1888	759	1129	763	1031	47272	2460	2916

LG-11 Maize

Lipid	Endosperm: Nonstarch + aleurone	Endosperm: Nonstarch - aleurone	Endosperm: Starch	Pure starch	Germ	Pericarp	Tip cap
Steryl ester	49	14	4	3	818	120	253
Triglyceride	344	45	8	5	36953	163	1184
Diglyceride	33	10	3	3	1244	26	89
Free fatty acid	133	168	290	380	305	7	345
Monoglyceride	7^a	5^a	11	12	39^a	4^a	53^a
Estd. steryl glycoside	7	13	6	7	73	7	48
Monogalactosyl monoglyceride	{12		{11		{128	9	26
Monogalactosyl monoglyceride						7	25
Digalactosyl diglyceride	12		9		436	2	56
Digalactosyl monoglyceride	12		8		173	6	22
N-acyl phosphatidyl ethanolamine	3		—	—	110	11	15
N-acyl lysophosphatidyl ethanolamine	{1		—	—	{18	{3	
Diphosphatidyl glycerol			—	—			
Phosphatidyl glycerol	1		—	—	36	{4	
Phosphatidyl ethanolamine	1		—	—	194		27
Phosphatidyl choline	3		—	—	748	8	41
Phosphatidyl inositol	3		—	—	247	7	32
Phosphatidic acid	1		—	—	77	2	24
Lysophosphatidyl glycerol			4	6		{3^b	
Lysophosphatidyl ethanolamine			16^b	17^b			
Lysophosphatidyl choline	17		220	226	23	4	28
Lysophosphatidyl inositol			8^c	8^c			18^c
Nonpolar lipids	566	242	316	403	39359	320	1924
Glycolipids	43	38	34	23	809	27	177
Phospholipids	30	36	248	257	1453	42	185
Unsaponifiable lipids	72	32	—	—	1916	451	305
Total lipids	711	348	598	683	43537	840	2591

Waxy maize

Lipid	Endosperm: Nonstarch + aleurone	Endosperm: Nonstarch - aleurone	Endosperm: Starch	Pure starch	Germ	Pericarp	Tip cap
Steryl ester	32	4	3	7	382	45	104
Triglyceride	269	32	33	12	32992	102	1197
Diglyceride	11	4	3	6	542	14	178
Free fatty acid	397	384	30	105	237	59	400
Monoglyceride	8^a	2^a	1	5	123^a	9^a	35^a
Estd. steryl glycoside	11	5	1	3	404	11	76
Monogalactosyl monoglyceride		{4	{1	{1	{178		{25
Monogalactosyl monoglyceride							
Digalactosyl diglyceride		2	1	2	158		32
Digalactosyl monoglyceride		5	2	3	298		29
N-acyl phosphatidyl ethanolamine	1	1	—	—	117	5	
N-acyl lysophosphatidyl ethanolamine	{1	{1	—	—	{30	{1	
Diphosphatidyl glycerol			—	—			
Phosphatidyl glycerol	trace	trace	—	—	27	2	{3
Phosphatidyl ethanolamine	1	1	—	—	49	1	
Phosphatidyl choline	1	1	—	—	578	5	7
Phosphatidyl inositol			—	—	397	2	15
Phosphatidic acid	trace	trace	—	—	59	7	17
Lysophosphatidyl glycerol			1	trace			
Lysophosphatidyl ethanolamine			1	1	18^b	1^b	
Lysophosphatidyl choline	1	1	3	8	60	3	10
Lysophosphatidyl inositol				trace	28^c		
Nonpolar lipids	717	426	70	135	34276	229	1914
Glycolipids	18	16	5	9	1038	11	162
Phospholipids	5	5	5	9	1363	27	52
Unsaponifiable lipids	86	55	8	18	2550	936	407
Total lipids	826	502	88	171	39227	1203	2535

[a]Includes 6-0-acyl monogalactosyl diglyceride.
[b]Includes phosphatidyl serine.
[c]Includes lysophosphatidyl serine.

It is generally known that the starchy endosperm tissue is easily separated from the aleurone layer and embryo at 35 DAP. Based on our long-term experience in this field, we could maximally avoid the contamination. Even if the contamination occurred, the effect should be negligible, due to the single cell layer of aleurone and preponderance in starchy endosperm mass.

DGDG/MGDG ratio is higher compared to the measurement in previous studies, such as ref 28, probably because the different developmental stage and genetic background of the materials influenced the results. Our endosperm tissues were in A619 background at 35 DAP, whereas those in ref28 were in W64A background at 20 DAP. We have carefully checked the protocol and didn't find any problem in our lipid assay. We then quantified the lipid content of NILs at 24 DAP using the same procedure and protocol. The result didn't show a dramatic experimental variation compared to the data at 35 DAP. Instead, the alteration and difference in lipid composition between NILW64A and NILA619 at 24 and 35 DAP is consistent with the differential status of amyloplast envelope integrity.

As you pointed out, we missed the description of the standards of galactolipids for MS analyses. Since deuterium substitutes for glycolipids and odd-numbered carbon substitutes are not available on the market, we used PE to quantify glycolipids. The description for this has been added in the material and methods. The alteration and content of galactolipids in the endosperm of NILs indeed reflected the stability of amyloplast membranes in NILs from 24 DAP to 35 DAP.

The lipid result part has been significantly rephrased in the revised manuscript as follows:

"Isolated amyloplast membranes contain about 70% galactolipid and 25% phospholipid (PL), but no neutral lipids like triacylglycerides (TAG) and fatty acids (FA)[13]. Monogalactosyldiacylglycerol (MGDG) and digalactosyldiacylglycerol (DGDG) provide both structural and functional organization of amyloplast. We measured the quantity and composition of lipids in the endosperm at 24 and 35 DAP and found they were markedly different between NILA619 and NILW64A (Fig. 3i-k; Supplementary Fig. 9b-e). In the endosperm, there are mainly neutral lipids, e.g., FA and TAG. In each NIL, total lipid content was not apparently altered at the two time points, but it was always significantly higher in NILA619 than NILW64A (Fig. 3i), suggesting an enhanced capacity for lipid synthesis. At 24 DAP, when amyloplast membranes of many SGs began to break down in NILW64A, only a few polar lipids (PA, LPC and LPE) accumulated at a mildly higher level in NILA619 than NILW64A (Fig. 3j and k). This suggested that although the breakdown of amyloplast membranes occurred in NILW64A, their main lipids, i.e. galactolipids were still present in the endosperm cells. The apparent difference between the two NILs were the levels of FA and TAG. By 35 DAP, when amyloplast membranes of most SGs completely degraded in NILW64A,

the level of DGDG, which is a predominant galactolipid in amyloplast membranes, was significantly higher in NILA619 than NILW64A, as well as PL (PC, PE, PG) and lysoPL (LPC, LPE, LPG) (Fig. 5k, Supplementary Fig. 9b). It was evident that the contents of amyloplast lipids MGDG, DGDG and other membrane phospholipids (PA, PC, PE and PG) were reduced at 35 DAP compared to those at 24 DAP in the two NILs. At this stage, FA and diacylglycerides (DAG, the minor neutral lipid constituent) were significantly higher in NILA619 than NILW64A, although TAG was not apparently different (Supplementary Fig. 9c and e). The difference in lipid composition between NILW64A and NILA619 is consistent with the differential status of amyloplast envelope integrity (Fig. 3b, c ,f and g; Supplementary Fig. 9a) ."

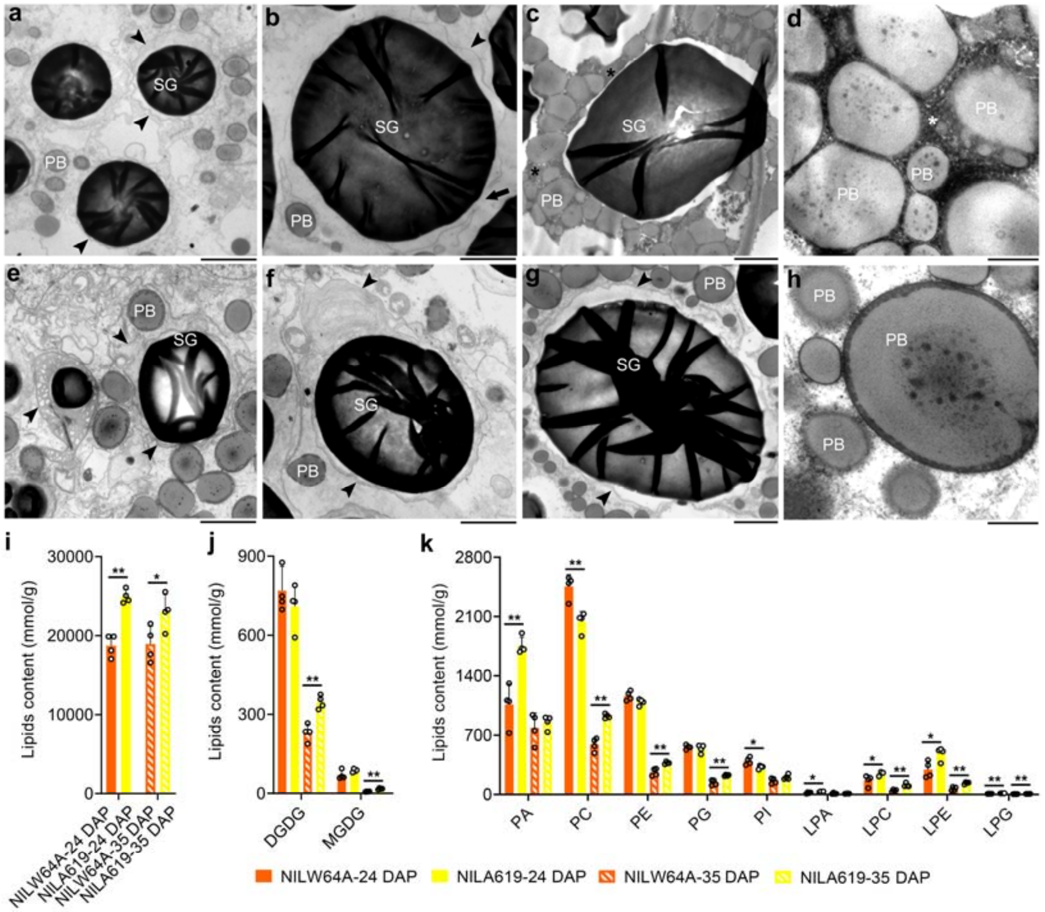

Fig. 3 Stability of PB membrane and the changes in lipid composition in endosperm of NILs.

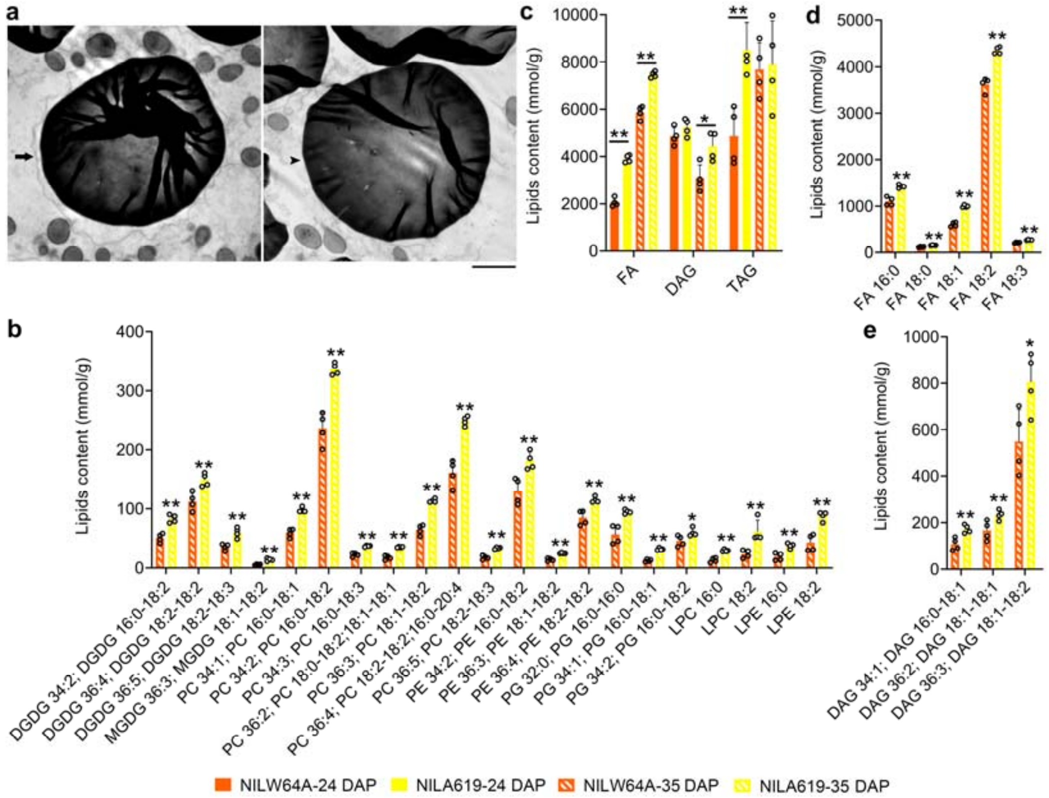

Supplementary Fig. 9 Analysis of lipid content in endosperm of NILs.

Finally, at this stage, I suggest to moderate the discussion concerning the role of amyloplast membranes and modify the title, e.g. "Carotenoids modulate kernel texture in maize by influencing the organization of endosperm membranes ". The persistence of the membrane of PBs in opaque and their collapse in the vitreous endosperm is certainly a sound and significant result of this study. It could also drive the fusion of PB and amyloplast membranes. Indeed, amyloplast membrane lipids are still present while amyloplast membranes are not observed in TEM images.

Response: Thanks for your suggestion! Because we focused the investigation of the role of carotenoid composition on the physicochemical properties of amyloplast membranes and as a consequence the vitreous endosperm formation, we preferred to use the current title.
We agree with you that the fusion of PBs and amyloplast membrane lipids probably function in vitreous endosperm formation. We have added the discussion as follows: "Condensation of liposome-like structures between neighboring PBs is evident in NILW64A, but not NILA619 (Fig. 3c-d and g-h, indicated by asterisks), supporting this hypothesis. Vitreous endosperm formation might be related to the rearrangement of PB membranes and the lipid-mediated aggregation of PBs could be a prerequisite for the adhesion of PBs."

Other comments:

“At 35 DAP, the amyloplast membranes in NILW64A completely disappeared, enabling PBs to physically interact with SGs (Fig. 3c).”

Amyloplast membranes are not observed on TEM images but their main lipids, i.e. galactolipids are present. As observed in durum wheat (ref 31), there is probably a fusion between PB and amyloplast membranes, especially during the dehydration phase which will orientate the glass transition of the endosperm matrix towards a vitreous or an opaque solid state.

Response: Thanks for your comment. We have added the following sentences in the result and discussion, respectively:

“This suggested that although the breakdown of amyloplast membranes occurred in NILW64A, their main lipids, i.e. galactolipids were still present in endosperm cells.”

“As observed in durum wheat[33], there might be a fusion between PB and amyloplast-derived membranes, especially during the dehydration phase, which will orientate the glass transition of the endosperm matrix towards a vitreous or an opaque solid state”.

“It appears the stability of the membranes creates a barrier that prevents PBs and the cytoplasmic matrix from approaching the SGs. There was little of the dense cytoplasmic contents around PBs compared to NILW64A (Fig. 3h). These results suggested Ven1A619 somehow influences the breakdown of amyloplast membranes, which is critical for allowing for SG-PB interaction.”

As mentioned above, the persistence of PB membranes could also prevent interaction between PBs and SGs.

Response: Thanks for your comments. We have added a relative discussion in another place as follows:

“Condensation of liposome-like structures between neighboring PBs is evident in NILW64A, but not NILA619 (Fig. 3c-d and g-h, indicated by asterisks), supporting this hypothesis. Vitreous endosperm formation might be related to the rearrangement of PB membranes and the lipid-mediated aggregation of PBs could be a prerequisite for the adhesion of PBs.”

“FA and diacylglycerides (DAG, the minor neutral lipid constituent) were significantly higher in NILA619 than NILW64A, although TAG was not apparently different (Fig. 3j)”

As previously mentioned, TAG are probably provided by contamination of aleurone cells (and also by scutellum-embryo in regard to the PA content) while FA and DAG are the major neutral lipids of starchy endosperm (see Tan & Morrison, 1979 cited above)

Response: Thanks for your comments. We have addressed the question above.

"For both neutral and membrane polar lipids, there was a remarkable shift in their saturation levels. Polyunsaturated fatty acids were more abundant in lipids extracted from NILA619 compared with NILW64A (Supplementary Fig. 9a-b)"

However, it seems that their saturation level is not shifted. To determine the saturation level, it is necessary to express in mol % of total FA and not in mol% per endosperm mass. Indeed, fig 9(a and b) reflects the difference of lipid contents between opaque and vitreous inbred lines.

Response: Thanks for your suggestion. The lipid result part has been significantly rephrased in the revised manuscript. This sentence has been deleted.

"A tentative explanation for the dispersal of PBs in NILA619 is the altered lipid composition of the amyloplast membranes (Fig. 3i-k and Supplementary Fig. 9)."

Fig 3i-j presents total lipids (mainly composed of neutral lipids) and neutral lipids not present in amyloplast membranes. Fig 3k presents the content of polar lipids. Only MGDG and DGDG are specific of amyloplast membranes. PC is present in both amyloplast and PB membranes. It seems that the galactolipid composition is not altered, only the galactolipid content. The same comment could be done from suppl. Fig 9b. As for the FA saturation level, it is necessary to consider the mol% of total galactolipids (for example) to conclude on the alteration of lipid composition.

Response: Thanks for your comments. This is an inappropriate statement, we revised this sentence as "A tentative explanation for the dispersal of PBs in NILA619 is the altered lipid content of the amyloplast membranes (Fig. 3i-k and Supplementary Fig. 9)."

"In wheat, dark lipid inclusions (containing glycolipids, phospholipids, free fatty acids, and monoglycerides) were found to organize in a liquid crystalline phase with PBs and SGs during vitreous endosperm formation31"

No PBs are found in mature wheat endosperm; replace PB by protein matrix.

Response: Thanks! It has been replaced.

"In NILW64A, coincident with amyloplast membrane degradation, the membrane lipids significantly decreased and their composition shifted to neutral lipids, TAG, and FA (Fig. 3). As a result, the residual membrane lipids might organize the FA and TAG into liposome-like structures."

TAG organize in lipid droplets ("oil bodies") and cannot organize in liposome-like structures. On the contrary, FA (and also DAG) can be dispersed in lamellar structures ("liposome-like"). Lipid droplets are not observed in TEM images strengthening the fact that TAG reveals contamination from lipid-rich cells (aleurone, scutellum, embryo).

Response: Good suggestion. The statements were revised as "In NILW64A, coincident with amyloplast membrane degradation, the membrane lipids significantly decreased (Fig. 3). As a result, the residual membrane lipids might organize the FA and DAG into liposome-like structures."

"Alteration in lipid composition might affect the PB- PB and SG-PB interactions (Fig. 3i-j), although further molecular and biochemical analyses are required to address this question"

Lipid analyses highlight significant differences of lipid contents of all lipid classes between vitreous and opaque endosperms, but not of lipid composition. As mentioned above, the alteration of lipid composition means a modification of the proportion of the different lipid classes. These proportions are not different between opaque and vitreous endosperms.

Response: Thanks for your suggestion. "composition" in this sentence was replaced as "content".

"We measured the quantity and composition of lipids in the endosperm at 35 DAP and found they were markedly different between NILA619 and NILW64A. In the endosperm, there are mainly neutral lipids, e.g., FA and TAG. Total lipid content was significantly higher in NILA619 than NILW64A (Fig. 3i), suggesting an enhanced capacity for lipid synthesis."

This enhanced capacity for lipid synthesis was already observed in the opaque mutant floury2 (Shank et al, Plant Physiol. 2001, 126, 267-277) and was related to UPR which is intense in opaque endosperms (Gayral et al. Front Plant Sci. 2017, 8:557). As mentioned by the reviewer1("An alternate hypothesis is that the increase in beta-carotene leads to some other metabolic change which is responsible"). Indeed, UPR strongly modulates cell metabolism (see the numerous reviews on this particular response to ER stress).

Response: This is a good idea that UPR may modulates the lipid synthesis. We also believe that UPR is important for endosperm formation. We will consider this in our future study.

"An influence of the amyloplast membrane on vitreous endosperm formation was reported for the opaque5 mutation28. Although the reason is unclear, this mutation

affects mono- galactosyldiacylglcerol synthase, the enzyme responsible for synthesis of amyloplast membrane galactolipids. Whether or not this influences amyloplast membrane stability during kernel desiccation was not reported."

In *o5* there is a huge decrease in galactolipid content (fourfold decrease). This is quite different from what it is observed for the galactolipid contents of the NILW64A and NILA619 endosperms. In *o5*, this led to fewer and smaller starch granules and abnormalities in the morphology of starch granules. Although not discussed in the Myers et al publication, the amyloplast envelops (double-membrane) display some leakages and abnormalities (see figure 6 of the corresponding publication ref 28).

Response: Thanks for your suggestion. We have rephrased the sentence as follows: "Whether or not this influences amyloplast membrane stability during kernel desiccation was not investigated in detail"

"On the one hand, during vitreous endosperm formation the amyloplast envelope appears to break down before PB membranes (Fig. 3b-c)."

As previously mentioned, this will induce differences of starch contents between opaque and vitreous endosperms. This is not observed, strengthening the fact that this is an artifact from the sectioning process, due however to changes in the mechanical properties of amyloplast membranes. Therefore, this artifact becomes a way of showing changes in the mechanical properties of amyloplast membranes.

Response: Thanks for the comment. We have addressed this question above.

There are typos in the manuscript, e.g. in the "methods" section (determination of the lipid composition) replace Kinete by Kinetex and CAN by ACN, NH4Ac by NH_4Ac

Response: Thanks for carefully reading the manuscript. These have been revised.

Finally, thanks again for your excellent comments that have significantly improved the quality of our manuscript.

Reviewer #3 (Remarks to the Author):

The revisions address most of my concerns. Due to the complexity of the genetics underlying this phenotype several key experiments that could shed light on the central hypothesis that non-polar carotenoids are responsible are probably not feasible in a reasonable time frame. These would include introducing carotenoid specific mutants, e.g. y1 and White cap,etc. The suppressor screen is a good alternative approach, but has not reached saturation. Pushing that screen to saturation would take considerable time.
In any case, regardless of whether their hypothesis is correct the implication of isoprenoid/carotenoid pathways in stability of amyloplast membranes is interesting.

Response: Thanks for your comments.
